# Photoswitching of Co(ii)-based coordination cages containing azobenzene backbones†

**DOI:** 10.1039/d4sc01575d

**Published:** 2024-05-03

**Authors:** Max B. Tipping, Lidón Pruñonosa Lara, Atena B. Solea, Larissa K. S. von Krbek, Michael D. Ward

**Affiliations:** a Department of Chemistry, University of Warwick Coventry CV4 7AL UK M.D.Ward@warwick.ac.uk; b Kekulé-Institut für Organische Chemie und Biochemie, Rheinische Friedrich-Wilhelms-Universität Bonn Gerhard-Domagk-Str. 1 53121 Bonn Germany larissa.vonkrbek@uni-bonn.de

## Abstract

Inclusion of photoswitchable azobenzene units as spacers into ditopic bridging ligands L^m^ and L^p^, containing two chelating pyrazolyl-pyridine termini, allows formation of metal complex assemblies with Co(ii) that undergo a range of light-induced structural transformations. One notable result is the light-induced conversion of a Co_2_(L^p^)_3_ dinuclear triple helicate (based on the *E* ligand isomer) to a *C*_3_-symmetric Co_4_(L^p^)_6_ assembly, assumed to be an edge-bridged tetrahedral cage, based on the *Z* ligand isomer. Another is the preparation of a series of Co_4_(L^m^)_6_ complexes, of which Co_4_(*E*-L^m^)_6_ was crystallographically characterised and consists of a pair of Co_2_(L^m^)_2_ double helicates connected by an additional two bridging ligands which span the pair of helicate units, giving a cyclic Co_4_ array in which one and then two bridging ligands alternate around the periphery. A set of Co_4_(L^m^)_6_ complexes could be prepared containing different ratios of *Z* : *E* ligand isomers (0 : 6, 2 : 4, 4 : 2 and 6 : 0) of which Co_4_(*Z*-L^m^)_2_(*E*-L^m^)_4_ was particularly stable and dominated the speciation behaviour, either during light-induced switching of the ligand geometry in pre-formed complexes, or when ligand isomers were combined in different proportions during the preparation. These examples of (i) interconversion between Co_2_L_3_ (helicate) and (ii) Co_4_L_6_ (cage) assemblies with L^p^, and the interconversion between a series of Co_4_L_6_ assemblies Co_4_(*Z*-L^m^)_n_(*E*-L^m^)_6−*n*_ with L^m^, constitute significant advances in the field of photoswitchable supramolecular assemblies.

## Abstract 2

Incorporation of a photoswitchable azobenzene unit into a ditopic bis(pyrazolyl-pyridine) ligand allows a range of reversible light-induced transformations between different cage assemblies.

## Graphical abstract

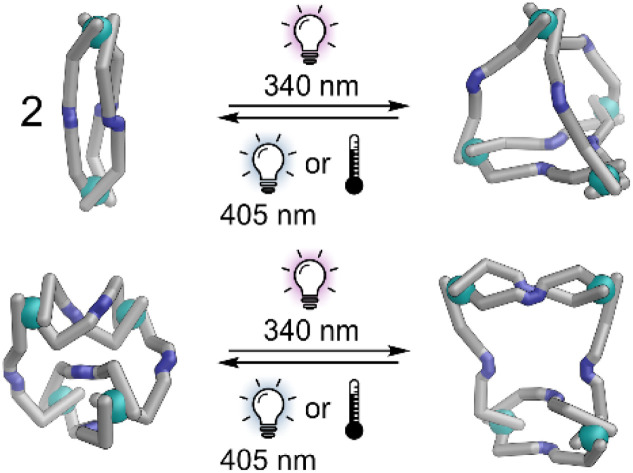

## Introduction

The ability of hollow metal–ligand coordination cages to accommodate small-molecule guests inside their central cavity^1^ can be made more valuable and widely applicable if the host–guest interactions can be controllably modulated, allowing for switchable guest uptake/release in response an external stimulus. These external stimuli may be chemical,^2^ electrochemical,^3^ or photochemical:^4^ light, in particular, is perceived as an excellent method for *in situ* manipulation of guest binding because of the precise tunability of wavelength and intensity, inherent cleanliness, and extreme ease of physical delivery.^5^

Many small molecules can undergo reversible structural changes when exposed to light, with one wavelength triggering a rearrangement and another wavelength triggering the reverse reaction – an obviously appealing way to modulate the structures of metal/ligand assemblies and hence control guest binding. These may include bond breaking and bond forming reactions that result in a significant chemical change in the unit concerned as shown by the photochromic dithienyl-ethene units from Irie and co-workers,^6^ which undergo reversible 6π-electron cyclisation/cyclo-reversion reactions (ring-closing and ring-opening) under light illumination at different wavelengths: this has been exploited by Clever and co-workers to make a Pd_2_L_4_ cage with ‘open’ and ‘closed’ forms which display different guest binding properties.^7^

Another popular photo-switching modality is provided by the *E/Z* isomerisation of stilbenes and azo-benzenes which can be exploited to allow control of the conformations of ligands where those units are incorporated into the backbone.^5*b*^ The first light-modulated coordination cage, based on azobenzene photoswitching units, was a Pd_12_L_24_ cage from Fujita's group: azobenzene units were bound to the concave side of each ligand (*i.e.* were inwardly-directed into the cavity) to form a 24-fold, endohedrally-functionalised ‘nanosphere’.^8^ This enabled the *in situ* manipulation of the hydrophobic interior surface of the cavity under light irradiation, causing the switchable uptake of the guest 1-pyrenecarboxaldehyde. Wu and co-workers prepared an azobenzene-containing ligand with anion-binding bis-urea end groups which, when combined with phosphate, formed an A_4_L_6_ tetrahedron; irradiation with 380 nm light transformed the *E*-ligand tetrahedron into a A_2_L_3_*Z*-ligand helicate, with concomitant release of a cavity-bound guest; this example was not reversibly photoswitchable however due to conformational restrictions associated with the rigid supramolecular structures.^9^ Beves and co-workers produced the first example of a visible-light responsive azobenzene-containing cage – a [Pd_2_L_4_]^4+^ lantern-shaped cage based on square-planar Pd(ii) ions which, when exposed to red light, isomerised to 2 equivalents of a PdL_2_ mononuclear complex. While the process was reversible with violet light, the photoisomerization yield and stability of the *Z*-ligand isomer were shown to improve with metal coordination.^10^ McConnell's and Herges's groups embedded the azobenzene photochromic unit into a cyclic diazocine unit, in which steric strain ensures that the *Z*-azo group is more stable; conformational changes induced by photoswitching of this diazocine ring enabled self-sorting of a mixture of regioisomers *via* formation and destruction of M_2_L_3_ helicates in the presence of Co(ii) ions.^11^

In this paper, we report how incorporation of the well-known azobenzene photochromic unit into our bis(pyrazolyl-pyridine) ligand system^12^ can be used as a basis to control the course of the self-assembly with metal ions. We have reported numerous examples of coordination cage complexes with ligands from this family,^12^ including some in which dynamic equilibria between cages of different nuclearity can be identified in solution.^13^ Here, the inclusion of the photochromic unit provides an additional light-based method to control the course of the self-assembly and allows, unusually, (i) switching between different complex forms such as dinuclear helicates and tetrahedral cages; and (ii) switching between isomeric forms of assemblies with the same composition.

## Results and discussion

### Synthesis, characterisation and photoswitching behaviour of ligands

**Scheme 1:**
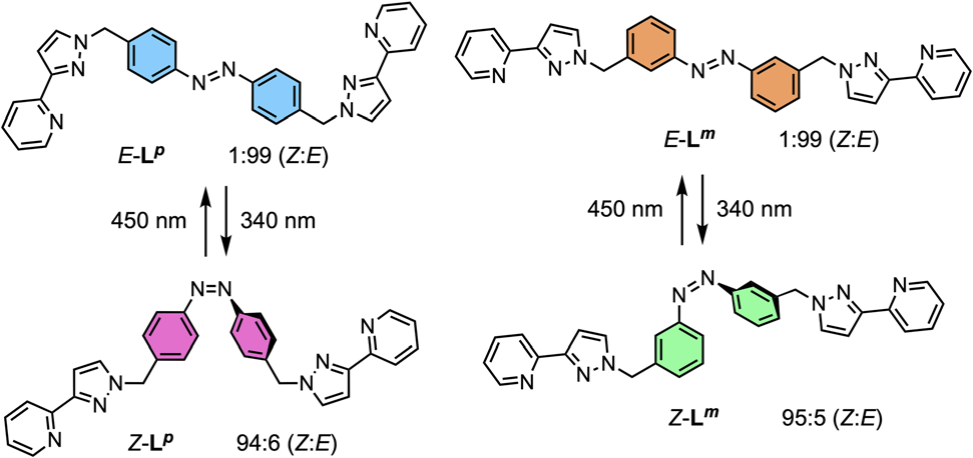
Ligands used in this paper and their isomeric forms.

The ligands used are shown in Scheme 1. The bridging ligand L^p^ was reported several years ago and used to prepare an Ag(i) metallamacrocycle: no photoswitching investigations were performed at that time.^14^ For this work we have also studied the isomeric ligand L^m^, which was prepared using the same general method as used for L^p^. Specifically, bromination of the methyl groups of 3,3′-dimethyl-azobenzene with *N*-bromo-succinimide in acetonitrile afforded 3,3′-bis(bromomethyl)-azobenzene in 70% isolated yield: reaction of this with two equivalents of 3-(2-pyridyl)pyrazole, under basic conditions to deprotonate the pyrazole NH group and in the presence of tetrabutylammonium iodide as a catalyst, afforded L^m^ in 75% isolated yield.

**Fig. 1:**
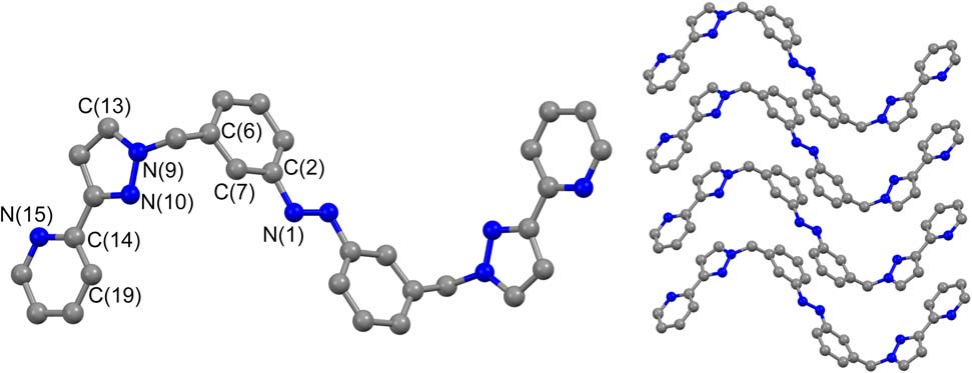
Molecular structure of L^m^ from crystallographic data, including the atomic labelling scheme and the packing arrangement.

Crystals of L^m^ were grown by diffusion of hexane vapour into a solution of the compound in ethyl acetate: the X-ray crystal structure is in Fig. 1 and shows the expected approximately transoid coplanar conformation of the pyrazolyl-pyridine as well as the *E* configuration of the N=N bond (length 1.234 Å). Extensive π–π interactions are apparent between centrosymmetric molecules of L^m^ in the stacked arrays.

**Fig. 2:**
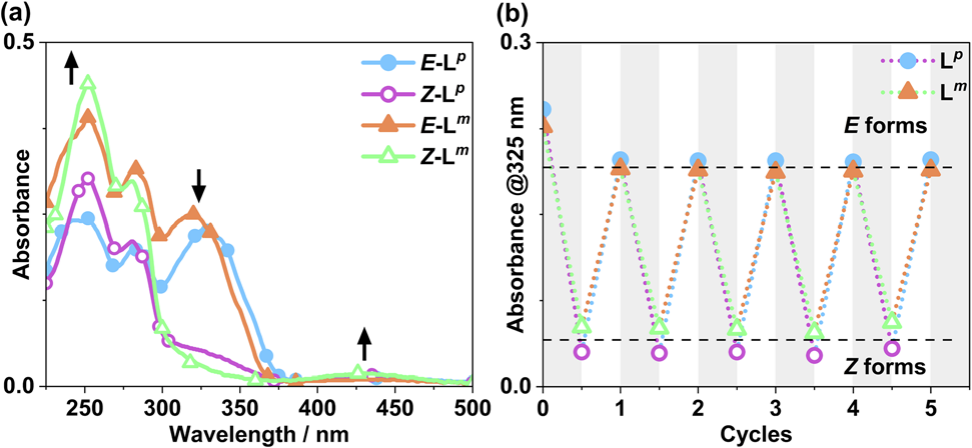
(a) Absorption spectra of 10 μM solutions of L^m^ and L^p^ in MeCN, pre (*E* form) and post (*Z* form) irradiation with 340 nm light. Arrows indicate changes in absorbance maxima. (b) Switching reversibility experiment monitoring absorbance of each ligand (10 μM) at 325 nm, after alternating irradiation with 340 nm and white light.

Following synthesis of the two ligands, it was important to confirm that they showed photo-switching behaviour. The UV/Vis spectra of both of the ligands in acetonitrile exhibited absorption bands at *ca.* 320 nm, corresponding to a π–π* transition, with the low-energy tail of the absorbance extending out to *ca.* 370 nm (Fig. 2a). Irradiation of 10 μM samples at 340 or 365 nm resulted in an immediate loss in intensity of the π–π* transition for the *E* isomer of each ligand, and an increased intensity for the weaker symmetry-allowed n–π* transition of the *Z* isomer of each ligand, at *ca.* 430 nm. Both azobenzene ligands had reached photostationary states (PSS) following 30 minutes of irradiation. The process could be mostly reversed by irradiation with white light, with a small difference in absorbance between the white-light-induced PSS and the thermally relaxed samples. In addition, the light-induced switching could be repeated for several cycles with no appreciable photobleaching (Fig. 2b).

**Fig. 3:**
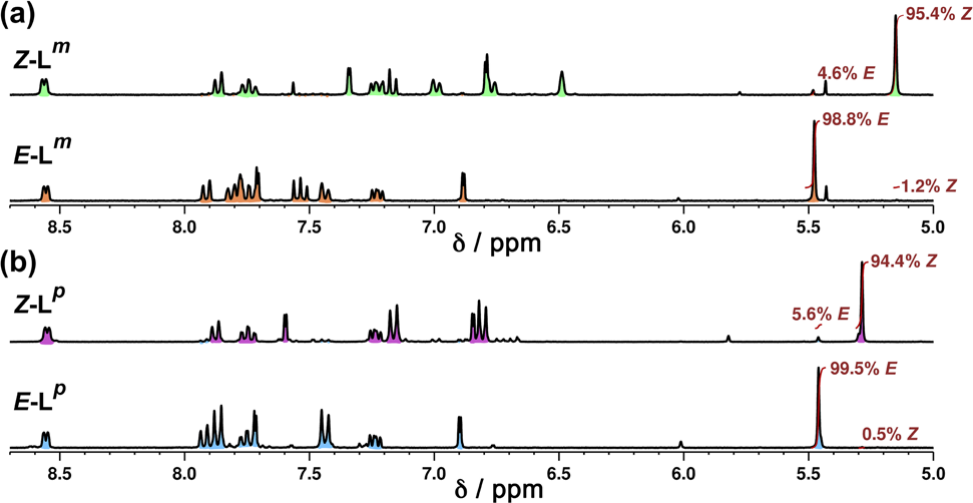
Stacked ^1^H NMR spectra (300 MHz, MeCN-d_3_, 10 μM, 298 K) of (a) L^m^ and (b) L^p^ showing changes between *E* forms (bottom) and predominantly *Z* forms (top), following photoirradiation at 340 nm, which generates a *Z* : *E* mixtures of 95 : 5 (L^m^) and 94 : 6 (L^p^).

NMR spectroscopy was used to quantify the PSSs of both ligands, using the integration ratios of the methylene protons which are well-separated from other signals (Fig. 3). Prior to irradiation of the sample, a solution of L^p^ in acetonitrile contained a mixture of 1% *Z* and 99% *E* isomers (note, all ratios will be presented as *Z* : *E*, with individual values to the nearest 1%). Following irradiation at 340 nm overnight, the sample composition was 94 : 6 *Z* : *E* (Fig. 3b). Similarly, an initial solution of L^m^ in acetonitrile containing a 1 : 99 *Z* : *E* isomeric mixture prior to irradiation switched to 95 : 5 *Z* : *E* post-irradiation (Fig. 3a).

Following determination of PSSs, photoconversion was studied using NMR spectroscopy during *in situ* illumination experiments whereby a quartz optical fibre was inserted into a sample tube such that the solution could be irradiated inside the spectrometer.^15^ Spectra were recorded every 17 seconds during both the forward and backward reactions. Due to the limited availability of LEDs it was not possible to irradiate the samples with 340 nm light during the NMR experiments, however 365 nm and 325 nm sources were available, of which the 365 nm source gave more photoswitching of L^p^ whereas the 325 nm source gave more photoswitching of L^m^: both ligands converted back from *Z* to *E* forms under 450 nm irradiation. Advantageously, the 325 and 365 nm LEDs are also much more powerful – 170 and 13 mW respectively – than the 1.7 mW 340 nm LED.

**Fig. 4:**
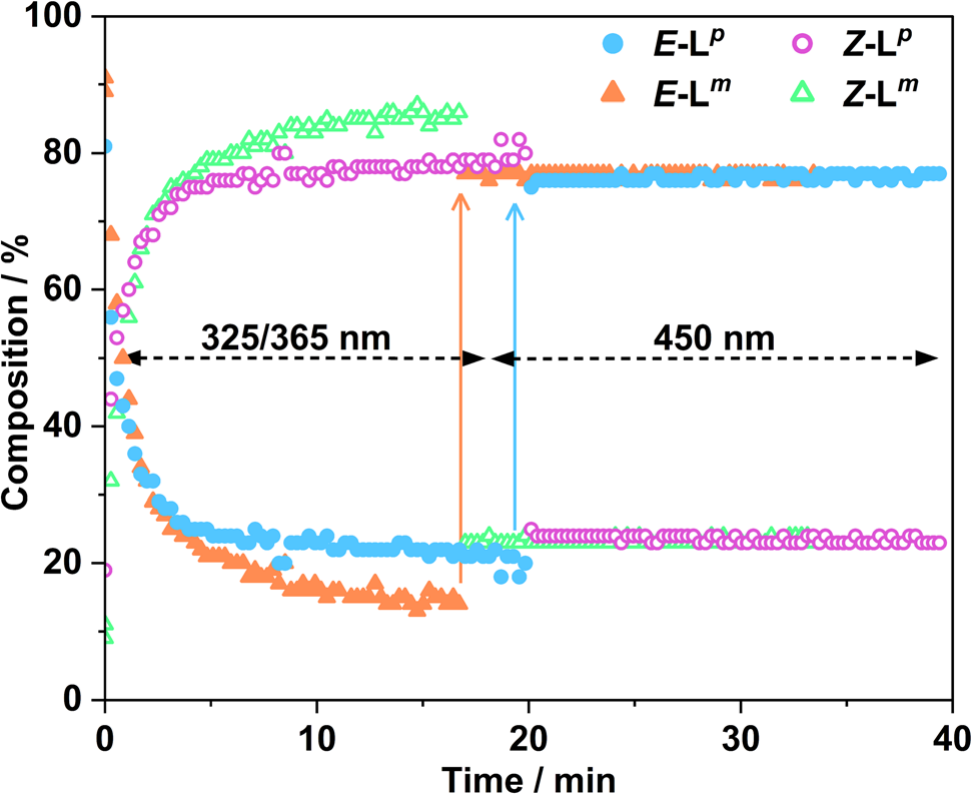
Changes in composition of *Z*/*E* mixtures of L^p^ (blue and pink for *E* and *Z* isomers, respectively) and L^m^ (orange and green for *E* and *Z* isomers, respectively) measured by *in situ* illumination ^1^H NMR (700 MHz, CD_3_CN, 298 K). A sample of *E*-L^m^ (2.0 mM) was irradiated with 325 nm light for *ca.* 17 minutes to induce the *E* to *Z* isomerisation; followed by illumination with 450 nm light for *ca.* 17 minutes, to switch the ligands back to the *E* configuration. A sample of *E*-L^p^ (2.0 mM) was irradiated with 365 nm light for *ca*. 20 minutes, with subsequent irradiation with 450 nm light for *ca.* 20 minutes. In both cases the slow change to the *Z* isomer during the first 17–20 minutes with the UV irradiation, and then the very fast change back to the *E* isomer following visible light irradiation, are clear.

Fig. 4 shows the resulting changes in isomeric composition of each ligand with time under irradiation as measured during the *in situ* NMR experiments. After approximately 5 minutes of irradiation with 365 nm light, isomerisation of L^p^ showed no further changes, with a PSS_365nm_ ratio of 79 : 21 *Z* : *E* being obtained under these conditions. Subsequent exposure to 450 nm light caused a fast return to the original composition, with 20 minutes of additional irradiation causing no further changes. The much shorter timescale for the reverse reaction is likely due to the higher power of the 450 nm LED (440 mW, compared to 170 mW for the 365 nm LED) and not necessarily to any differences in isomerisation mechanisms.^16^ In a similar way a solution of L^m^, after approximately 20 minutes of irradiation with 325 nm light, achieved a PSS_325nm_ ratio of 85 : 15 *Z* : *E* isomers under the same conditions. Again, the reverse reaction with 450 nm light was fast with all changes complete after 17 seconds.

This sample did not return completely to its original state however, instead consisting of a PSS_450nm_ ratio of 22 : 78 *Z* : *E*, so for L^m^ the photo-isomerisation is not fully reversible under 450 nm light irradiation (Fig. 4).

**Fig. 5:**
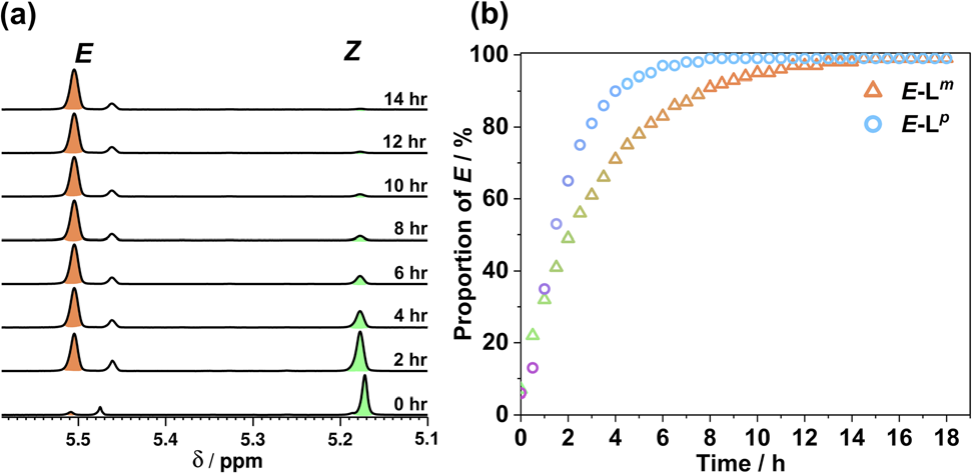
(a) ^1^H NMR spectra (400 MHz, CD_3_CN, 338 K) indicating the change in the methylene proton signals of (predominantly) *Z*-L^m^ peaks following heating at 65 °C in CD_3_CN to effect thermal relaxation back to *E*-L^m^; (b) proportion of *E*-L^m^ and *E*-L^p^ with time during this heating process.

Usefully, azobenzenes may also undergo specifically the *Z* to *E* isomerisation by thermal relaxation. To test this, solutions of L^m^ and L^p^ in acetonitrile were first irradiated until PSSs were reached, producing a 93 : 7 and 94 : 6 *Z* : *E* ratio, respectively. Fig. 5 shows how the composition of isomers in solution varies after heating this sample to 65 °C for 18 hours. Both ligands showed complete conversion back to the *E* form with a thermal half-life *τ*_1/2_ of 2.36 h for L^m^ and 1.38 h for L^p^, demonstrating an alternative thermal method for isomerisation of the *Z* form back to the *E* form.

### Metal complexes with L^p^: synthesis and characterisation

Reaction of L^p^ (1.5 equivalents) with 1 equivalent of an appropriate metal salt [Co(BF_4_)_2_ or Zn(BF_4_)_2_] at 60 °C overnight in MeOH afforded a precipitate which was washed with methanol, CH_2_Cl_2_ and diethyl ether to remove any unreacted metal salts and ligand. The 1.5 : 1 ligand : metal ratio reflects the generally six-coordinate preference of the metal ion and the bis-bidentate nature of the ligands, and this ligand : metal ratio routinely appears in this family of complexes based on bis(pyrazolyl-pyridine) ligands.^12^

**Fig. 6:**
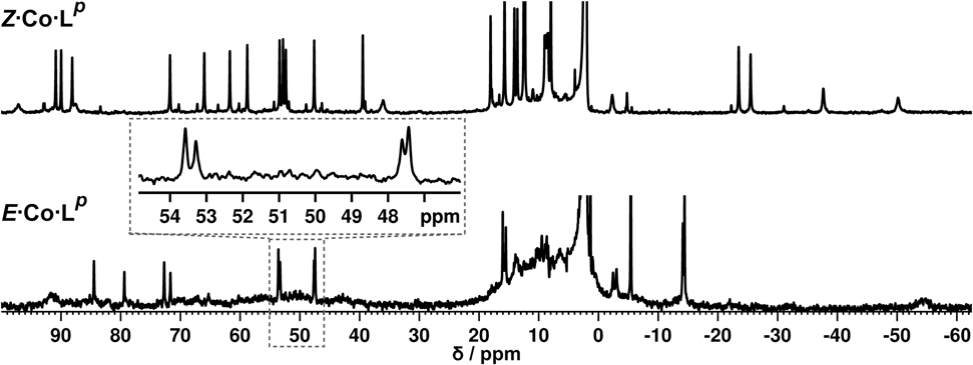
(Top) ^1^H NMR spectrum (300 MHz, CD_3_CN, 298 K) of *Z*·Co·L^p^, prepared by irradiation at 340 nm of a solution of *E*·Co·L^p^. (Bottom) ^1^H NMR spectrum (300 MHz, CD_3_CN, 298 K) of *E*·Co·L^p^. An expansion of the 47–54 ppm region makes clear the presence of two closely-spaced signals: this is also apparent in the signal at −14 ppm.

We denote the Co(ii) complex that was prepared with the (predominantly) *E* form of ligand as *E*·Co·L^p^. The high-resolution electrospray ionisation (ESI) mass spectrum showed a sequence of signals corresponding to the species [Co_2_(L^p^)_3_(BF_4_)_n_]^(4−n)+^ (*n* = 0, 1, 2) suggesting that the complex formed is [Co_2_(L^p^)_3_](BF_4_)_4_ with three bridging ligands spanning two Co(ii) centres to give a triple-stranded structure which could be either a helicate or a mesocate.^17^ The ^1^H NMR spectrum, characteristically dispersed over a range of nearly 200 ppm due to the paramagnetism of high-spin Co(ii), clearly contains major and minor signals (Fig. 6): but the number of signals associated with the major component is consistent with one half of one ligand environment being unique, *i.e.* all three ligands are the same and have twofold symmetry, which (in conjunction with the ESI-MS data) is consistent with a dinuclear triple helicate structure for *E*·Co·L^p^.

**Fig. 7:**
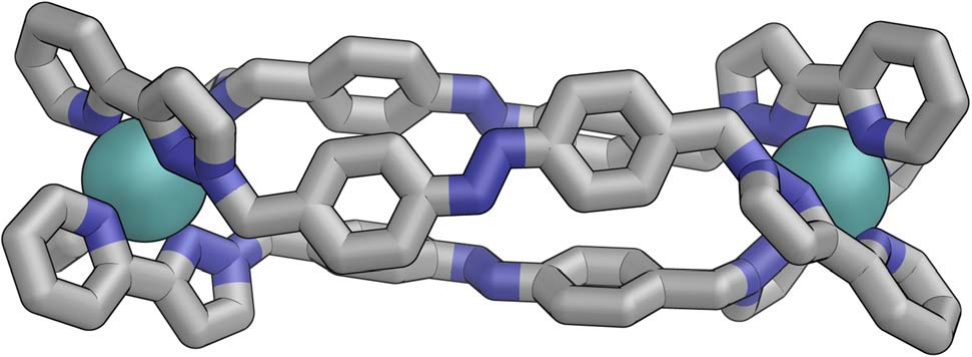
MM2-optimised model of *E*·Co·L^p^ showing a dinuclear, triple-helicate with D_3_ symmetry.

As no *X*-ray quality crystals could be obtained for *E*·Co·L^m^, a minimum-energy structure was calculated using an MM2 force-field (Fig. 7). The two metal centres possess *fac* tris-chelate coordination geometries and the three ligands adopt continuous spiral conformations, characteristic of helicates. The resulting D_3_ symmetry is consistent with the ^1^H NMR spectrum of the major solution species (Fig. 6) with one half of a ligand environment being unique.

**Fig. 8:**
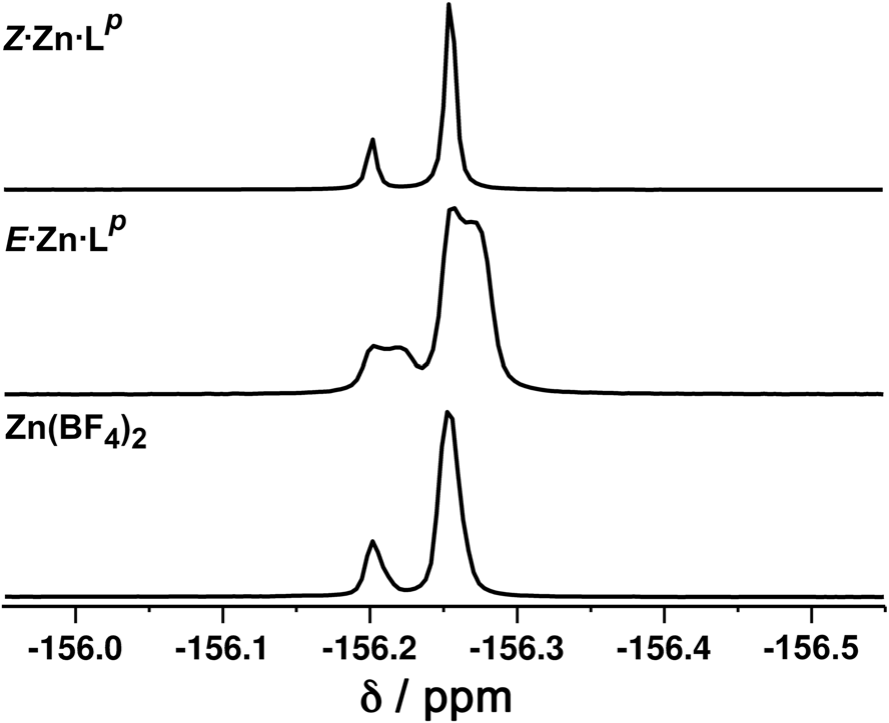
^19^F NMR spectra (400 MHz, CD_3_CN, 298 K) of (bottom) Zn(BF_4_)_2_ showing the presence of only one anion environment (the two separate signals arise from the ^10^B and ^11^B isotopomers which give slightly different chemical shifts for attached ^19^F atoms); (middle) *E*·Zn·L^p^ showing by the extra splitting the presence of two BF_4_^−^ anion environments in the complex; and (top) *Z*·Zn·L^p^ showing the presence of only one anion environment.

Close examination of the ^1^H NMR spectrum reveals that some of the signals are split into two closely-spaced components of comparable intensity (particularly the signals at *ca.* −14, 48 and 53 ppm, see inset in lower part of Fig. 6 showing expansions of the latter two), which could indicate the presence of slightly different environments associated with a guest (such as a counter-anion) binding to the helicate, possibly in the central cavity,^18^ such that there are separate signals for ‘free’ and ‘guest-bound’ helicate. Indirect evidence for this suggestion comes from preparing the analogous Zn(ii) complex *E*·Zn·L^p^ which has the composition (from high-resolution ESI-MS) [Zn_2_(L^p^)_3_](BF_4_)_4_ and is likely to be isostructural to the Co(ii) analogue. The ^19^F NMR spectrum of *E*·Zn·L^p^ (Fig. 8) showed that the signals associated with the fluoroborate anions (separate ^19^F signals for the ^10^B and ^11^B isotopomers are clearly visible) are split into two closely-spaced components, consistent with the anions being in two different environments: such splitting is not present in the ^19^F NMR spectrum of Zn(BF_4_)_2_ (Fig. 8).‡‡We used the Zn(ii) complex for this experiment because paramagnetic broadening in the Co(ii) complex meant that the closely-spaced signals for the two forms of the complex (with free or bound anion) could not be resolved. The two component spectrum suggests that BF_4_^−^ anions from *E*·Zn·L^p^ are found free in solution or are associated with the [Zn_2_(L^p^)_3_]^4+^ core, with any free/bound anion exchange being slow on the NMR timescale. We were unable to obtain X-ray quality crystals of these complexes but the NMR evidence for anion interaction with the complex cation is clear, though the close overlap of the ^19^F signals precludes accurate integration. Association of anions to the surface of cages formed using ligands of this family has been firmly established crystallographically and also underpins the solution catalytic activity of this family of cages.^12,19^ A possible (calculated) structure for *E*·Co·L^p^ containing one fluoroborate anion inside the triple helicate is in ESI, Fig. S51.†

### Metal complexes with L^p^: photoswitching

Irradiation of a CD_3_CN solution of *E*·Co·L^p^ with 340 nm light results in the major component of the ^1^H NMR spectrum becoming significantly more complex, with >30 new ^1^H signals (the major component of the spectrum) appearing, indicating formation of a new species with lower internal symmetry (Fig. 6, top). Significantly, the sharp signals associated with *E*·Co·L^p^ are now completely absent. On the basis that irradiation at this wavelength converts the *E* form of L^p^ to the *Z* form, we denote this new species *Z*·Co·L^p^. The high-resolution ESI-MS of this solution revealed a new series of signals corresponding to the species [Co_4_(L^p^)_6_(BF_4_)_*n*_]^(8−*n*)+^ (*n* = 2, 3, 4, 5), suggesting that an M_4_L_6_ tetrahedral cage had formed. The number of new ^1^H NMR signals is consistent with the presence of two different ligand environments, each with no internal symmetry: even if we cannot observe all 48 expected signals (because some are overlapping, or paramagnetically highly broadened, or obscured by residual solvent/water signals in the 0–10 ppm region) the number is clearly larger than the 24 signals that would arise from a structure associated with one independent, non-symmetrical ligand environment. This, together with the ESI-MS data, implies formation of an M_4_L_6_ cage that has *C*_3_ symmetry because one vertex is different from the other three (which are equivalent) such that there is an M^A^M_3_^B^ arrangement of metal types. This can arise when one metal centre has a *fac* tris-chelate coordination geometry and the other three have *mer* tris chelate coordination geometries, as we have reported;^20^ or it can arise when one metal centre has Λ chirality and the other three have Δ (or *vice versa*).^21^ We note in the NMR spectrum (Fig. 6, top) the presence of a set of much weaker signals, associated with a minor component. As the ES mass spectrum of *Z*·Co·L^p^ indicates the formulation Co_4_(L^p^)_6_(BF_4_)_8_ with no evidence for other cage sizes, a plausible explanation for the minor peaks is formation of an alternate cage isomer (*T* and *S*_4_ isomers for tetrahedral cages are also possible).^21^

**Fig. 9:**
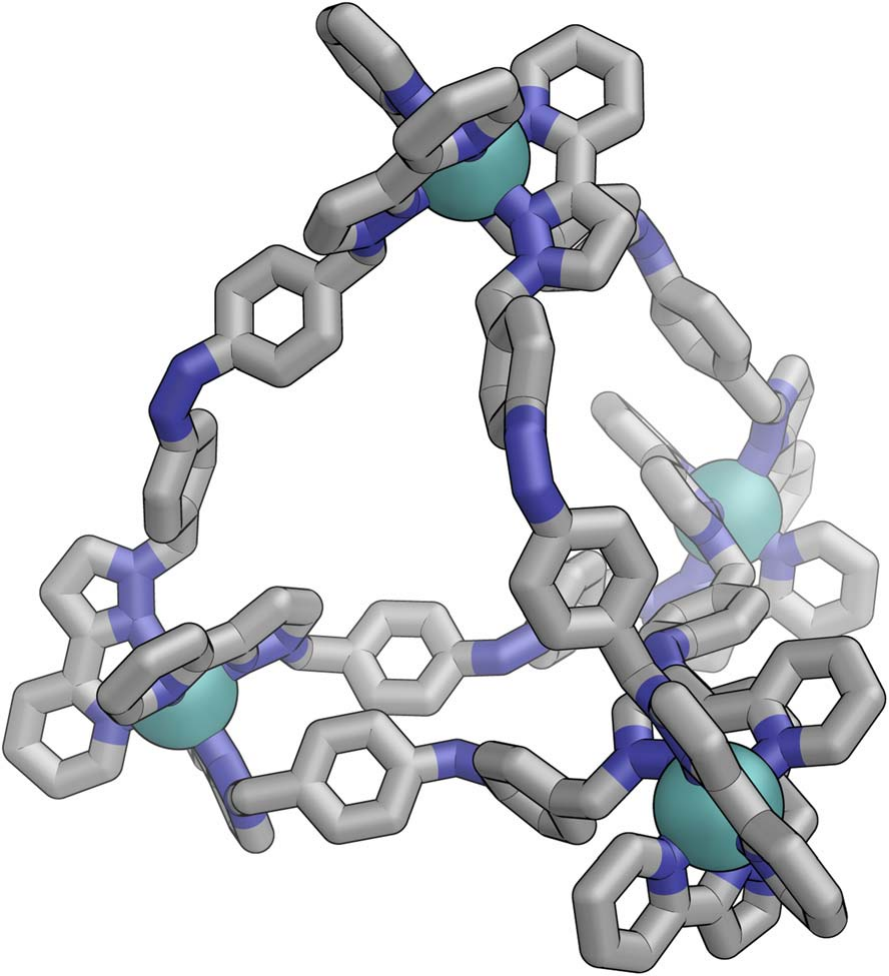
GFN-xTB generated structure of the C_3_ symmetric M_4_L_6_ tetrahedral cage *Z*·Co·L^p^. The structure contains two distinct ligand environments, with one ligand environment spanning *fac* (top metal ion in Figure) and *mer* (other three metal ions at base of Figure) metal vertices, and the other spanning two *mer* vertices but with no internal symmetry due to the cage chirality.

Fig. 9 shows the GFN-xTB optimised geometry^22^ of a *C*_3_ symmetric M_4_L_6_ tetrahedral cage, *Z*·Co·L^m^, with one *fac* and three *mer* vertices. The resulting structure possesses, as required by the ^1^H NMR spectrum, two distinct ligand environments (with one ligand spanning *fac* and *mer* tris-chelate metal vertices; and the other spanning two *mer* vertices but with no internal symmetry due to the chirality of the cage), which follows a previously-observed structure in this cage family.^20^ Such *C*_3_-symmetric M_4_L_6_ tetrahedra are less common than *T*-symmetric M_4_L_6_ tetrahedra in which all four metal centres are identical and are usually *fac* tris-chelates.

We note that there is no splitting of signals indicative of slow-exchange guest binding in the ^1^H NMR spectrum of *Z*·Co·L^p^, or in the ^19^F NMR spectrum of the Zn analogue (*Z*·Zn·L^p^) (Fig. 8). This could be due to fluoroborate anions binding to the more accessible cavity of the tetrahedron in fast exchange on the NMR timescale,^20^ or no binding occurring at all due to restructuring of the assembly from helicate to tetrahedron triggering guest release.

**Fig. 10:**
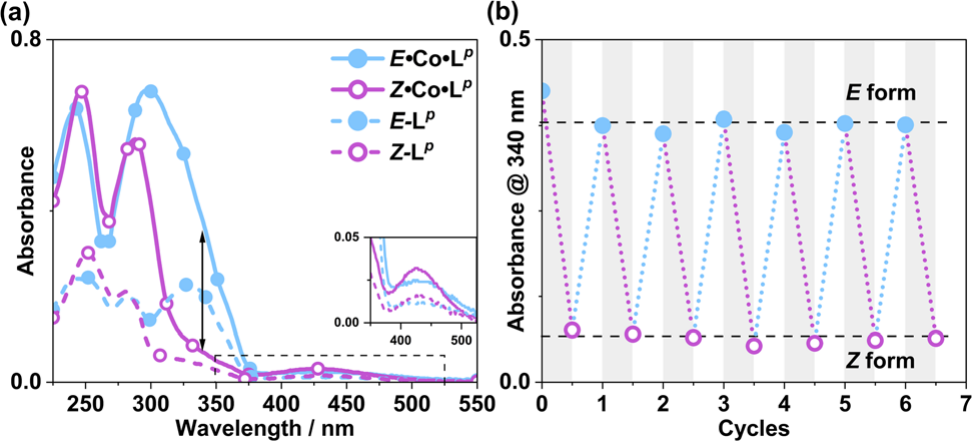
(a) UV/Vis spectra of *E*·Co·L^p^ and *Z*·Co·L^p^ (MeCN, 13 μg ml^−1^), as well as *E*-L^p^ and *Z*-L^p^ (MeCN, 10 μM). (b) Switching stability experiment monitoring the absorbance at 340 nm [black arrow in part (a)] of a 13 μg ml^−1^ sample after alternating irradiation with 340 nm for 30 minutes and then white light for 30 minutes, to switch between *E*·Co·L^p^ and *Z*·Co·L^p^ complexes.

The photo-conversion of *E*·Co·L^p^ to *Z*·Co·L^p^ (Co_2_L_3_ form to Co_4_L_6_ form) proceeded to completion within the detection limits of NMR spectroscopy, with complete loss of *E*·Co·L^p^. This photoswitching is more effective (in terms of completeness) than was observed for free L^p^ in acetonitrile (Fig. 10), which may be ascribed to the greater separation between the π–π* and n–π* bands in the absorption spectrum (13 μg ml^−1^Co·L^p^ and 10 μM L^p^)(Fig. 10a) and also, possibly, to a greater steric barrier to relaxation when the ligand is incorporated into a cage structure compared to being free. Similarly, the reverse photoswitching of *Z*·Co·L^p^ back to *E*·Co·L^p^ (Co_4_L_6_ form back to Co_2_L_3_ form) went to completion on irradiation with white light, indicating complete chemically reversible photoswitching between Co_2_L_3_ (likely a triple helicate) and Co_4_L_6_ (likely a C_3_-symmetric tetrahedral cage) species.

**Fig. 11:**
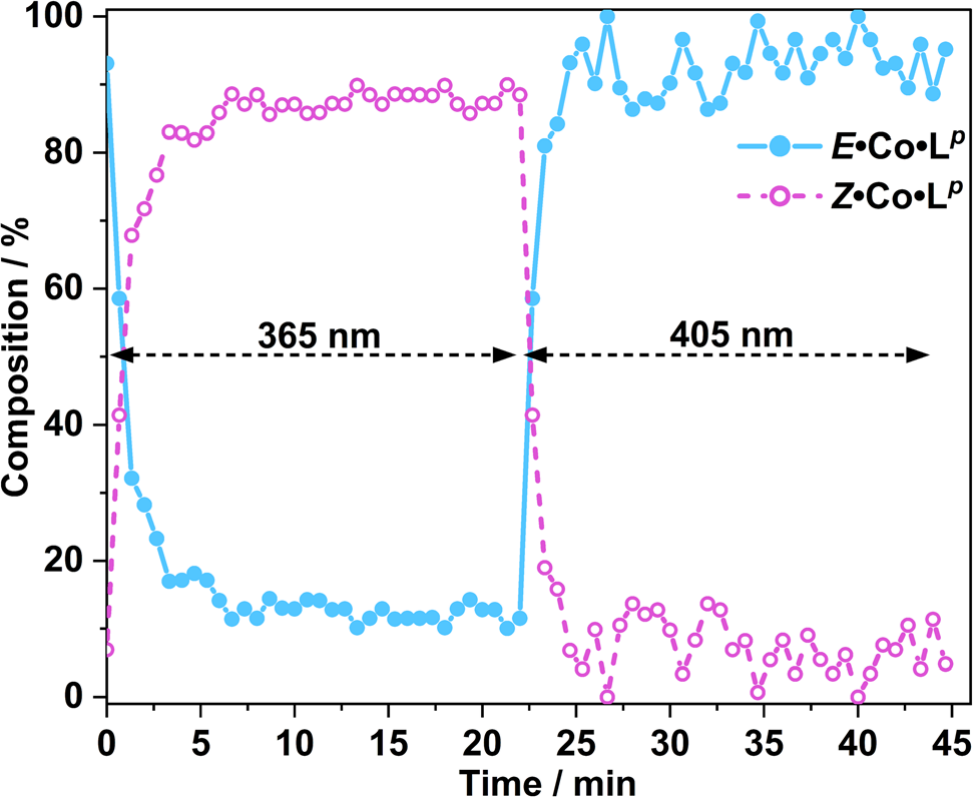
Photoswitching of a solution of *E*·Co·L^p^ to *Z*·Co·L^p^ and back, using 365 nm and then 405 nm irradiation respectively, monitored by *in situ*^1^H NMR spectroscopy (700 MHz, CD_3_CN, 298 K); see main text. The light blue trace shows the initial solution containing 3 : 97 *Z* : *E* complexes converting over several minutes to a PSS containing *ca.* 90 : 10 *Z* : *E* forms under UV irradiation, followed by rapid conversion back to the starting composition under 405 nm light excitation.

*In situ* NMR measurements during illumination were used to probe the kinetics of this metal complex photoswitching, again using a quartz optical fibre inserted into the sample in the NMR cavity (Fig. 11 and S48/49†).^15^^1^H NMR spectra were recorded every 40 seconds for 20 minutes under irradiation with 365 nm and subsequently 405 nm light. Given the high dispersion of NMR signals arising from the paramagnetism of high-spin Co(ii), a relatively narrow NMR spectral width (50–75 ppm) containing two (for *E*·Co·L^p^) or three (for *Z*·Co·L^p^) characteristic signals was used to provide good resolution of the signals in those selected windows. To help compensate for any uncertainty in integration of the broad signals associated with paramagnetism, integrals were recorded for all of the signals associated with each compound in the relevant window, and then averaged, with the resulting average being used to determine the ratio of isomers in solution.

Prior to irradiation, the sample of Co·L^p^ used was prepared using the as-isolated ligand L^p^ (3 : 97, *Z* : *E*), *i.e.* predominantly the M_2_L_3_ complex containing the *E* form of the ligand, *i.e. E*·Co·L^p^. Following approximately 5 minutes of irradiation a PSS was reached comprising 90 : 10 *Z* : *E*Co·L^p^ (Fig. 11). Subsequent exposure to 405 nm light showed complete reversion to the original mixture after 3 minutes. Moreover, UV/Vis spectroscopy could also be used to show cycling between the two species (Fig. 10): the spectrum of *E*·Co·L^p^ showed a small blue shift of the ligand's π–π* transition following coordination to Co(ii), from 327 nm to 305 nm, which ensured a better separation from the n–π* transition for *Z*·Co·L^p^ than is possible for the free ligands. Light-induced switching between the M_2_L_3_ complex *E*·Co·L^p^ (presumed triple helicate) and the M_4_L_6_ complex *Z*·Co·L^p^ (presumed *C*_3_-symmetric cage isomer) could be repeated for many cycles with no appreciable photobleaching (Fig. 10b).

**Fig. 12:**
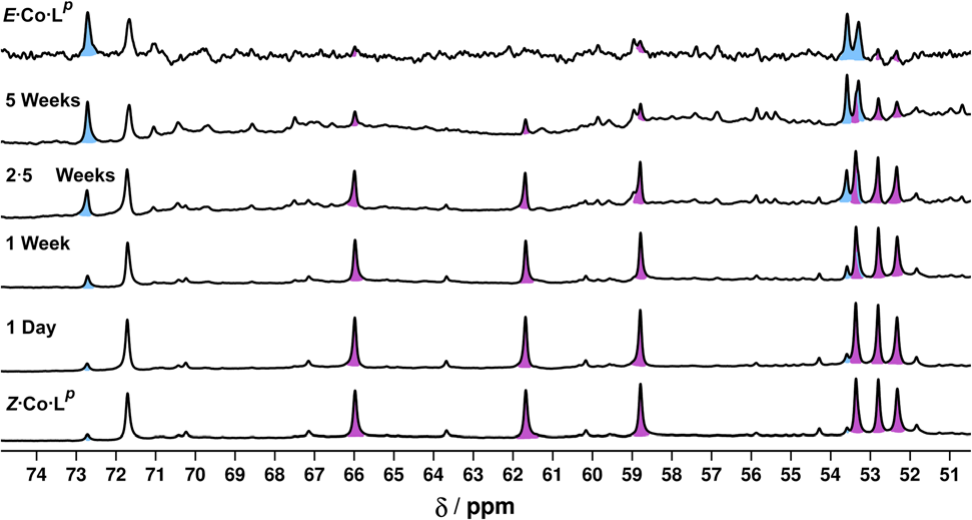
Series of ^1^H NMR spectra (400 MHz, CD_3_CN, RT) showing the slow thermal conversion of *Z*·Co·L^p^ (bottom spectrum) back to *E*·Co·L^p^ (top spectrum) over 5 weeks. The top and bottom spectra match those in Fig. 6.

Finally, we note that *Z*·Co·L^p^ showed good thermal stability, taking approximately 5 weeks to return to *E*·Co·L^p^ in solution at ambient temperature (Fig. 12): the high kinetic inertness of coordination cages compared to mononuclear species, even when based on individually labile metal complex vertices, is well known.^13,23^ We also note that thermal relaxation is expected to be slower than light-induced isomerisations which are – effectively – performed under forcing non-equilibrium conditions.^24^

### Metal complexes with L^m^: synthesis and characterisation

These complexes were prepared in exactly the same way as the complexes of L^p^, by reaction of 1.5 equivalents of L^m^ (in the as-isolated 3 : 97 *Z* : *E* form) with one equivalent of Co(BF_4_)_2_ or Zn(BF_4_)_2_ in methanol overnight. After evaporation of solvent to give a solid product, any unreacted ligand and metal salt were removed with ethyl acetate and diethyl ether washes. High-resolution ESI-MS of the Co(ii) product, which we denote *E*·Co·L^m^, showed a sequence of signals for the species [Co_4_(L^m^)_6_(BF_4_)_*n*_]^(8−*n*)+^ (*n* = 2, 3, 4, 5), suggesting that an M_4_L_6_ complex (possibly, but not necessarily, a tetrahedral cage) had formed. The mass spectra however also showed signals corresponding to [Co_2_(L^m^)_3_(BF_4_)_*n*_]^(4−*n*)+^ (*n* = 1, 2, 3, 4): and whilst some of these will have the same *m/z* values as signals from [Co_4_(L^m^)_6_(BF_4_)_*n*_]^(8−*n*)+^ and hence overlap (see ESI†), the different isotopic spacings make the presence of [Co_2_(L^m^)_3_(BF_4_)_*n*_]^(4−*n*)+^ in the sample quite clear, though whether this is a separate species or just a fragment induced by ESI conditions is not known. The high-resolution ESI-MS of *E*·Zn·L^m^ showed a signal corresponding to [Zn_4_(L^m^)_6_(BF_4_)_5_]^3+^.

**Fig. 13:**
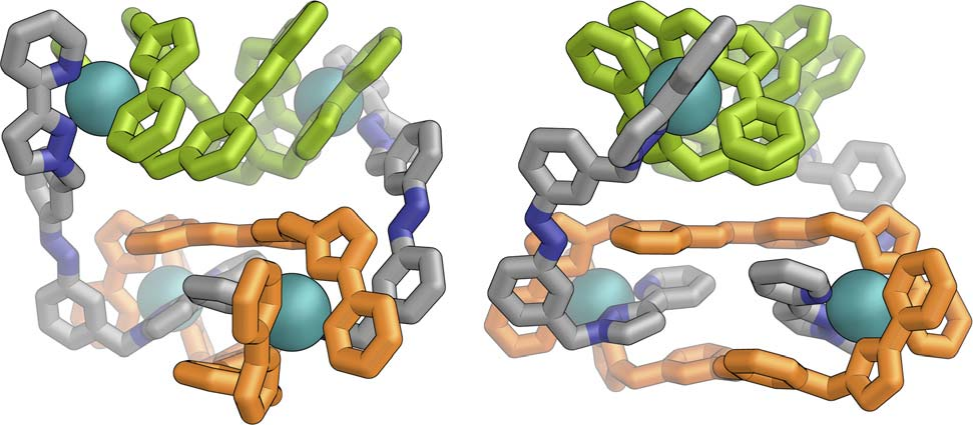
Two views of the molecular structure of the M_4_L_6_ complex cation of *E*·Co·L^m^ from an X-ray crystal structure determination, with different types of ligand environment highlighted in green or orange (within the two M_2_L_2_ helicate units), or grey (cross-linking the two helicate units).

Slow cooling of a solution of *E*·Co·L^m^ in methanol afforded X-ray quality crystals; the molecular structure of the complex is shown in Fig. 13. The M_4_L_6_ composition as shown by ESI-MS is confirmed. The structure is not that of a conventional edge-bridged tetrahedral cage, but consists of a pair of (crystallographically inequivalent) M_2_L_2_ double helicate units which are connected at each end by an additional bridging ligand spanning the two helicate units. The connectivity is therefore that of a closed (non-planar) M_4_ cycle with alternately 1 and then 2 bridging ligands spanning successive edges – a structural type that has been reported before.^25^ The ligands are coloured as three pairs in Fig. 13 for clarity, with one M_2_L_2_ unit having ligands coloured green, the other having ligands coloured orange, and the cross-piece ligands connecting the helicates coloured grey. Note that the same colouring does not imply crystallographic equivalence as the complex molecule has no internal crystallographic symmetry, with an entire molecule in the asymmetric unit. All metal centres have a *mer* tris-chelate coordination geometry and all have the same optical configuration (Δ in the figure shown, with the enantiomeric assembly also present in the other half of the *P*1̄ unit cell).

**Fig. 14:**
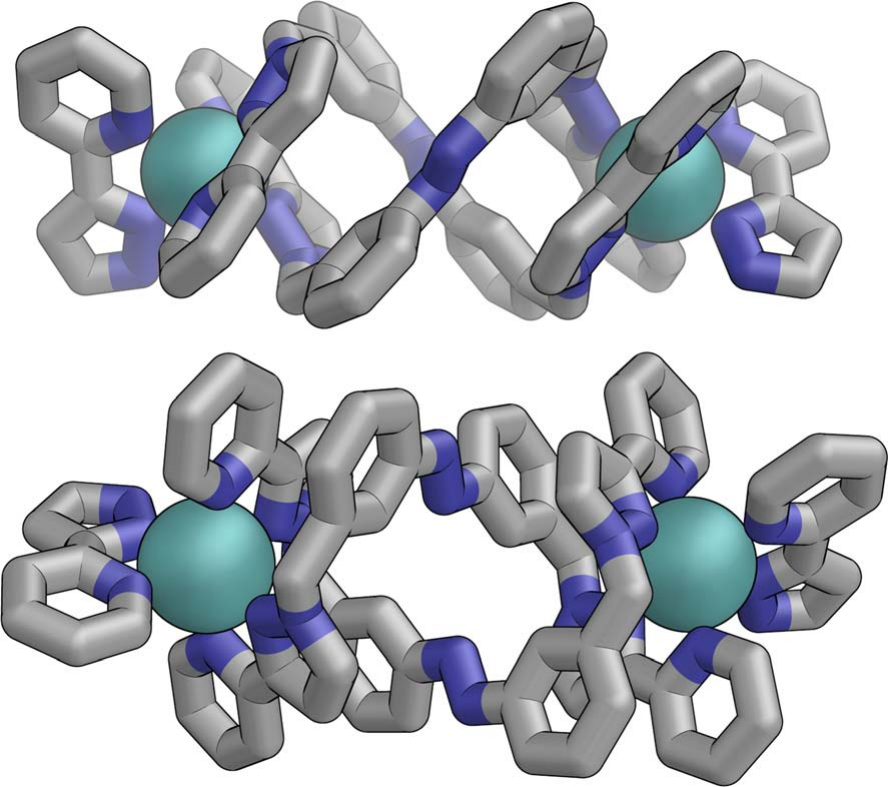
Structure of the M_2_L_2_ double-helicate unit coloured green in Fig. 13, with approximate (non-crystallographic) *C*_2_ internal symmetry in which the two ligands are essentially identical but conformational details two ends of each ligand are different (*i.e.* both adopt the same ‘head to tail’ arrangement within the M_2_L_2_ unit).

The flexibility of the ligands associated with the –CH_2_– spacer units, as well as rotation of the aromatic rings about the Ph–N bonds, provide substantial conformational freedom. The two ligands coloured green in Fig. 13 have a conformation in which the internal twofold symmetry of each ligand is lost, *i.e.* the ‘head’ and ‘tail’ ends are different, but the head-to-tail disposition of the pair of similar (but not crystallographically equivalent) ligands provides approximate local *C*_2_ symmetry for this helical unit (Fig. 14).

**Fig. 15:**
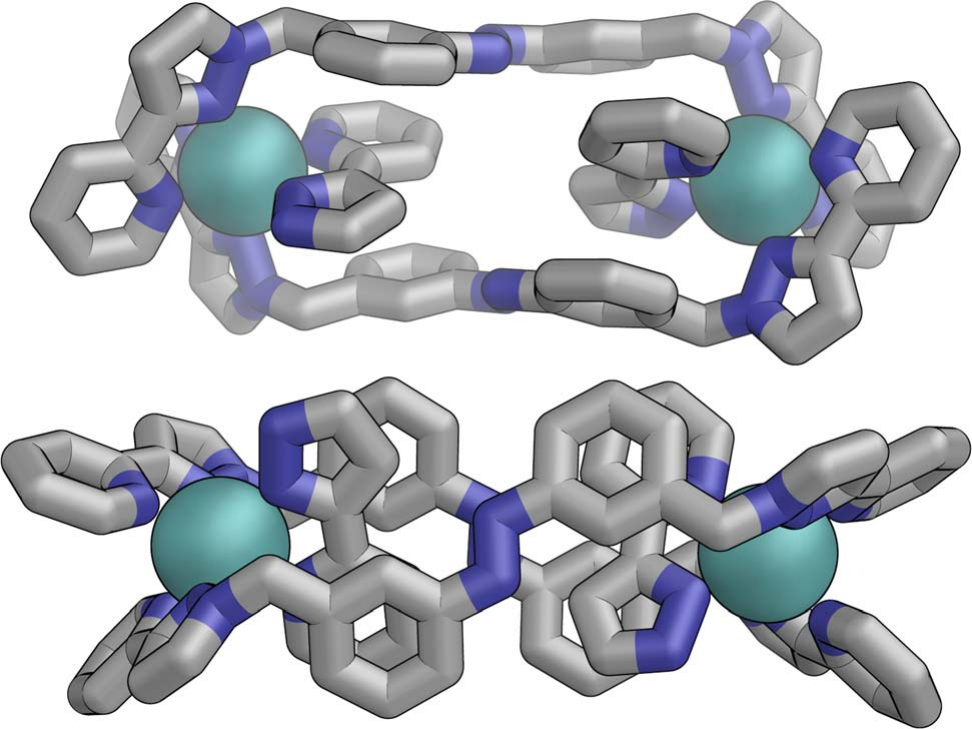
Structure of the M_2_L_2_ double-helicate unit coloured orange in Fig. 14, with each ligand having approximate (non-crystallographic) *C*_2_ internal symmetry with each end the same: the two ligands are essentially identical, giving local *D*_2_ symmetry for the M_2_L_2_ unit.

In contrast the pair of orange-coloured ligands in the other M_2_L_2_ helicate have a conformation in which both retain (non-crystallographic) internal *C*_2_ symmetry and are also very similar to one another, giving this fragment local *D*_2_ symmetry (Fig. 15), though this is removed by one face of that helicate being closer to the other M_2_L_2_ unit, pointing ‘into’ the centre of the complex, than the other which lies on the exterior surface. In both M_2_L_2_ helicate units we can see inter-ligand π-stacking with phenyl rings of one ligand stacked with electron-deficient, coordinated, pyrazolyl-pyridine units from other ligands. The grey-coloured ligands connecting the two helicate fragments have no internal symmetry. The Zn(ii) analogue *E*·Zn·L^m^ was also crystallographically characterised and is isostructural, but provided significantly poorer quality data: accordingly it is not included here but we just note that it is isostructural/isomorphous to *E*·Co·L^m^.

**Fig. 16:**
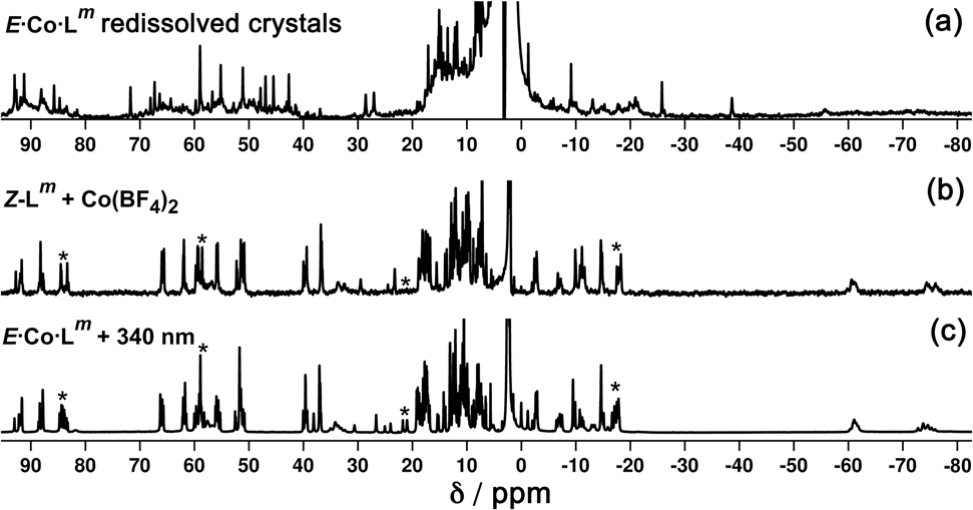
^1^H NMR spectra (300 MHz, CD_3_CN, 298 K) of (a) redissolved crystals of *E*·Co·L^m^; (b) *Z*·Co·L^m^ prepared by combination of *Z*-L^m^ with Co(BF_4_)_2_; and (c) *Z*·Co·L^m^ prepared by irradiation of a sample of *E*·Co·L^m^ at 340 nm. Comparison of signals labelled * between parts (b) and (c) show additional complexity in the spectrum of the sample generated by photoswitching.

In solution, allowing for adoption of formally higher symmetry due to relaxation from what is observed in the crystal structure, the cation of *E*·Co·L^m^ could display twofold symmetry, with a single *C*_2_ axis bisecting the two helicates: this would lead to three independent ligand environments – the orange, green and grey ligand types shown in Fig. 14 – and, in principle, 72 different ^1^H NMR signals. The observed ^1^H NMR spectrum (from redissolved crystals) reveals a complicated mixture of signals of different intensities that is not susceptible to simple analysis (Fig. 16a): there are clearly major and minor components present whose relative intensities were concentration dependent, with the weaker signals becoming relatively more intense compared to the stronger signals at higher concentrations, possibly indicating aggregation into a larger structure. This is consistent with what would be expected for a combination of M_2_L_3_ and M_4_L_6_ (and perhaps other) species in solution, *cf.* the ESI mass spectrum which revealed the presence of both M_2_L_3_ and M_4_L_6_ species, and it is to be expected on the basis of the Le Chatelier principle that the larger assembly dominates at the high local concentrations when crystal nucleation occurs. We have seen concentration-dependence in the speciation behaviour of other metal/ligand assemblies from this general family of ligands,^13^ and we note that McConnell and Herges,^26^ and Clever,^27^ have similarly observed complex mixtures of species forming in solution using bridging ligands incorporating diazocene units into the spacers.

### Metal complexes with L^m^: preparation and identification of mixed-ligand-isomer complexes

**Fig. 17:**
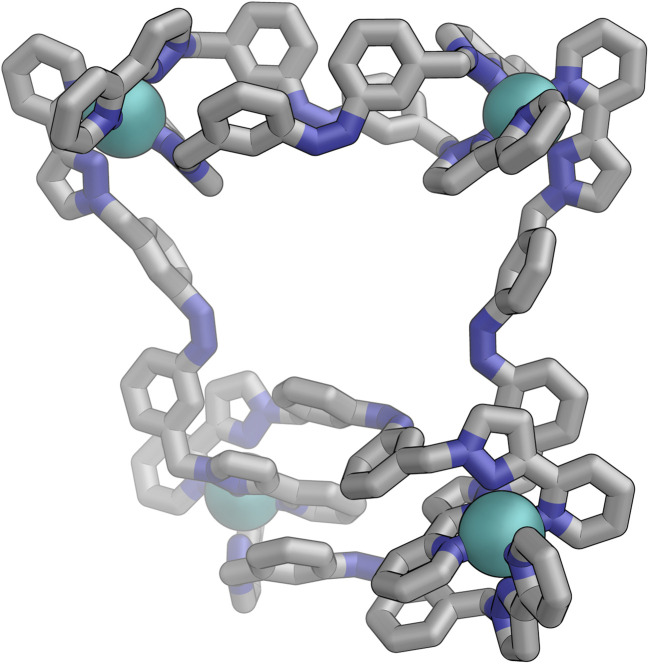
GFN-xTB generated structure of the M_4_L_6_ complex cation of *Z*·Co·L^m^ comprising two M_2_L_2_ helicate units joined by two cross-linking ligands, with no internal symmetry.

Following photo-irradiation of *E*·Co·L^m^ with 340 nm light to induce *E* to *Z* isomerisation of the ligands, ESI-MS showed that signals corresponding to an M_4_L_6_ complex stoichiometry were retained in *Z*·Co·L^m^; this photochemical switching is discussed in more detail in the next section. In the absence of crystallographic data, the geometry of *Z*·Co·L^m^ was optimised using GFN-xTB assuming a similar M_4_ cyclic structure as for *E*·Co·L^m^ with (i) all metal centres possessing a *mer* tris-chelate coordination geometry and (ii) having same optical configuration as one another (Δ, Fig. 17).

The structure adopts a similar conformation to *E*·Co·L^m^, displaying two inequivalent M_2_L_2_ helical units, connected at each end by two additional bridging ligands. This generated structure of an isolated molecule of *Z*·Co·L^m^ is less compact than the molecular structure observed in crystals of *E*·Co·L^m^, which we attribute to the greater solid-state constraints associated with crystal packing. In this calculated structure of *Z*·Co·L^m^, inter-ligand π-stacking was observed between relatively electron-rich phenyl rings of one ligand and coordinated pyrazolyl-pyridine units from other ligands which are relatively electron-deficient due to their coordination to +2 ions. In contrast to *E*·Co·L^m^, both helical units in the calculated structure of *Z*·Co·L^m^ displayed local *C*_2_ symmetry, which is removed by the orientation of the two helical units with respect to each other. This calculated M_4_ cyclic structure therefore possesses no symmetry elements, which would lead to 6 independent ligand environments displaying 144 different ^1^H-NMR signals in solution.

Samples of *Z*·Co·L^m^ could be prepared by two procedures: (i) reaction of Co(BF_4_)_2_ (7.0 mM) with a sample of *Z*-L^m^ (10.5 mM) in acetonitrile; or (ii) irradiation of pre-formed *E*·Co·L^m^ in acetonitrile with 340 nm light. Comparison of the observed ^1^H NMR spectra of the two samples (Fig. 16b and c, respectively) show that they are generally similar but contain major and minor components, whose relative intensities were dependent on the method by which the samples were prepared. The latter method [direct reaction of Co(ii) with photo-generated *Z*-L^m^, Fig. 16b] afforded a ^1^H NMR spectrum with a major component of >100 signals, consistent with the optimised structure generated by GFN-xTB (Fig. 17). In contrast, irradiation of *E*·Co·L^m^ in acetonitrile with 340 nm generated a more complex NMR spectrum containing additional minor signals (compare the features labelled * between Fig. 16b and c), implying formation of a less clean mixture of multiple species by photoswitching of pre-assembled *E*·Co·L^m^.

If cyclic M_4_L_6_ species can form with the ligands in either *E* (crystal structure, Fig. 13) or *Z* (calculated structure, Fig. 17) geometries, this implies that other intermediate M_4_L_6_ structures might exist containing different proportions of *Z*- and *E*-L^m^ ligands, *viz.* a series of M_4_(*E*-L^m^)_*n*_(*Z*-L^m^)_6−*n*_ species which can tolerate *Z*/*E* configurational changes to individual ligands without disrupting the overall complex formulation, and that is the focus of this section.

We investigated this possibility by combining Co(BF_4_)_2_ with mixtures of *Z* and *E* forms of L^m^ in different proportions. Due to the low solubility of free L^m^ in acetonitrile, 15% (*v*/*v*) chloroform was added to aid dissolution. Two titrations were performed, whereby the free ligand *Z*-L^m^ was gradually added to the complex mixture *E*·Co·L^m^, and *vice versa*, with free *E*-L^m^ being added to the complex *Z*·Co·L^m^ [prepared by addition of Co(BF_4_)_2_ (4 mM) to *Z*-L^m^ (6 mM)]. The first titration, therefore, had the starting composition Co(BF_4_)_2_ (4 mM) and *E-*L^m^ (6 mM), to which was added aliquots of *Z*-L^m^ (0 → 6 mM): the second titration, conversely, started with a mixture of Co(BF_4_)_2_ (4 mM) and *Z*-L^m^ (6 mM), to which was added aliquots of *E*-L^m^ (0 → 6 mM). This meant that by the end of each titration it would be possible for the solution to select whichever combination of ligand isomers gave the most stable cage if one particular cage was significantly favoured, or a mixture of species otherwise. Any ligand not needed for the minimum-energy complex assembly would be free in solution. Performing this titration in both directions but to the same compositional end point (4 Co^2+^ ions: 6 *Z*-L^m^: 6 *E*-L^m^) avoids any issues with one of the species being particularly kinetically inert and thereby giving a misleading impression of thermodynamic stability.

**Fig. 18:**
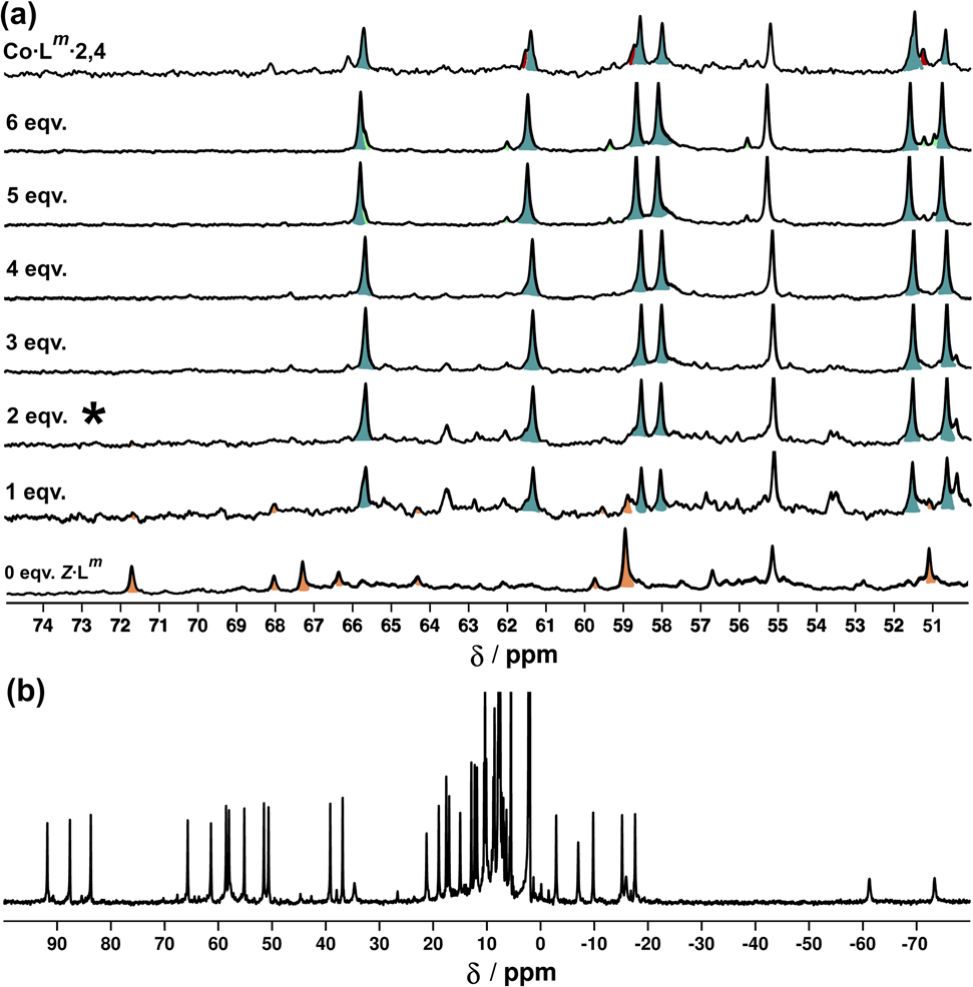
(a) ^1^H NMR spectra [300 MHz, MeCN-d_3_/CDCl_3_ (85 : 15 *v*/*v*), 298 K] recorded during addition of 0–6 equivalents (from bottom up) of *Z*-L^m^ to a solution of *E*·Co·L^m^. The spectrum labelled * (after addition of two equivalents of *Z*-L^m^) signals the point at which Co·L^m^·2,4 is the dominant species present with little change thereafter. (b) ^1^H-NMR spectrum (300 MHz, MeCN-d_3_/CDCl_3_ (85 : 15 *v/v*), 298 K) of Co·L^m^·2,4 prepared separately in the same solvent mixture – compare the 50–70 ppm region with the spectrum labelled * in Fig. 18a.

Fig. 18a shows the sequence of ^1^H NMR spectra measured during stepwise addition of portions of *Z*-L^m^ (1 mM) to *E*·Co·L^m^ in 85 : 15 (*v*/*v*) CD_3_CN/CDCl_3_. Addition of 1 equivalent of *Z*-L^m^ immediately resulted in appearance of a clear set of signals for a new complex: these new signals grew in intensity as the amount of *Z*-L^m^ increased to two equivalents (spectrum labelled * in Fig. 18a) with little change thereafter, and this species remains as the only metal complex structure following the addition of further equivalents of *Z*-L^m^ up to 6 mM. The pronounced appearance of this species after addition of two equivalents of *Z*-L^m^, and its retention thereafter, implies the formation of a complex with a 2 : 4 *Z* : *E* ligand ratio – which we denote Co·L^m^·2,4, with the numerals indicating the numbers of *Z* and *E* ligands respectively. Co·L^m^·2,4 therefore appears to be a particularly thermodynamically favoured mixed-ligand product. It could be prepared directly using the appropriate mixture of ligand isomers and Co(BF_4_)_2_ in the required proportions. The relatively simple and clean spectrum for Co·L^m^·2,4 suggests a single species, with ≈40 signals clearly resolved (Fig. 18b), consistent with the presence of two independent ligand environments having no internal symmetry which would give 48 distinct signals if all signals were clearly resolved with no overlap.

A similar result was obtained from the second titration in the opposite direction: on addition of portions of *E*-L^m^ to the complex *Z*·Co·L^m^, signals for Co·L^m^·2,4 dominated the spectra over a wide range of compositions (see ESI†). From this we can conclude that Co·L^m^·2,4, with the composition M_4_(*Z*-L^m^)_2_(*E*-L^m^)_4_, is the most stable of the ligand–isomer complex possibilities and we assume that it has the same cyclic structure as that observed for the crystal structure of *E*·Co·L^m^ but with two of the six ligands adopting the *Z* geometry: possibly the two ‘crosslinking’ ligands, as isomerisation of these would not disrupt the dinuclear helicate subunits.

### Metal complexes with L^m^: photoswitching

**Fig. 19:**
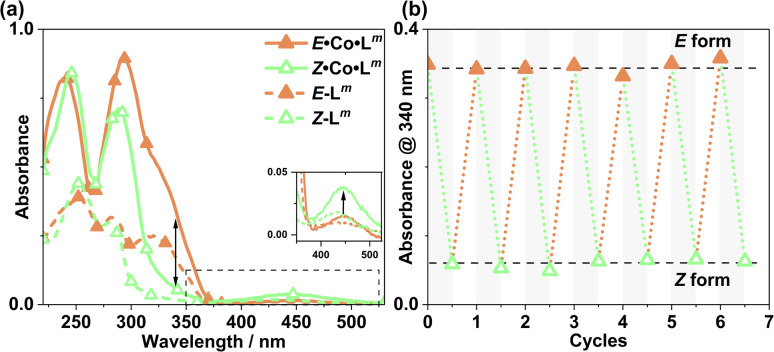
(a) UV/Vis spectra of *Z*·Co·L^m^ and *E*·Co·L^m^ in MeCN (20 μg ml^−1^), as well as *E*-L^p^ and *Z*-L^p^ (MeCN, 10 μM). (b) Switching stability experiment monitoring the absorbance of Co·L^m^ (20 μg ml^−1^) species at 340 nm, after alternating irradiation with 340 nm for 30 minutes and white light for 30 minutes.

UV/Vis spectroscopy (Fig. 19) was used to demonstrate repeated cycling between complexes containing *E*·Co·L^m^ (M_4_L_6_ complex possibly also containing other species such as M_2_L_3_ as part of a concentration-dependent equilibrium, see above) and the photoswitched *Z*·Co·L^m^ species whose mass spectrum indicates an M_4_L_6_ complex. As the UV/Vis spectra of the two forms have good separation of key absorption features, selective irradiation can be applied, and light-induced switching could be repeated over multiple cycles (Fig. 19b) with no appreciable photobleaching. We emphasise that this is not complete switching of the ‘molecule A converts to molecule B’ type, but switching of the composition of two metal complex ensembles in which the ligands are *E* to start with, but have the *Z* structure after photo-switching. The UV/Vis spectra (Co·L^m,^ 20 μg ml^−1^; and L^m^, 10 μM in acetonitrile) clearly confirm basically complete disappearance of the lower-energy absorption shoulder associated with the *E* form of L^m^ (Fig. 19a) following irradiation at 340 nm. Indeed, the similarity to the UV/Vis spectra associated with the *E/Z* switching of free L^m^ is striking (Fig. 2a), implying that the PSS under the forcing conditions of UV irradiation can be considered as approximately all-*Z*·Co·L^m^. After irradiation of the PSS with white light, the system completely reverted to the *E*·Co·L^m^ mixture of species, and the process could be repeated reversibly over several switching cycles.

**Fig. 20:**
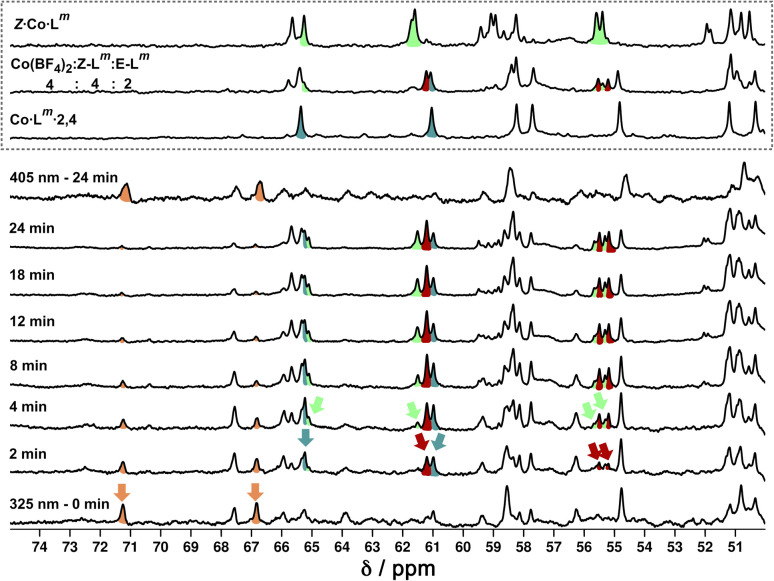
^1^H NMR (700 MHz, CD_3_CN, 298 K) spectral changes during illumination of *E*·Co·L^m^ (orange) with 325 nm light for 24 minutes, with subsequent illumination with 405 nm light for 24 minutes. Characteristic spectra of *Z*·Co·L^m^ (green) and Co·L^m^·2,4 (teal blue) prepared by combination of stoichiometric quantities of *Z* and *E*-L^m^ with Co(BF_4_)_2_, are shown for comparison. A third spectrum prepared by combination of *Z*-L^m^, *E*-L^m^ and Co(BF_4_)_2_ in a 4 : 2 : 4 ratio shows the presence of a fourth species (red) amongst signals associated with *Z*·Co·L^m^ and Co·L^m^·2,4. For discussion of signals labelled with coloured arrows, see main text.

We also used *in situ* illumination during NMR measurements to study the photo-induced interconversions between the set of complexes based on different ligand isomers, starting from *E*·Co·L^m^. Spectra were recorded every 40 seconds for 20 minutes under 325 nm irradiation for the *E* to *Z* switching, and then with 405 nm irradiation to reverse the ligand isomerisation (Fig. 20). As in the earlier photoswitching experiments, a narrow NMR spectral window (50–75 ppm) was selected in order to maximise the frequency resolution of the signals in this region and facilitate analysis.

Overlap of some ^1^H NMR signals due to the similarities between NMR spectra of isomeric structures made determination of integrals unreliable even after attempts at deconvolution, and also made evolution of spectra during the photoswitching difficult to follow with precision. However, it was still possible to see some diagnostic changes in the NMR spectra with time under irradiation. Starting with a solution of *E*·Co·L^m^, which has a messy spectrum as mentioned earlier, irradiation at 325 nm results in disappearance of some of the starting signals (denoted with orange arrows in Fig. 20, at around 67 and 71 ppm) over a period of ≈10 minutes. In parallel with this, new signals appeared at different rates. Signals labelled with a red arrow were not present in the starting spectrum of *E*·Co·L^m^ but appeared quickly during the irradiation, plateauing in intensity on a similar timescale to disappearance of *E*·Co·L^m^. The signals indicated with a teal blue arrow, in contrast, appeared to grow and then reduce in intensity, being most intense at around the 4–6 minutes mark before shrinking; and finally signals indicated with a green arrow were slowest to appear but then persisted.

This behaviour is consistent with photoswitched species containing different amounts of *Z*-L^m^ appearing in sequence, with the first such complex likely being Co·L^m^·2,4 – which is the complex that appears first and then disappears (teal blue signals) as it is further converted to species containing a higher proportion of *Z*-L^m^. Red signals seem to match numerous peaks visible in spectra containing a *Z* : *E* ligand ratio of 4 : 2. Literature precedents shows a tendency for cyclic bis-azobenzenes to favour *EE* and *ZZ* states, leading us tentatively to suggest concerted switching of pairs of azo-units *i.e.* two M_2_L_2_ units and the pair of cross-piece ligands connecting these helicates,^24,28^ as suggested earlier. Given the obvious complexity/high overlap between signals in these evolving spectra of mixtures we cannot assign confidently the nature of the new photoswitched species beyond that: we just note that (i) *E* to *Z* photoswitching occurs of ligands within the complexes, without changing the overall M_4_L_6_ stoichiometry; and (ii) the appearance and then disappearance of signals associated with Co·L^m^·2,4 is apparent as the complex population shifts towards species containing a higher proportion of the *Z* isomer under the forcing conditions (prolonged UV irradiation). After 25 minutes, a PSS was reached with no further significant changes in the ^1^H NMR spectra. Like the spectrum obtained following 340 nm irradiation, incomplete switching to *Z*·Co·L^m^ was observed, showing that in order to form *Z*·Co·L^m^ as the major species, L^m^ must be switched to the *Z* form by photoirradiation prior to assembly. As with the UV/Vis experiments, after 2 minutes of irradiation at 405 nm, the system completely reverted to the *E*·Co·L^m^ mixture of species.

Finally, we note (again) that thermal relaxation, after photoswitching, is slow. A solution subjected to photoswitching using 340 nm irradiation, as described above, had partially reverted after 2.5 weeks at ambient temperature with complete loss of the photoswitched species *Z*·Co·L^m^. After this time ^1^H NMR signals associated with the particularly stable species Co·L^m^·2,4 were present: but even after 5 weeks there was no *E*·Co·L^m^, with the presence of stable intermediate species such as Co·L^m^·2,4 hindering relaxation. After 10 months the complex had completely reverted to *E*·Co·L^m^.

## Conclusions

The bis-bidentate bridging ligands L^p^ and L^m^ contain two chelating pyrazolyl-pyridine coordination sites separated by photochromic azobenzene units based on *para* or *meta* substitution patterns, respectively. The light-induced *E* → *Z* conversion (under UV excitation) and subsequent reversion (under visible excitation) was clearly established by *in situ* NMR spectroscopic measurements which established compositions of the PSS as 94 : 6 *Z* : *E* and 95 : 5 *Z* : *E* for L^p^ and L^m^ respectively in MeCN.

The photochromic properties of the ligands could be exploited to effect photoswitching between different complex structures. The Co(ii) complex with *E*-L^p^ (denoted *E*·Co·L^p^) has NMR and MS data consistent with the dominant structure being a symmetric Co_2_L_3_ helicate with all three ligands equivalent and each ligand having twofold symmetry. Photoswitching (complete within detection limits of NMR spectroscopy) to *Z*·Co·L^p^ shows appearance by MS of a new M_4_L_6_ species whose more complex NMR spectrum is consistent with two independent ligand environments, characteristic of a *C*_3_-symmetric isomer of an M_4_L_6_ tetrahedral cage. The interconversion is fully reversible, and whilst we were unable to obtain X-ray quality crystals of these species, the combination of NMR and MS data points to fully reversible photo-switching between a Co_2_L_3_ helicate and a Co_4_L_6_ cage.

The Co(ii) complex with *E*-L^m^ (denoted *E*·Co·L^m^) has an ill-defined NMR spectrum indicative of a mixture of species, but it contains at least Co_2_L_3_ and Co_4_L_6_ species by ESI MS. It crystallises as a Co_4_L_6_ complex which is a pair of Co_2_L_2_ dinuclear double helicates crosslinked by two additional bridging ligands, such that the cyclic array of four Co(ii) ions has alternately one or two bridging ligands along each edge. NMR titrations, to examine the dominant speciation that occurred when Co(ii) ions could select from different proportions of *Z*-L^m^ and *E*-L^m^ in solution, indicated that the most stable assembly of composition M_4_(*Z*-L)_*n*_(*E*-L)_6−*n*_ has *n* = 2 (two *Z* ligands and four *E* ligands, denoted Co·L^m^·2,4), which persisted over a wide range of ligand isomer compositions. Photoswitching showed, by UV/Vis spectroscopy, conversion of the ligands to the *Z* form to give the species *Z*·Co·L^m^ amongst other *Z*-L^m^ complex species, which – by ESI MS – retain M_4_L_6_ compositions; *in situ* NMR experiments to monitor the photoswitching could not be fully interpreted but did show the appearance and then disappearance of a photoswitched initial product which is likely to be Co·L^m^·2,4*en route* to eventual formation of *Z*·Co·L^m^. Thermal relaxation of *Z*·Co·L^m^ was slow, with Co·L^m^·2,4 being the dominant species after several weeks: complete reversion to *E*·Co·L^m^ was complete by 10 months.

Overall the pair of ligands L^p^ and L^m^ both display photoswitching behaviour which translates into major structural changes in their metal complex assemblies, with unusual examples of (i) interconversion between Co_2_L_3_ (helicate) and Co_4_L_6_ (cage) assemblies with L^p^; and interconversion between a series of Co_4_L_6_ assemblies Co_4_(*Z*-L^m^)_*n*_(*E*-L^m^)_6−*n*_ with L^m^.

## Experimental

All experimental information (synthesis, characterisation data, X-ray crystallography, photoswitching instrumentation and methodologies, calculations of model structures and software used) are in the ESI.†

## Data availability

The datasets supporting this article have been uploaded as part of the ESI.†

## Author contributions

M. B. T.: synthesis, characterisation and UV/Vis based photoswitching studies. L. P. L.: NMR-based photoswitching studies and calculations of molecular models. A. B. S.: X-ray crystallography. L. K. S. v. K and M. D. W.: project conception and supervision.

## Conflicts of interest

There are no conflicts to declare.

## Supplementary Material

SC-015-D4SC01575D-s001

SC-015-D4SC01575D-s002
