# Supplementary material for: Photoswitching of Co(ii)-based coordination cages containing azobenzene backbones
Source: Chem Sci. 2024 May 3;15(22):8488–99. doi: 10.1039/d4sc01575d (PMC11151815; doi:10.1039/d4sc01575d)
Supplement: SC-015-D4SC01575D-s001 [file SC-015-D4SC01575D-s001.pdf]

Electronic Supplementary Material (ESI) for Chemical Science.
This journal is © The Royal Society of Chemistry 2024

Supporting Information For:
Photoswitching of Co(II)-based coordination cages containing azobenzene backbones

Max B. Tipping,[a] Lidón Pruñonosa Lara,[b] Atena B. Solea,[a] Larissa K. S. von Krbek*[b] and Michael D. Ward*[a]

a Department of Chemistry, University of Warwick, Coventry CV4 7AL, UK
b Kekulé-Institut für Organische Chemie und Biochemie, Rheinische Friedrich-Wilhelms-Universität Bonn, Gerhard-Domagk-Str. 1, 53121 Bonn, Germany

Corresponding Authors Email: m.d.ward@warwick.ac.uk, larissa.vonkrbek@uni-bonn.de

Contents

Materials 4
Methods 4
NMR Spectroscopy 4
Mass Spectrometry 4
UV/Vis Spectroscopy 4
IR Spectroscopy 4
Light Sources 4
Preparations 5
L^m: 5
E•Co•L^p 5
E•Zn•L^p 5
Z•Co•L^p 5
Z•Zn•L^p: 5
E•Co•L^m: 6
E•Zn•L^m: 6
Z•Co•L^m 6
Selected NMR Spectra 7
L^m 7
E•Zn•L^p 8
Z•Zn•L^p 9
E•Co•L^m 11
E•Zn•L^m 11
Z•Zn•L^m 13
Mass Spectra 14
E•Co•L^p 14
E•Zn•L^p 14
Z•Co•L^p 15
Z•Zn•L^p 15
E•Co•L^m 16
E•Zn•L^m 17
Z•Co•L^m 18
Co•L^m•2,4 19
UV/Vis Spectroscopy 20
L^p 20
L^m 20
Co•L^p 21
Co•L^m 21
Variable Temperature NMR 22
Thermal Relaxation NMR Experiments 24
Ligands 24
Z-L^p 65 °C 24
Z-L^m 25 °C 25

Z-L^m 65 °C 26
Ligand Half-Life 27
Z-L^p 27
Z-L^m 27
Cages 28
Z•Co•L^p 28
Z•Co•L^m 29
Titrations 30
Mixed Ligand Cage Formation 30
Addition of Z-L^m to E•Co•L^m 32
Addition of E-L^m to Z•Co•L^m 33
***In situ* NMR Spectra** 34
E-L^p - 365 nm 35
Z-L^p - 450 nm 35
E•Co•L^p – 365 nm 36
Z•Co•L^p – 405 nm 36
E-L^m – 325 nm 37
Z-L^m – 450 nm 37
E•Co•L^m – 325 nm 38
Z•Co•L^m – 405 nm 38
X-ray crystallography 39
Simulations 41
Co•L^p 41
Co•L^m 43
References 45

Materials

Starting materials were purchased from the following commercial sources: Fluorochem, Sigma-Aldrich, Fisher Scientific and Alfa Aesar and used as supplied. The following materials were prepared using literature procedures: 3,3'-bis(bromomethyl)-azobenzene and 4,4'-bis(bromomethyl)-azobenzene;[1] 3-(2-pyridyl)-1H-pyrazole;[2] $\mathbf{L}^{\boldsymbol{p}}$.[3]

Methods

NMR Spectroscopy - ^{1}H NMR and ^{13}C{^{1}H} NMR spectra were recorded at 300, 400, 500 or 700 MHz (^{1}H) and 125 MHz (^{13}C) respectively on Bruker Avance (300 MHz), Bruker Avance III HD (400 MHz), Bruker Avance III HD (500 MHz), Bruke Avance I (500 MHz) or Bruker Avance III HD Ascend (700 MHz) spectrometers. ^{19}F{^{1}H} NMR were also recorded on a Bruker Avance III HD (400 MHz). All NMR spectra were measured at 25°C in the indicated deuterated solvents unless stated otherwise. Proton and carbon chemical shifts (δ) are reported in ppm and coupling constants (*J*) are reported in Hertz (Hz). The resonance multiplicity in the ^{1}H NMR spectra are described as "s" (singlet), "d" (doublet), "t" (triplet), "q" (quartet), ''dd'' (doublet of doublets), ''ddd'' (doublet of doublet of doublets) and "m" (multiplet) and broad resonances are indicated by "br". 2D homonuclear correlation ^{1}H-^{1}H COSY and 2D heteronuclear correlation ^{1}H-^{13}C HETCOR experiments (HSQC, HMBC) were used to confirm NMR peak assignments.

Mass Spectrometry - Low resolution ESI mass spectrometry was performed using an Agilent 6130B ESI-MS; high resolution ESI mass spectra were acquired on a Bruker Compact ESI-Q-TOF.

UV/Vis Spectroscopy - recorded on an Implen C40 Nanophotometer and/or a Clariostar Plus (BMG Labtech) plate reader.

IR Spectroscopy - recorded on a Bruker Alpha FTIR Spectrometer.

Light Sources - Illumination experiments were carried out using Prizmatrix fiber collimated LEDs and a Thorlabs single colour UV LED (340 nm light source).

Table S1. Light output of LEDs used.

**λ / nm**	**310**	**325**	**340***	**365**	**390**	**405**	**450**	**500**	**550**
Output power (1 m optical fiber) / mW	1	8.6	1.7	170	220	260	440	165	230

* 340 nm is not a fibre collimated LED and, hence, output power is given directly at the light source.

340 nm LEDs (only) were unavailable for *in situ* illumination NMR experiments, but were used for endpoint experiments and synthesis.

Preparations

***L^m^*:** Aqueous KOH (5.5 M, 51 cm^3, 280.5 mmol) was added to a stirred solution of 3-(2-pyridyl)-1H-pyrazole (4.20 g, 28.9 mmol) in THF (250 cm^3). Once effervescence had ceased, 3,3′-bis(bromomethyl)-azobenzene (5.00 g, 13.7 mmol) was added along with a catalytic quantity of tetrabutylammonium iodide and the solution heated to 74 °C for 20 h. Once cooled to RT, the crude mixture was diluted with H_2O and then reduced in volume by 50% under reduced pressure. The crude product was extracted using DCM (3 x 50 cm^3), dried over $MgSO_4$, filtered and solvent removed by rotary evaporation. Column chromatography on silica ($THF/CHCl_3$, 1:9 v/v) yielded $\mathbf{L^m}$ as an orange powder (4.1 g, 61%). High-resolution ESI-MS: m/z 497.2192; calcd. for $C_{30}H_{25}N_8^+$ $(M + H)^+$, 497.2197. IR: ν_{max}/cm^{-1} 3121w, 3048w, 2918m, 2850w, 1743w, 1707w, 1591m, 1564m, 1491m, 1459m, 1226m, 1143m, 1048m, 993m, 755s, 695s, 634m, 621m, 539m. ^{1}H-NMR (500 MHz, $CDCl_3$, RT): δ 8.69 (2H, d, J 4.3 Hz, pyridyl H^6); 7.96 (2H, d, J 7.9 Hz, pyridyl H^3); 7.84 (2H, d, J 8.0 Hz, phenyl H^6), 7.81 (2H, s, phenyl H^2), 7.71 (2H, td, J 7.9, 1.8 Hz, pyridyl H^4), 7.49-7.47 (4H, m, pyrazole H^5 and phenyl H^5), 7.35 (2H, d, J 7.9 Hz, phenyl H^4), 7.19 (2H, dd, J 6.9, 5.4 Hz, pyridyl H^5), 6.96 (2H, d, J 2.1 Hz, pyrazole H^4), 5.48 (4H, s, CH_2). ^{13}C-NMR (125 MHz, $CDCl_3$, RT): δ152.7 (Phenyl C^1), 152.0 Pyridyl C^2), 151.7 Pyrazole C^3), 149.2 (Pyridyl C^6), 137.6 (Phenyl C_3), 136.7 (Pyridyl C^4), 131.0 (Pyrazole C^5), 130.2 (Phenyl C^4), 129.6 (Phenyl C^5), 122.7 (Phenyl C^6), 122.4 (Pyridyl C^5), 122.0 (Phenyl C^2), 120.2 (Pyridyl C^3), 105.1 (Pyrazole C^4), 55.9 (CH_2).

E•Co•L^p^: $\mathbf{L^p}$ (50 mg, 101 μmol) and $Co(BF_4)_2.6H_2O$ (23 mg, 68 μmol) were combined in methanol (10 cm^3) and heated to 60 °C overnight. The suspension was centrifuged and the solid washed with methanol, CH_2Cl_2, and diethyl ether. The resulting compound was dried in air. Yield: 60 mg (91%). High-resolution ESI-MS: m/z 890.2472 (calcd. for $\{Co_2(\mathbf{L^p})_3(BF_4)_2\}^{2+}$, 890.2554); 564.4983 (calcd. for $\{Co_2(\mathbf{L^p})_3(BF_4)\}^{3+}$, 564.5021); 401.8743 (calcd. for $\{Co_2(\mathbf{L^p})_3\}^{4+}$, 401.8761). ^{1}H NMR spectra shown in **Figure 6**.

E•Zn•L^p^ was prepared in the same way as *E*•**Co•L^p^**, but using $Zn(BF_4)_2.xH_2O$ (21 mg, 68 μmol, assumed to be the hexahydrate) in place of $Co(BF_4)_2.6H_2O$. Yield: 58 mg (88%). High-resolution ESI-MS: m/z 897.2521 (calcd. for $\{Zn_2(\mathbf{L^p})_3(BF_4)_2\}^{2+}$, 897.2501); 569.1662 (calcd. for $\{Zn_2(\mathbf{L^p})_3(BF_4)\}^{3+}$, 569.1653); 405.1236 (calcd. for $\{Zn_2(\mathbf{L^p})_3\}^{4+}$, 405.1229). ^{1}H NMR spectra shown in **Figure S4**.

Z•Co•L^p^: A sample of *E*•**Co•L^p^** (10 mg) was dissolved in MeCN-d_3 (1 cm^3), transferred to a quartz cuvette and irradiated with a 1.7 mW 340 nm LED overnight. High-resolution ESI-MS: m/z 1216.3280 (calcd. for $\{Co_4(\mathbf{L^p})_6(BF_4)_5\}^{3+}$, 1216.3428); 890.4975 (calcd. for $\{Co_4(\mathbf{L^p})_6(BF_4)_4\}^{4+}$, 890.5060); 694.9983 (calcd. for $\{Co_4(\mathbf{L^p})_6(BF_4)_3\}^{5+}$, 695.0039); 564.6636 (calcd. for $\{Co_4(\mathbf{L^p})_6(BF_4)_2\}^{6+}$, 564.6692). ^{1}H NMR spectrum shown in Figure 6 and spectra recorded during the interconversion are shown in **Figure S45**.

***Z•Zn•L^p^*:** A sample of *E*•**Zn•L^p^** (10 mg) was dissolved in MeCN-d_3 (1 cm^3), transferred to a quartz cuvette and irradiated with a 1.7 mW 340 nm LED overnight. High-resolution ESI-

MS: m/z 1224.6689 (calcd. for $\{Zn_4(\mathbf{L^{p}})_6(BF_4)_5\}^{3+}$, 1224.6692). For ^1H NMR spectrum, see **Figure S6**.

E•Co•L^m^: $\mathbf{L^m}$ (50 mg, 101 μmol) and $Co(BF_4)_2.6H_2O$ (23 mg, 68 μmol) were combined in methanol and stirred at 60 °C overnight. After cooling to room temperature, the solvent was removed via rotary evaporation. The resulting solid was washed with ethyl acetate and diethyl ether. Yield: 60 mg, (91%). For ^1H NMR spectrum, see main text. High-resolution ESI-MS: m/z 1216.2931 (calcd. for $\{Co_4(\mathbf{L^{m}})_6(BF_4)_5\}^{3+}$, 1216.3428); 890.4694 (calcd. for $\{Co_4(\mathbf{L^{m}})_6(BF_4)_4\}^{4+}$, 890.5060); 694.9746 (calcd. for $\{Co_4(\mathbf{L^{p}})_6(BF_4)_3\}^{5+}$, 695.0039). For ^1H NMR spectrum, see **Figure 16**.

E•Zn•L^m^: this was prepared exactly as for *E*•**Co•L^m^** but using $Zn(BF_4)_2$ (21 mg, 68 μmol, assumed to be the hexahydrate) in place of $Co(BF_4)_2.6H_2O$. Yield: 59 mg, (89%). High-resolution ESI-MS: *m/z* 1225.0099 (calcd. for $\{Zn_4(\mathbf{L^{m}})_6(BF_4)_5\}^{3+}$, 1225.0020). For ^1H NMR spectrum, see **Figure S10**.

Z•Co•L^m^: $\mathbf{L^m}$ (6.0 mg, 12 μmol) was dissolved in MeCN (1 cm^3) and irradiated with 340 nm overnight, to produce *Z*-$\mathbf{L^m}$**.** $Co(BF_4)_2.6H_2O$ (19.2 mg, 56 μmol) was dissolved in acetonitrile (1 cm^3). *Z*-$\mathbf{L^m}$ solution (0.7 cm^3) and $Co(BF_4)_2$ solution (0.1 cm^3) were combined and vortexed. High resolution ESI-MS: m/z 1867.9415 (calcd. for $\{Co_4(\mathbf{L^{m}})_6(BF_4)_6\}^{2+}$, 1868.0164); 1216.2930 (calcd. for $\{Co_4(\mathbf{L^{m}})_6(BF_4)_5\}^{3+}$, 1216.3428); 890.4697 (calcd. for $\{Co_4(\mathbf{L^{m}})_6(BF_4)_4\}^{4+}$, 890.5060); 694.9747 (calcd. for $\{Co_4(\mathbf{L^{p}})_6(BF_4)_3\}^{5+}$, 695.0039). ^1H NMR spectrum shown in Figure 16 and spectra recorded during the interconversion are shown in **Figure 16** and **Figure S48**.

Selected NMR Spectra

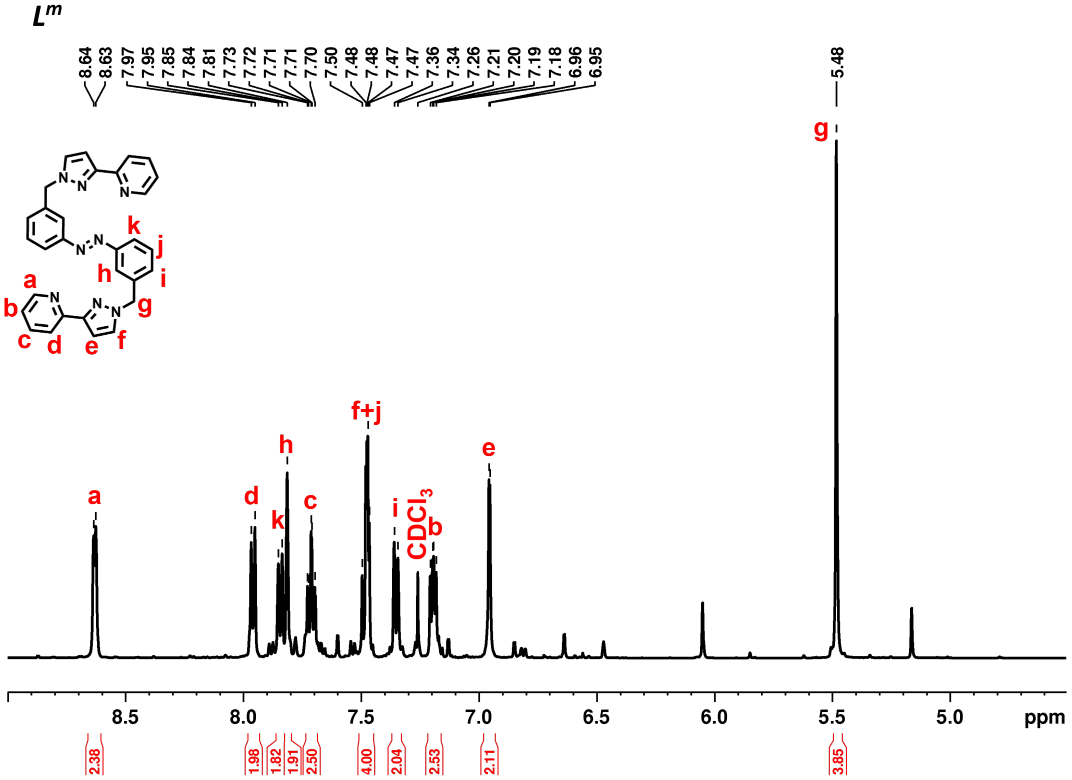

Figure S1. ^{1}H NMR spectrum (500 MHz, $CDCl_3$, 298 K) of *E*-**L*^m^***.

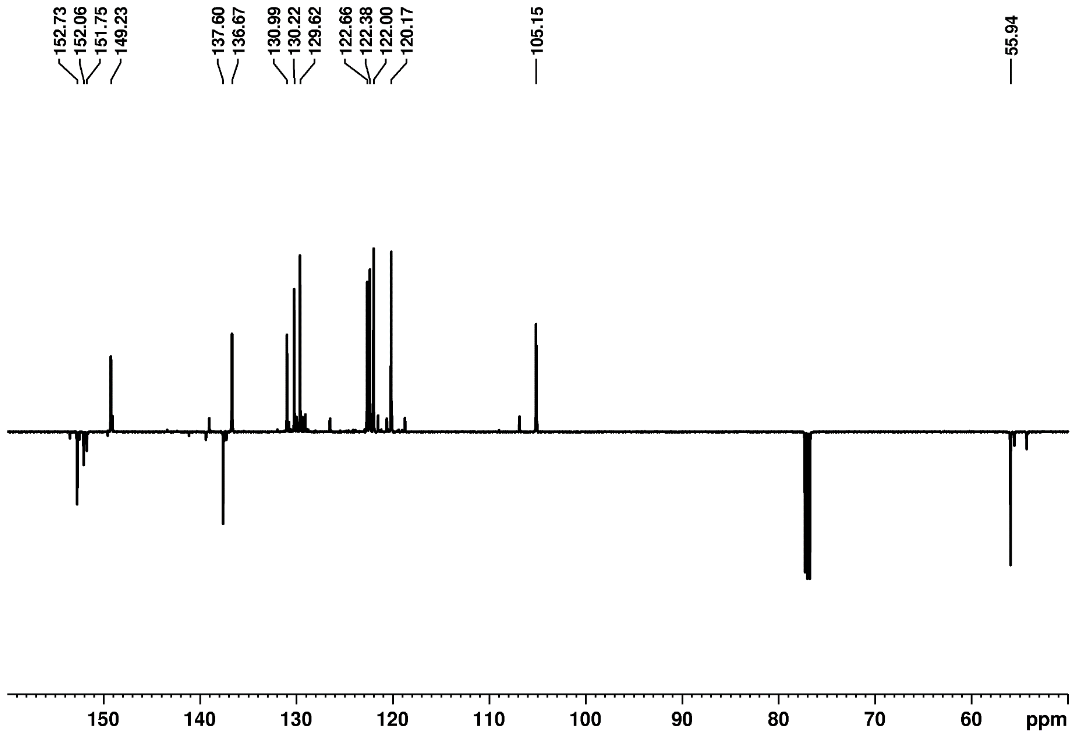

Figure S2. $^{13}C\{^{1}H\}$ APT NMR spectrum (125 MHz, $CDCl_3$, 298 K) of *E*-**L*^m^***.

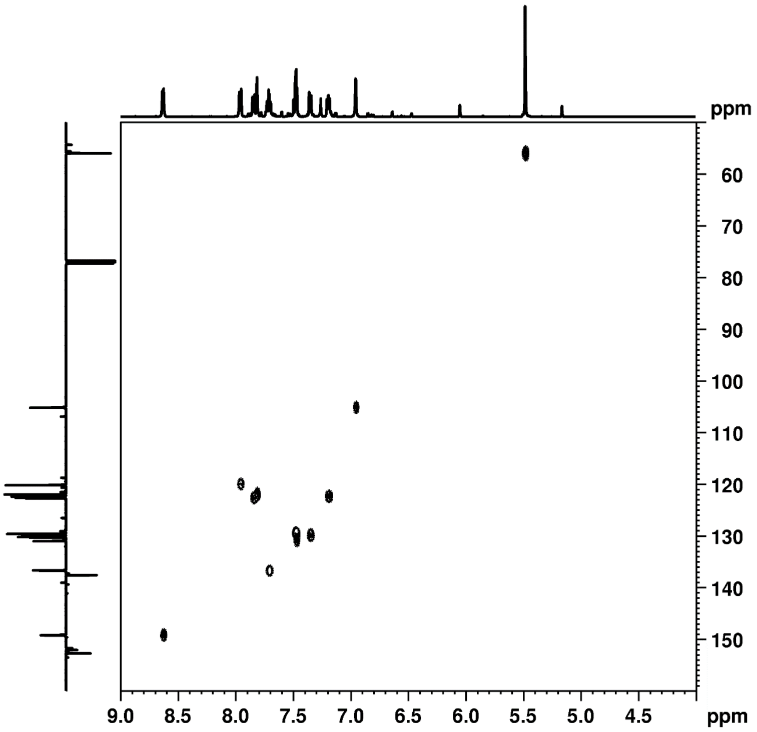

Figure S3. 2D ^{1}H-^{13}C HSQC NMR spectrum ($CDCl_3$, 298 K) of *E*-**L**^{m}

E•Zn•L^{p}

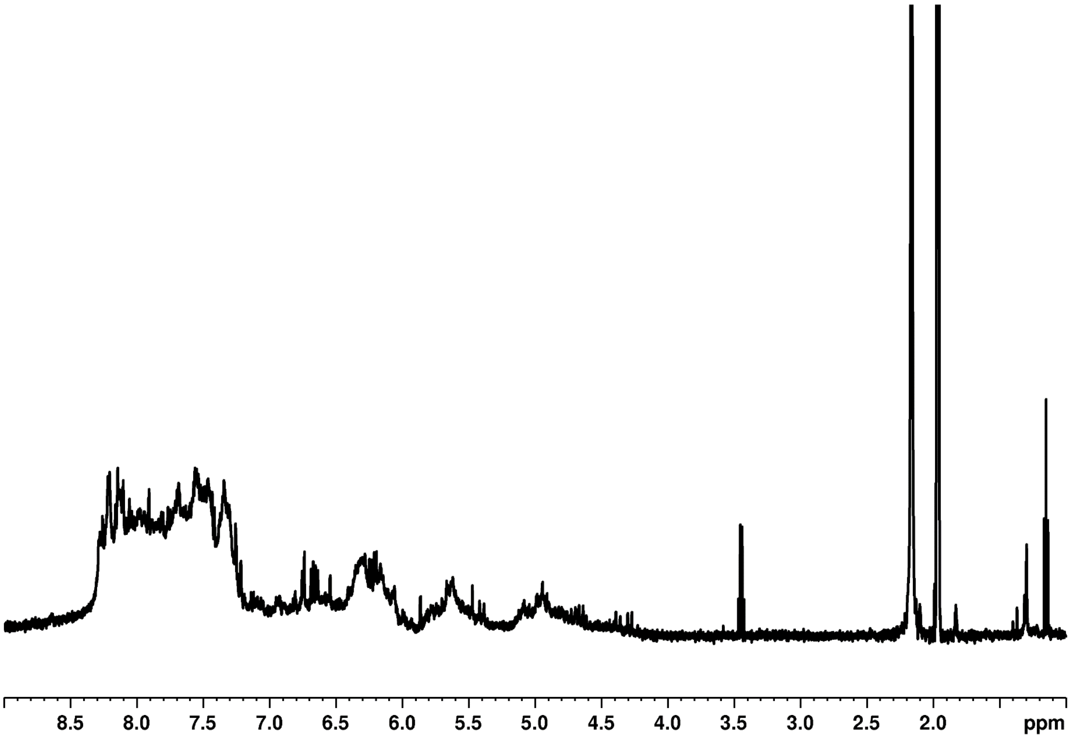

Figure S4. ^{1}H NMR spectrum (500 MHz, MeCN-d_3, 298 K) of *E*•**Zn**•**L**^{p}

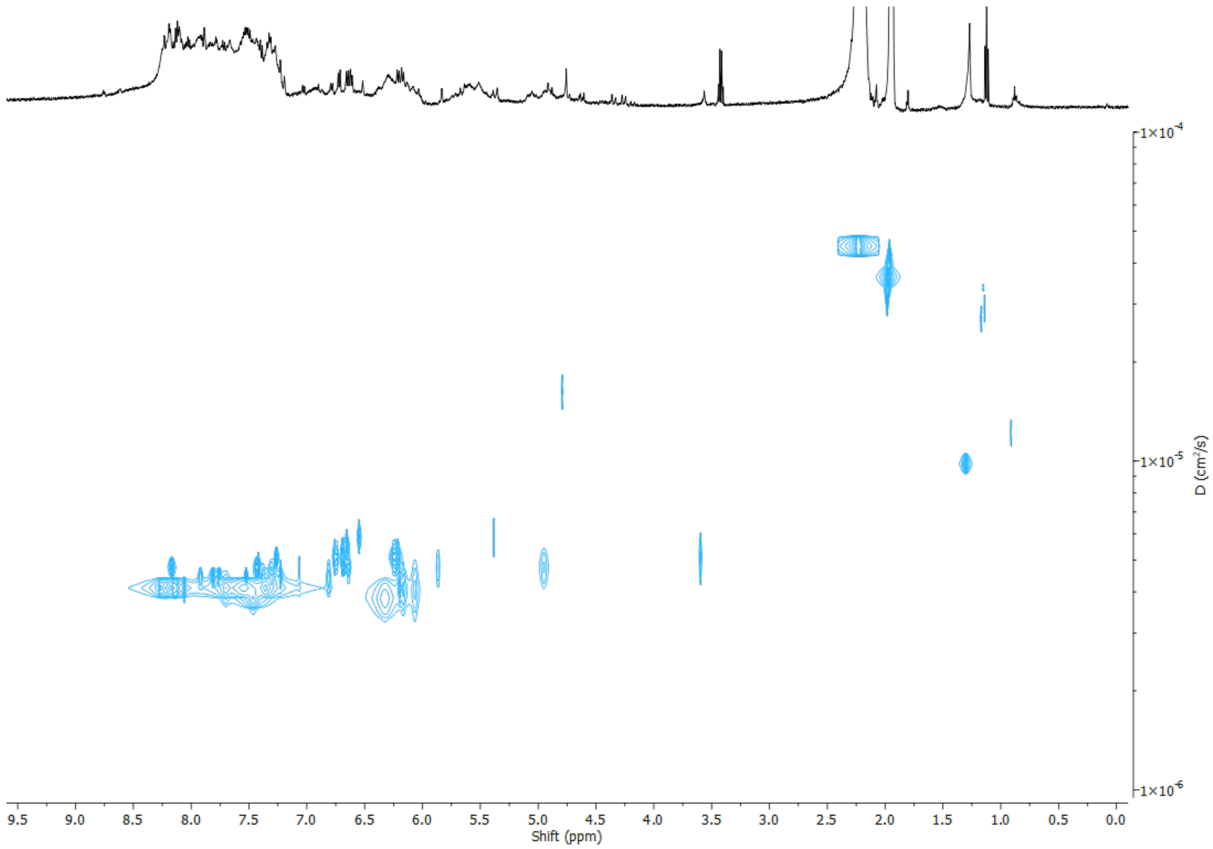

Figure S5. 2D-DOSY NMR spectrum (500 MHz, MeCN-d$_3$, 298 K) of *E*•**Zn•L***^{p}*

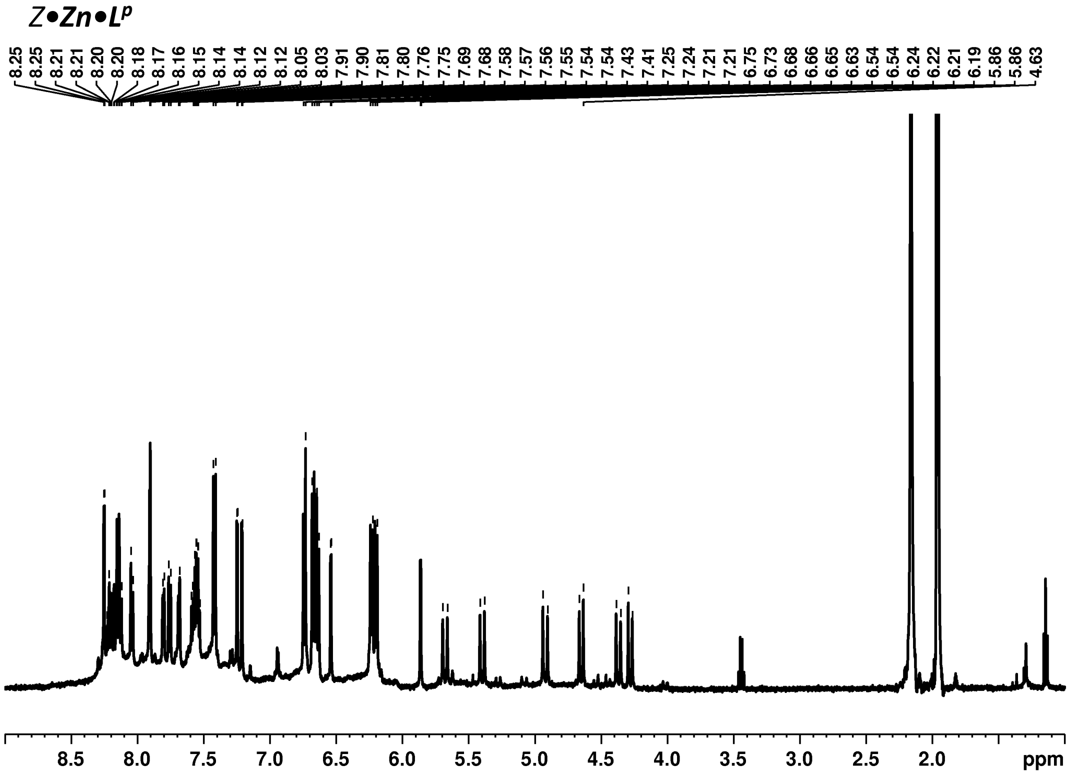

Figure S6. ^{1}H NMR spectrum (500 MHz, MeCN-d$_3$, 298 K) of *Z*•**Zn•L***^{p}*

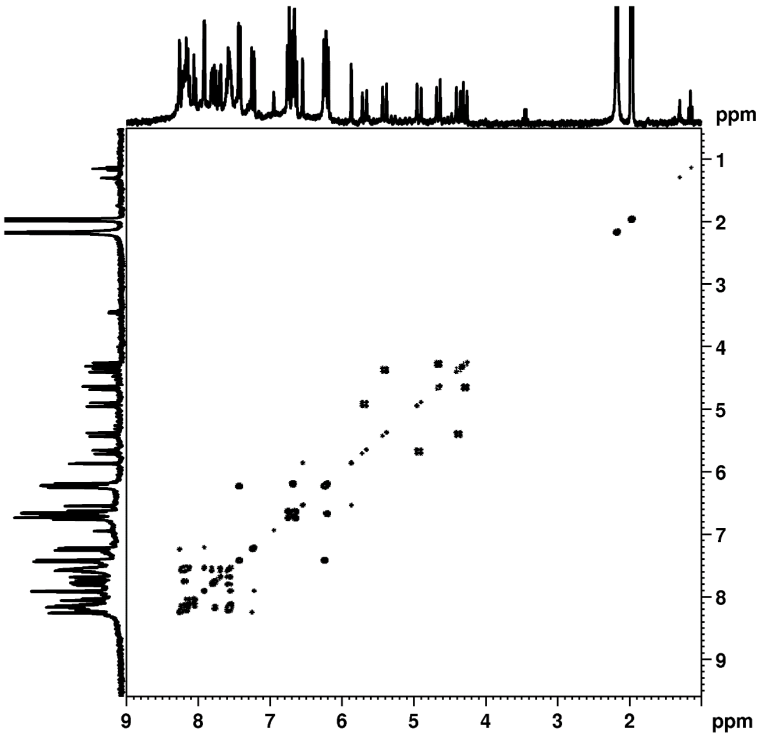

Figure S7. 1H-1H COSY spectrum (300 MHz, MeCN-d$_3$, 298 K) of *Z*•**Zn•L**^{p}.

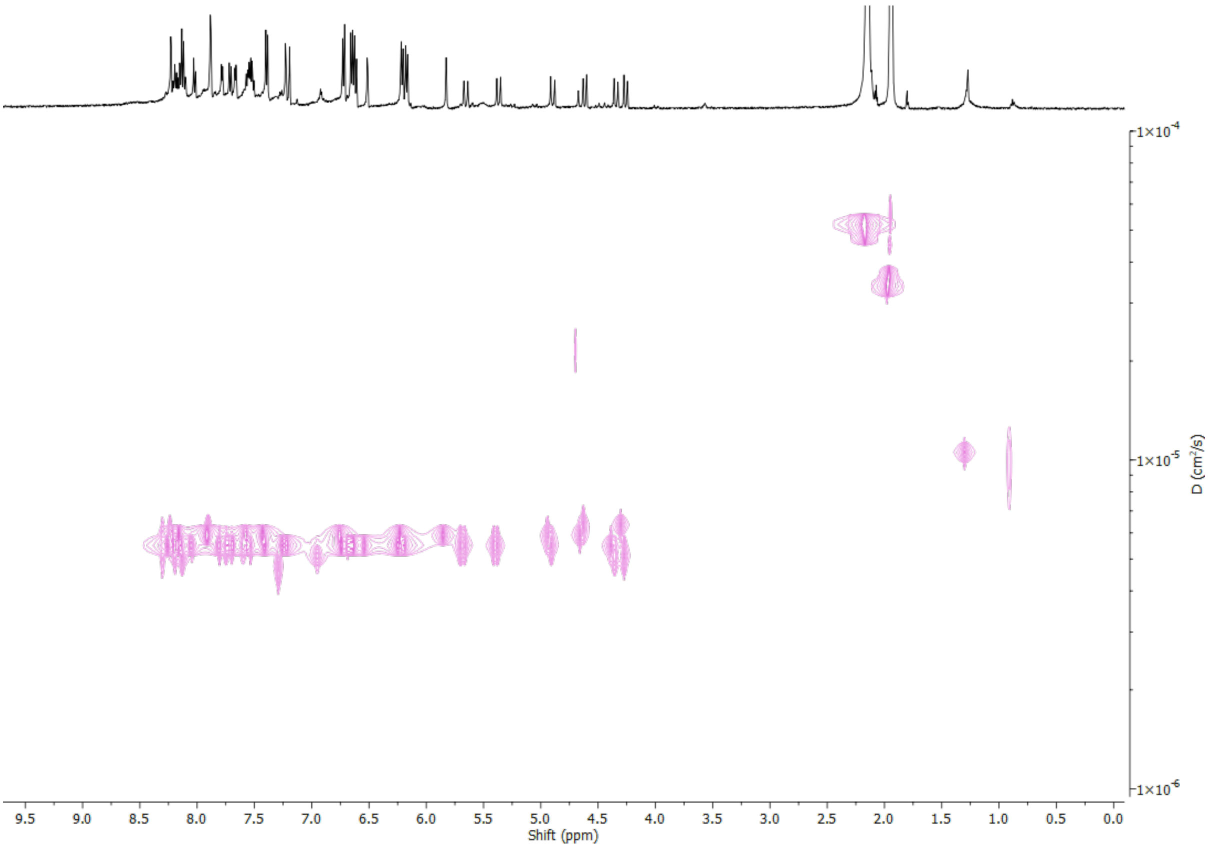

Figure S8. 2D-DOSY NMR spectrum (500 MHz, MeCN-d$_3$, 298 K) of *Z*•**Zn•L**^{p}

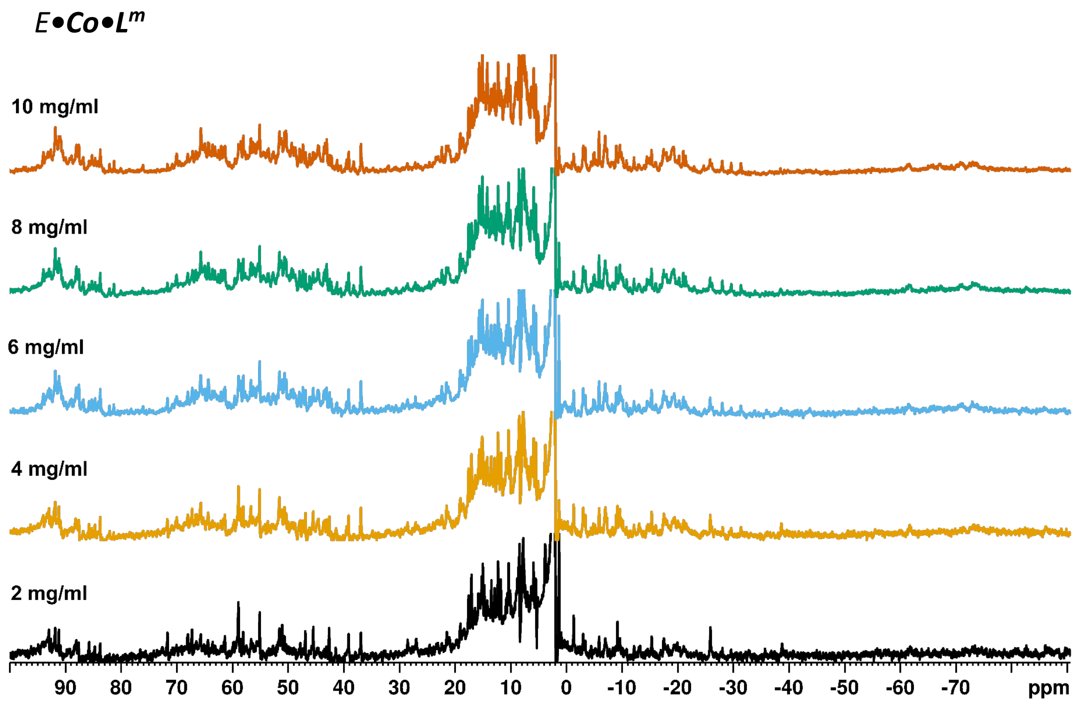

Figure S9. ^{1}H NMR spectrum (300 MHz, CD_3CN-d_3, 298 K) of *E*•**Co**•**L***^{m}* at various concentrations.

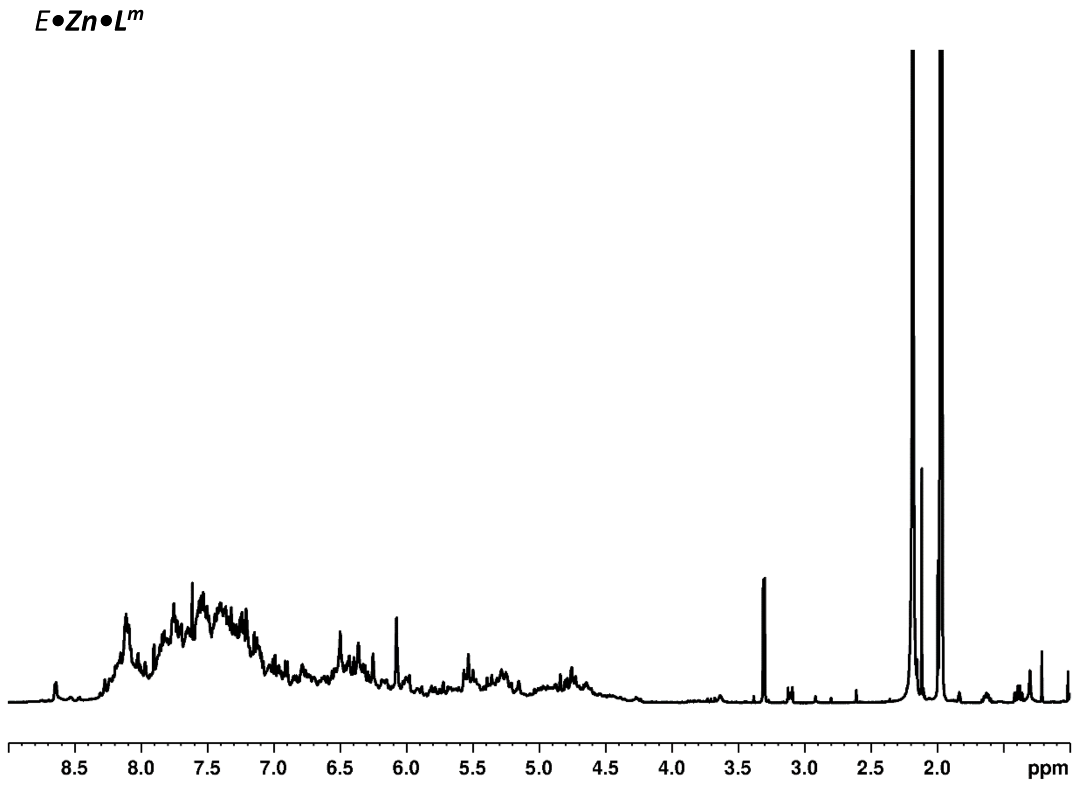

Figure S10. ^{1}H NMR spectrum (500 MHz, MeCN-d_3, 298 K) of *E*•**Zn**•**L***^{m}*

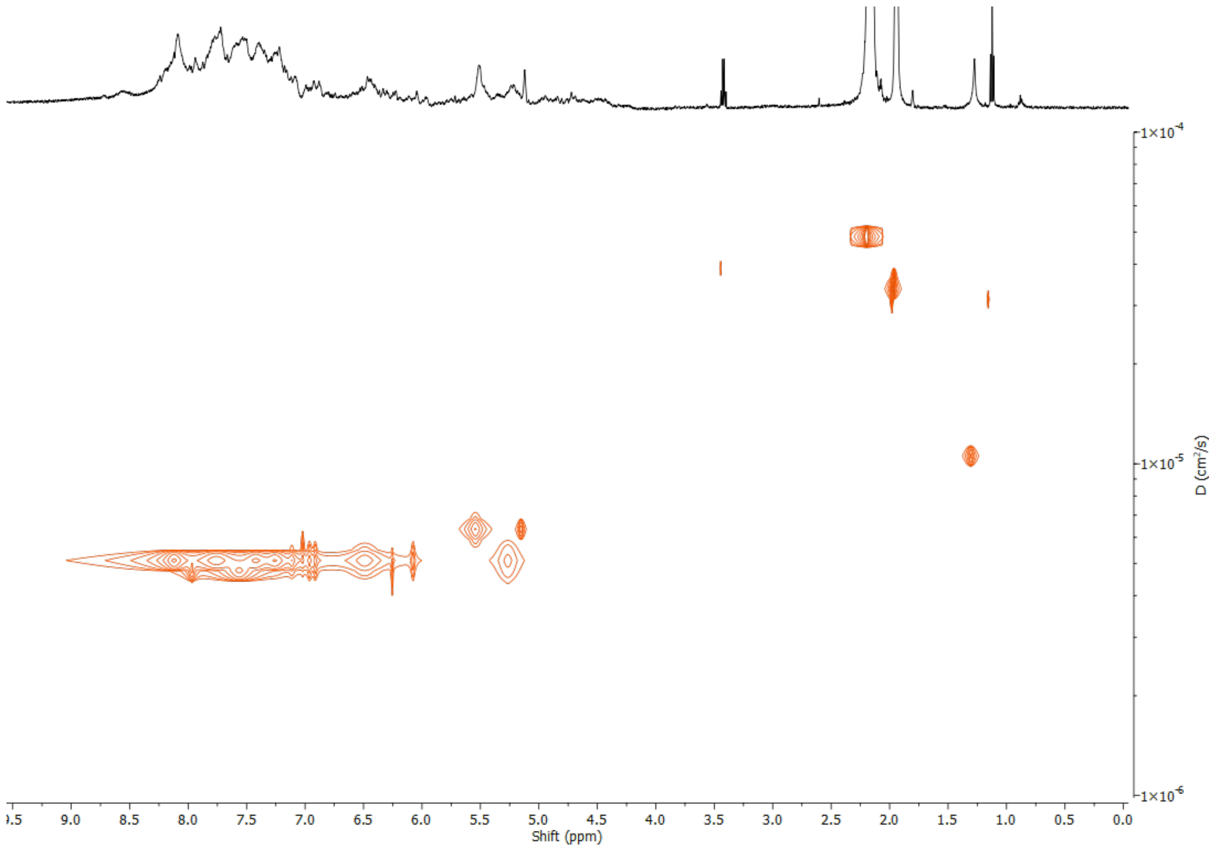

Figure S11. 2D-DOSY NMR spectrum (500 MHz, MeCN-d_3, 298 K) of ***E*•Zn•L*ᵐ***

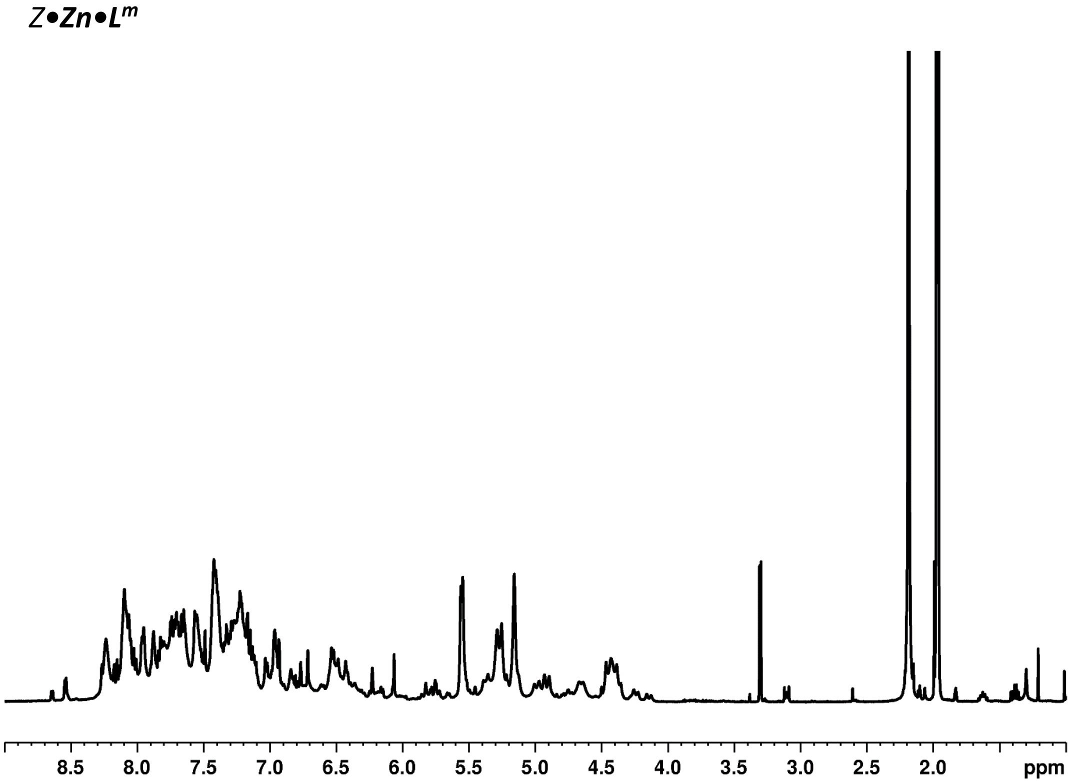

Figure S12. ^{1}H NMR spectrum (500 MHz, MeCN-d$_3$, 298 K) of *Z*•**Zn**•**L***^{m}*

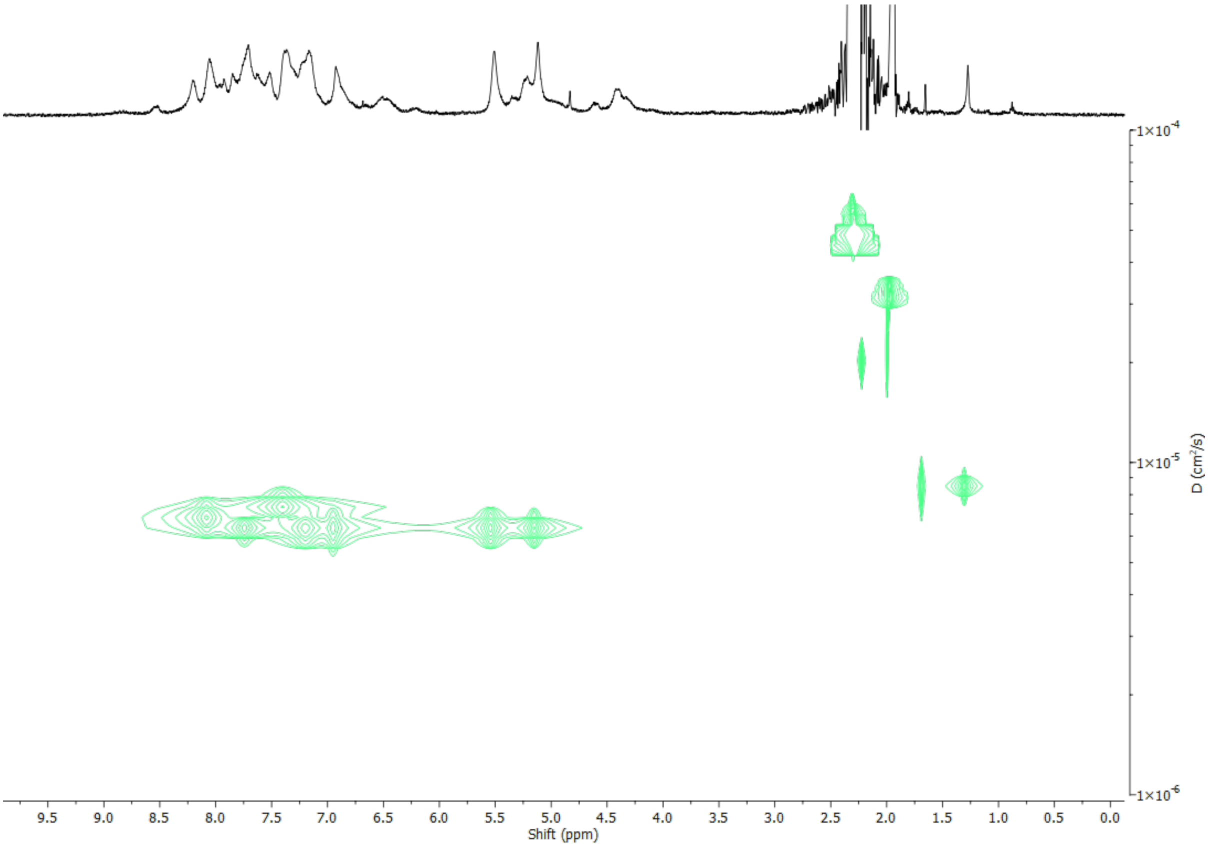

Figure S13. 2D-DOSY NMR spectrum (500 MHz, MeCN-d$_3$, 298 K) of *Z*•**Zn**•**L***^{m}*

Mass Spectra

E•*Co*•*L^p*

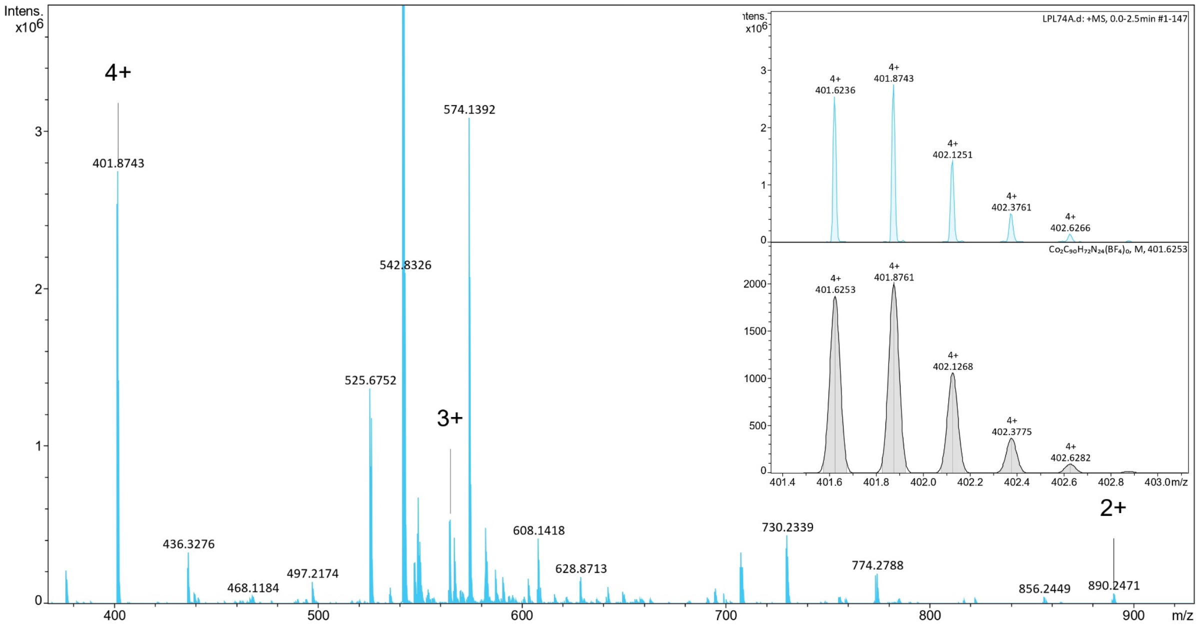

Figure S14. HR-ESI-MS of $[Co_2(E\text{-}\mathbf{L^p})_3(BF_4)_4]$ [*E*•**Co**•**L^p**] in CH_3CN with signals corresponding to $[Co_2(E\text{-}\mathbf{L^p})_3(BF_4)_n]^{(4-n)+}$ (n = 0, 1, 2). Expansion of $[Co_2(E\text{-}\mathbf{L^p})_3]^{4+}$ is inset.

E•*Zn*•*L^p*

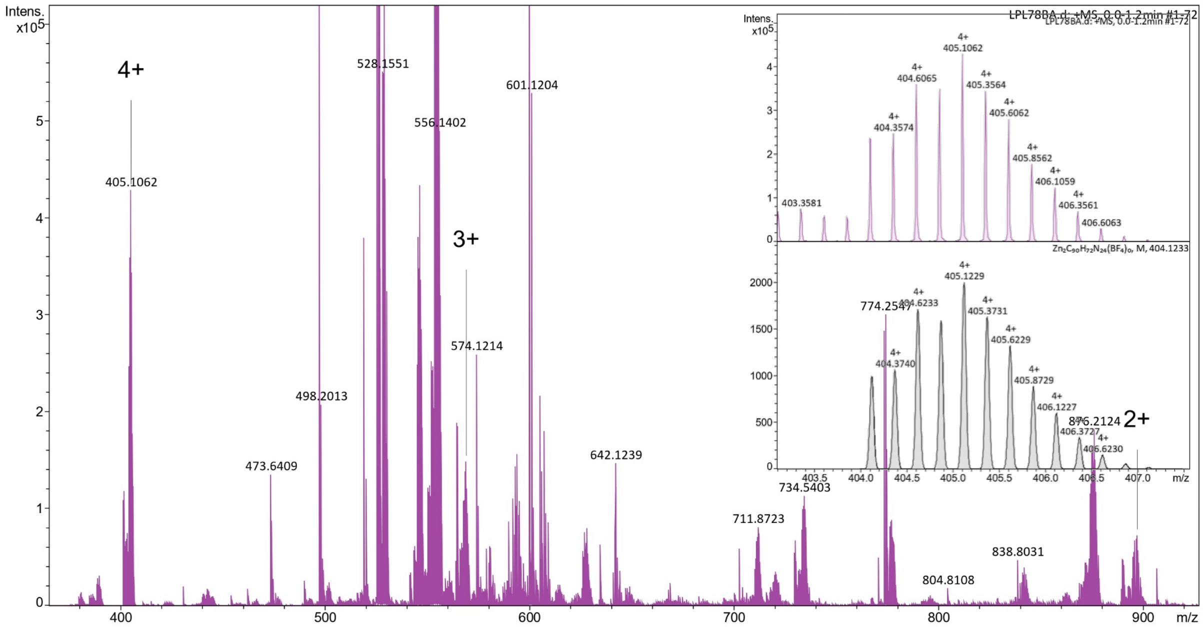

Figure S15. HR-ESI-MS of $[Zn_2(E\text{-}\mathbf{L^p})_3(BF_4)_4]$ [*E*•**Zn**•**L^p**] in CH_3CN with signals corresponding to $[Zn_2(E\text{-}\mathbf{L^p})_3(BF_4)_n]^{(4-n)+}$ (n = 0, 1, 2). Expansion of $[Zn_2(E\text{-}\mathbf{L^p})_3]^{4+}$ is inset.

Z•***Co***•***L***^{p}

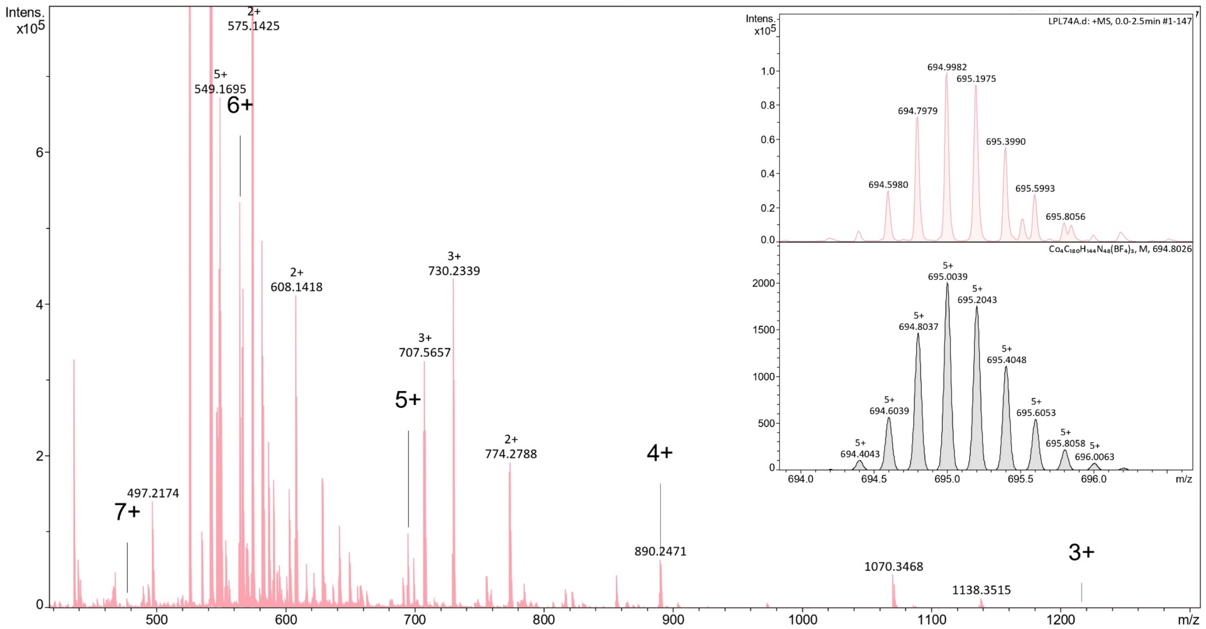

Figure S16. HR-ESI-MS of [Co_4(*Z*-**L**^{p})$_6$(BF_4)$_8$] [*Z*•**Co**•**L**^{p}] in CH_3CN with signals corresponding to [Co_4(*Z*-**L**^{p})$_6$(BF_4)$_n$]$^{(8-n)+}$ (n = 1, 2, 3, 4, 5). Expansion of [Co_4(*Z*-**L**^{p})$_6$(BF_4)$_3$]$^{5+}$ is inset.

Z•***Zn***•***L***^{p}

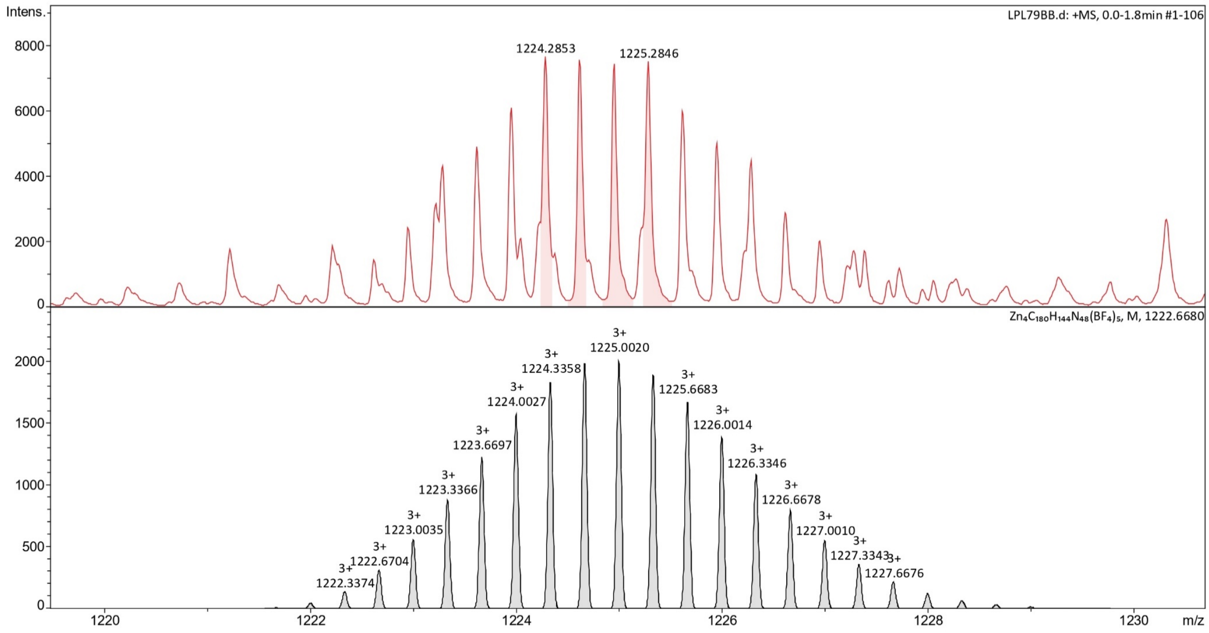

Figure S17. HR-ESI-MS of [Zn_4(*Z*-**L**^{p})$_6$(BF_4)$_8$] [*Z*•**Zn**•**L**^{p}] showing expansion of [Zn_4(*Z*-**L**^{p})$_6$(BF_4)$_5$]$^{3+}$.

E•Co•L^m

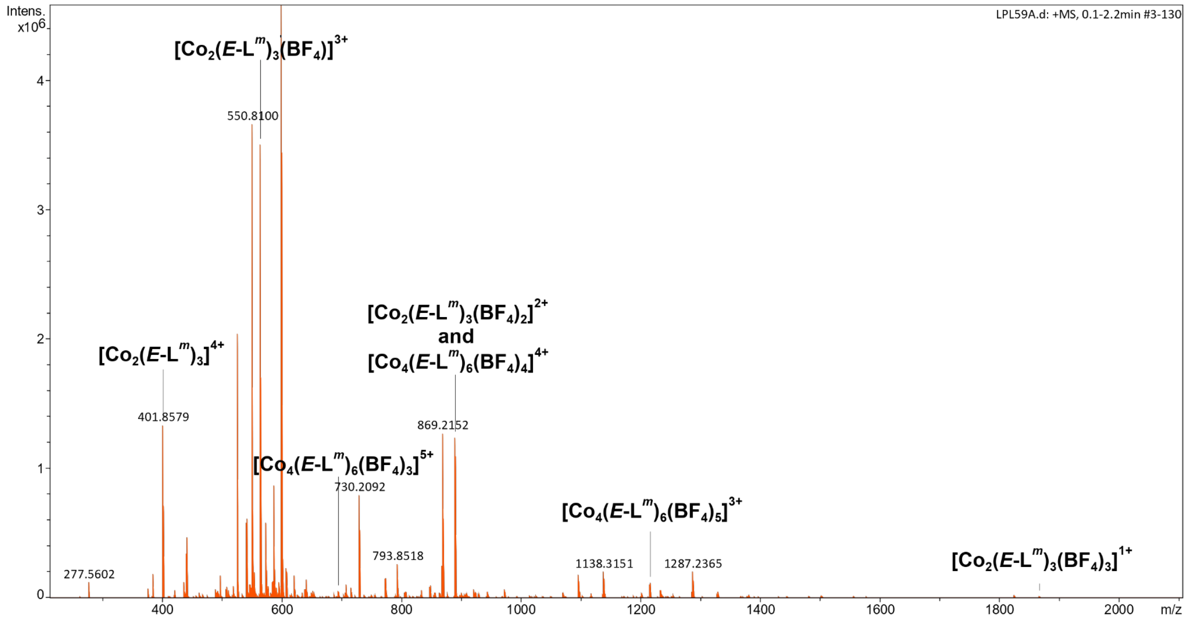

Figure S18. HR-ESI-MS of *E*•**Co**•**L*^{m}*** in CH_3CN with peaks labelled for the M_4 cyclic species $[Co_4(E\text{-}\mathbf{L}^m)_6(BF_4)_{(n)}]^{(8-n)+}$ (n = 3, 4, 5) and the dinuclear triple helicate $[Co_2(E\text{-}\mathbf{L}^m)_3(BF_4)_n]^{(4-n)+}$ (n = 0, 1, 2, 3).

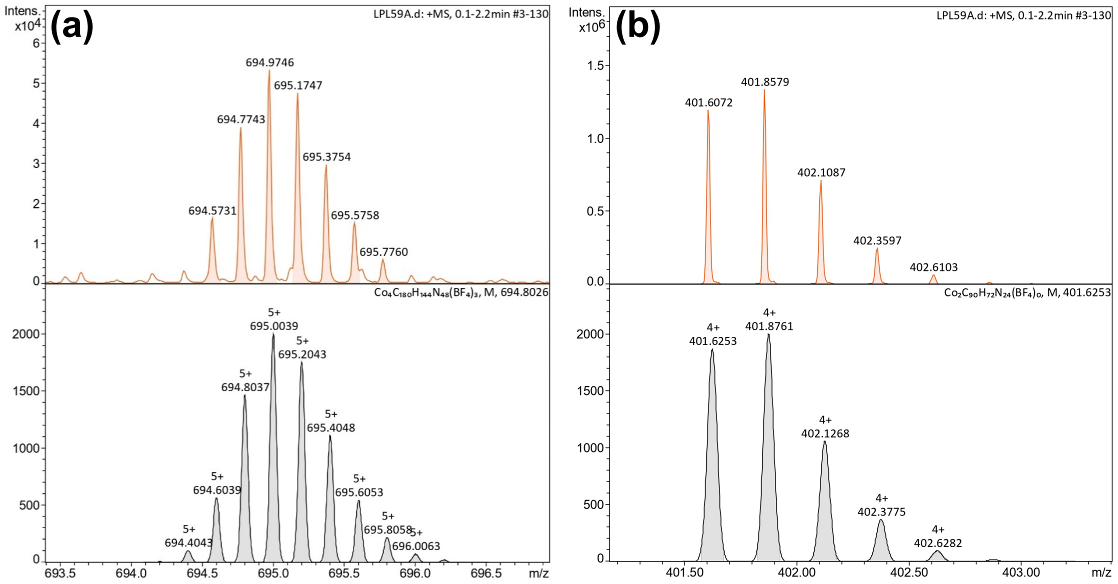

Figure S19. (a) Expansion of $[Co_4(E\text{-}\mathbf{L}^m)_6(BF_4)_3]^{5+}$ with simulated isotope distribution shown below for comparison. (b) Expansion of $[Co_2(E\text{-}\mathbf{L}^m)_3]^{4+}$ with simulated isotope distribution shown below for comparison.

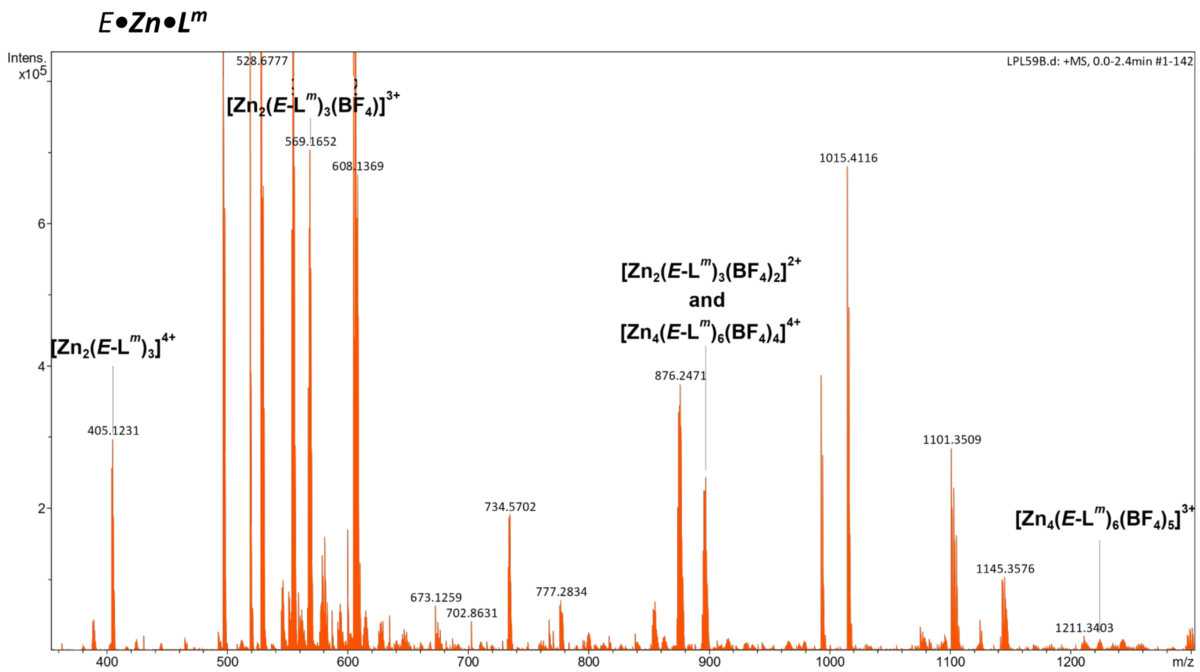

Figure S20. HR-ESI-MS of *E*•**Zn**•**L***m* in CH_3CN with peaks labelled for the M_4 cyclic species $[Zn_4(E\text{-}\mathbf{L}^m)_6(BF_4)_n]^{(8-n)+}$ (n = 4, 5) and the dinuclear triple helicate $[Zn_2(E\text{-}\mathbf{L}^m)_3(BF_4)_n]^{(4-n)+}$ (n = 0, 1, 2).

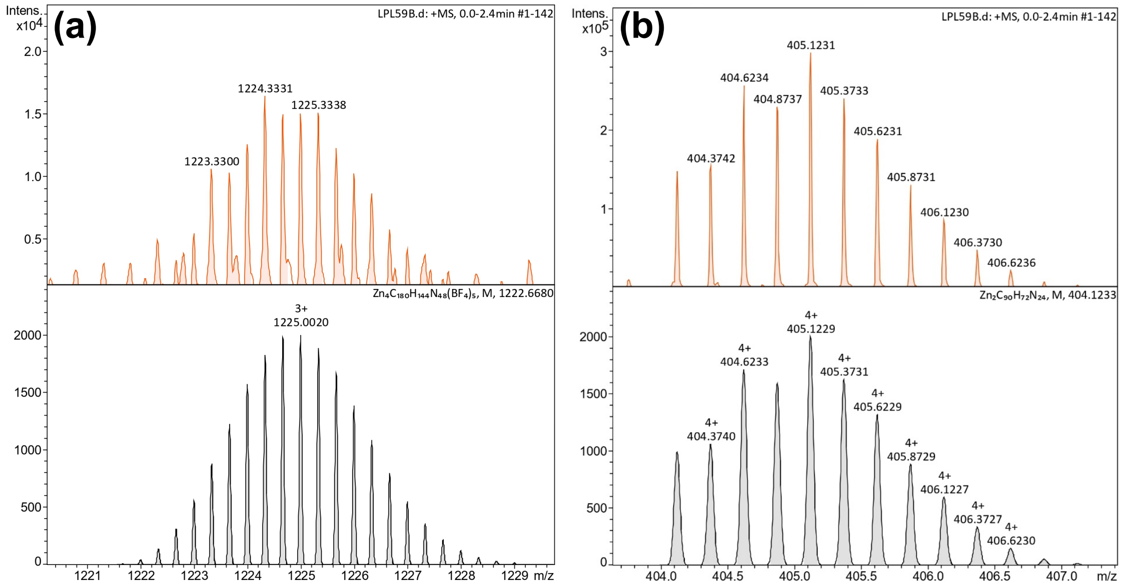

Figure S21. (a) Expansion of $[Zn_4(E\text{-}\mathbf{L}^m)_6(BF_4)_5]^{3+}$ with simulated isotope distribution shown below for comparison. (b) Expansion of $[Zn_2(E\text{-}\mathbf{L}^m)_3]^{4+}$ with simulated isotope distribution shown below for comparison.

Z•**Co**•***L***^{m}

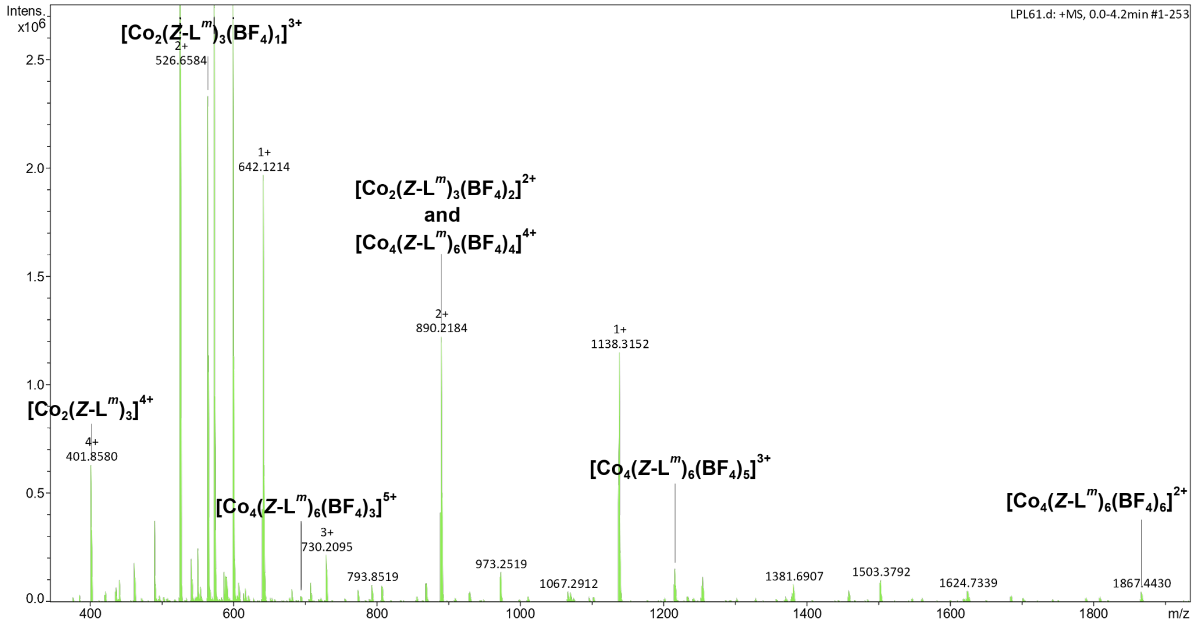

Figure S22. HR-ESI-MS of *Z*•**Co**•**L**^{m} in CH_3CN with peaks labelled for the M4 cyclic species $[Co_4(Z\text{-}\mathbf{L}^m)_6(BF_4)_n]^{(8-n)+}$ (n = 3, 4, 5, 6) and the dinuclear triple helicate $[Co_2(Z\text{-}\mathbf{L}^m)_3(BF_4)_n]^{(4-n)+}$ (n = 0, 1, 2).

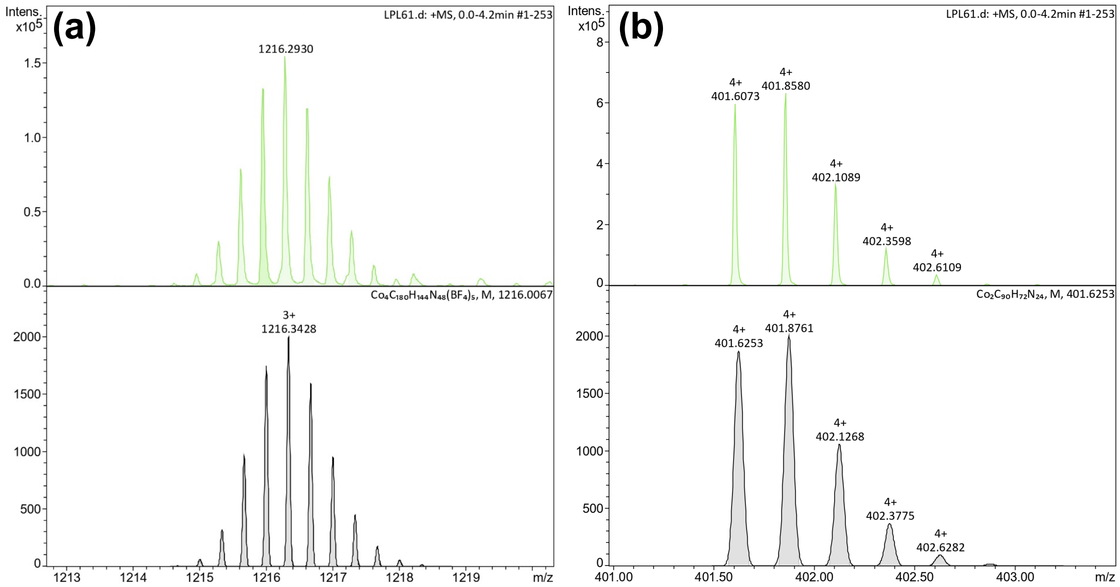

Figure S23. (a) Expansion of $[Co_4(Z\text{-}\mathbf{L}^m)_6(BF_4)_5]^{3+}$ with simulated isotope distribution shown below for comparison. (b) Expansion of $[Co_2(Z\text{-}\mathbf{L}^m)_3]^{4+}$ with simulated isotope distribution shown below for comparison.

Co•L^m•2,4

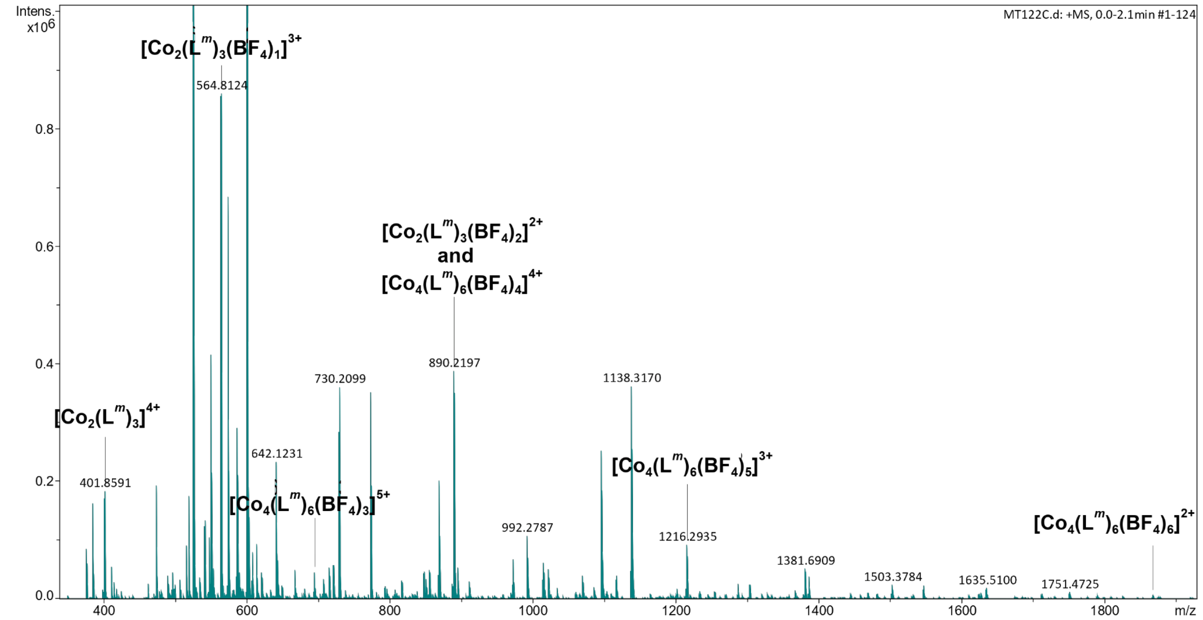

Figure S24. HR-ESI-MS of **Co•$\mathbf{L}^m$•2,4** in CH_3CN with peaks labelled for the M_4 cyclic species $[Co_4(\mathbf{L}^m)_6(BF_4)_n]^{(8-n)+}$ (n = 3, 4, 5, 6) and the dinuclear triple helicate $[Co_2(\mathbf{L}^m)_3(BF_4)_n]^{(4-n)+}$ (n = 0, 1, 2).

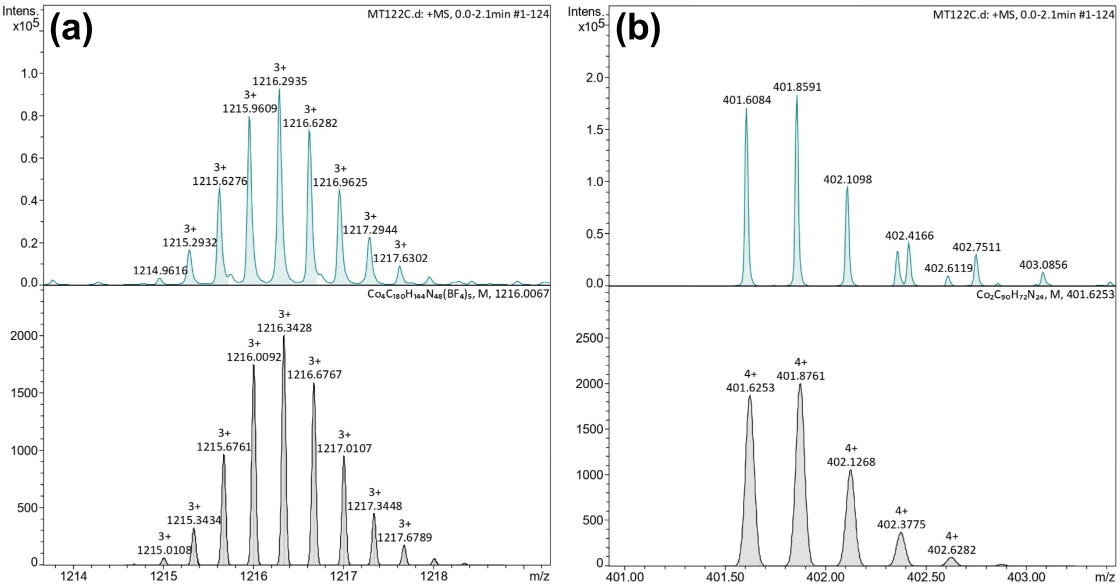

Figure S25. (a) Expansion of $[Co_4(\mathbf{L}^m)_6(BF_4)_5]^{3+}$ with simulated isotope distribution shown below for comparison. (b) Expansion of $[Co_2(\mathbf{L}^m)_3]^{4+}$ with simulated isotope distribution shown below for comparison.

UV/Vis Spectroscopy

L^p

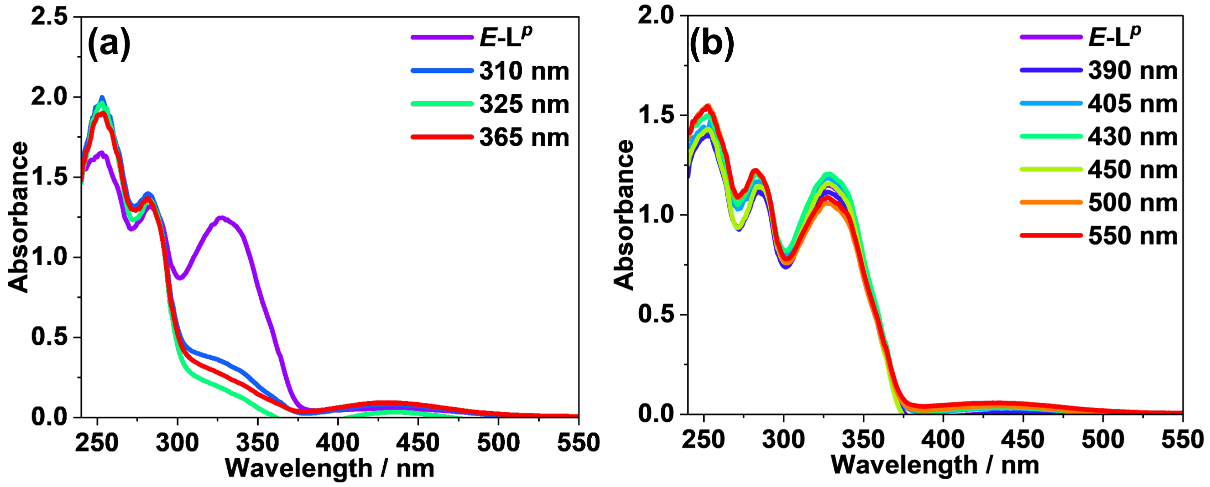

Figure S26. UV-vis spectra of **L**^{p} (MeCN, 55.9 µM) at ambient temperature, after irradiation with different wavelengths (310, 325, 365, 390, 405, 430, 450, 500 and 550 nm, respectively) to determine the optimum wavelengths for photoswitching.

L^m

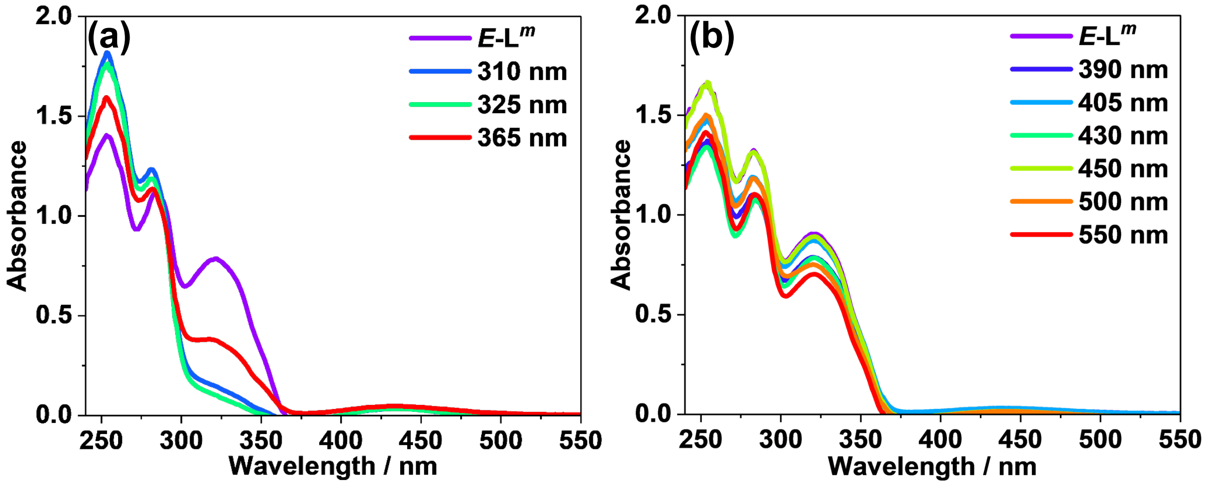

Figure S27. UV-vis spectra of **L**^{m} (MeCN, 56.0 µM) after irradiation with different wavelengths (310, 325, 365, 390, 405, 430, 450, 500 and 550 nm, respectively) to determine the optimum wavelengths for photoswitching.

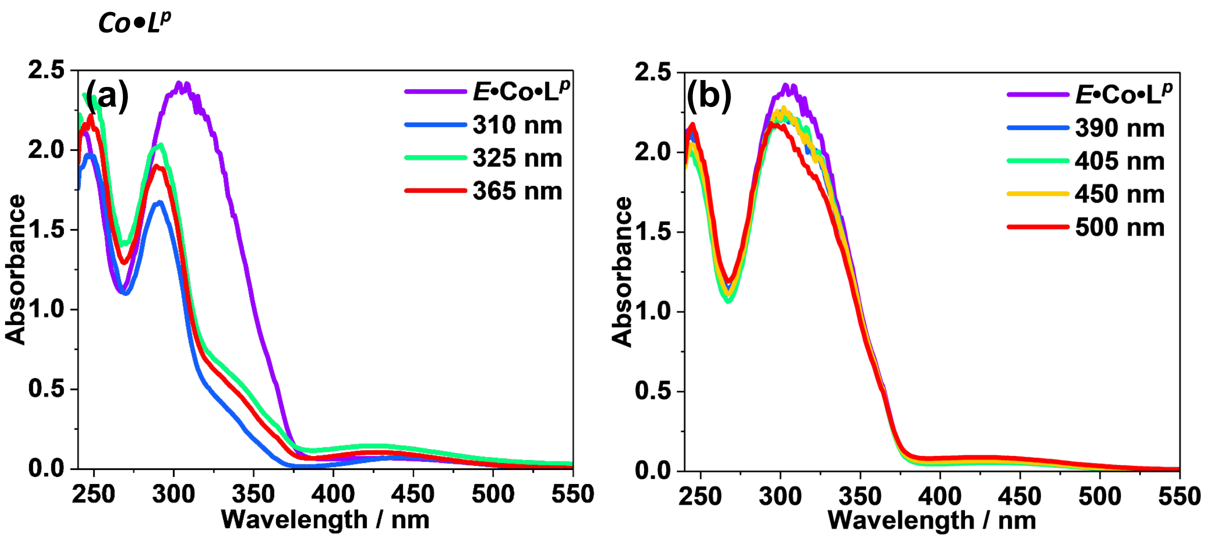

Figure S28. UV-vis spectra of **Co•L**^{p} (MeCN, 28.4 µM) after irradiation with different wavelengths (310, 325, 365, 390, 405, 450, and 500 nm, respectively) to determine the optimum wavelengths for photoswitching.

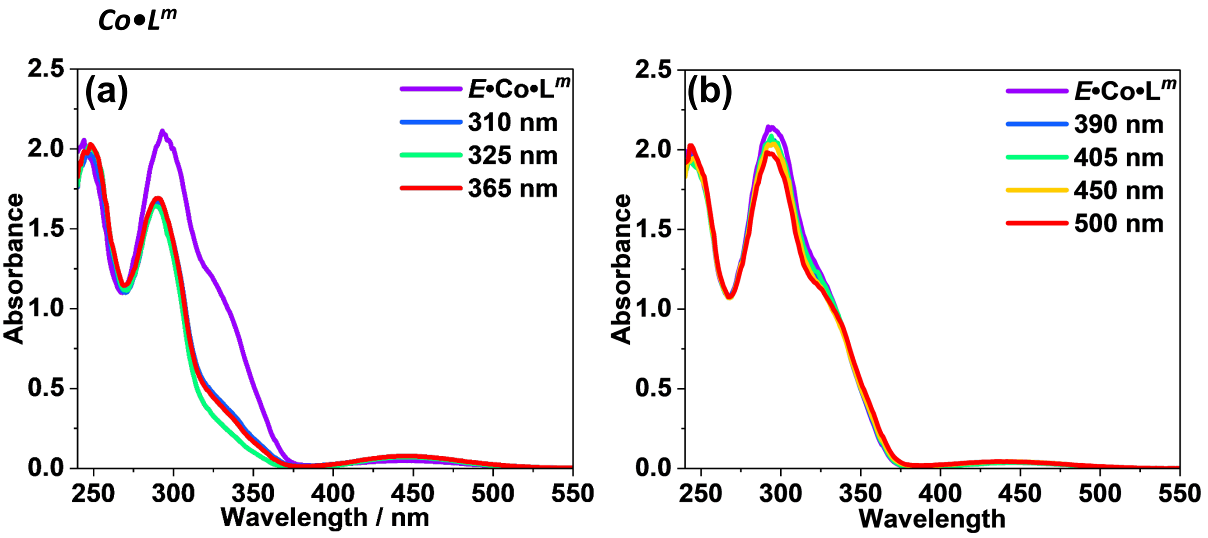

Figure S29. UV-vis spectra of **Co•L**^{m} (MeCN, 14.2 µM) after irradiation with different wavelengths (310, 325, 365, 390, 405, 450, and 500 nm, respectively) to determine the optimum wavelengths for photoswitching.

Variable Temperature NMR

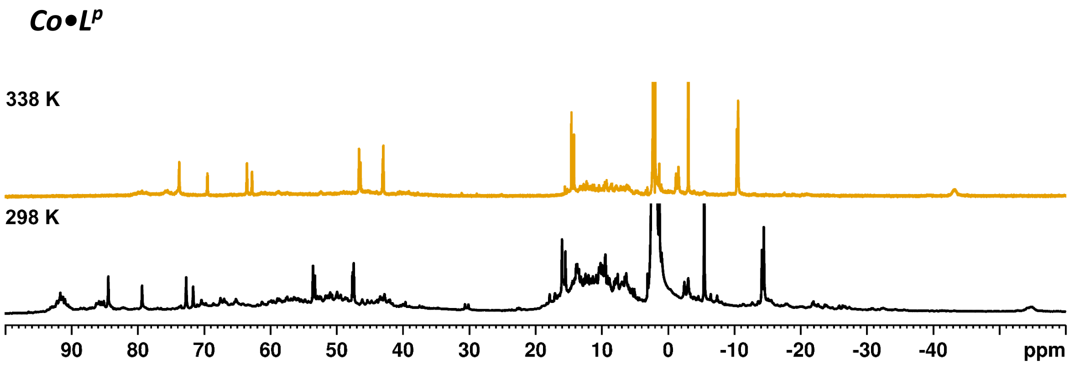

Figure S30. Variable Temperature ^{1}H-NMR spectra (400 MHz, MeCN-d_3, 298, 338 K) of ***E*•Co•L*p*.**

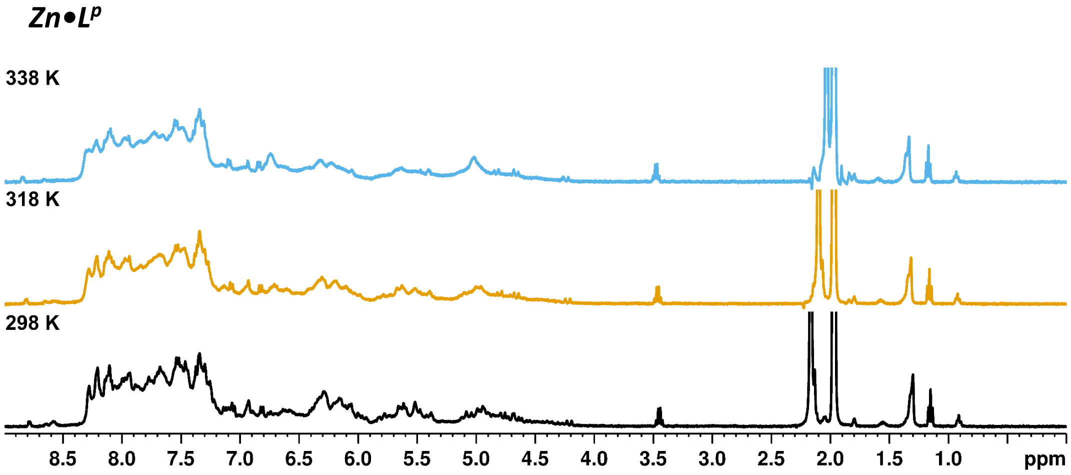

Figure S31. Variable Temperature ^{1}H-NMR spectra (400 MHz, MeCN-d_3, 298, 318, 338 K) of ***E*•Zn•L*p*.**

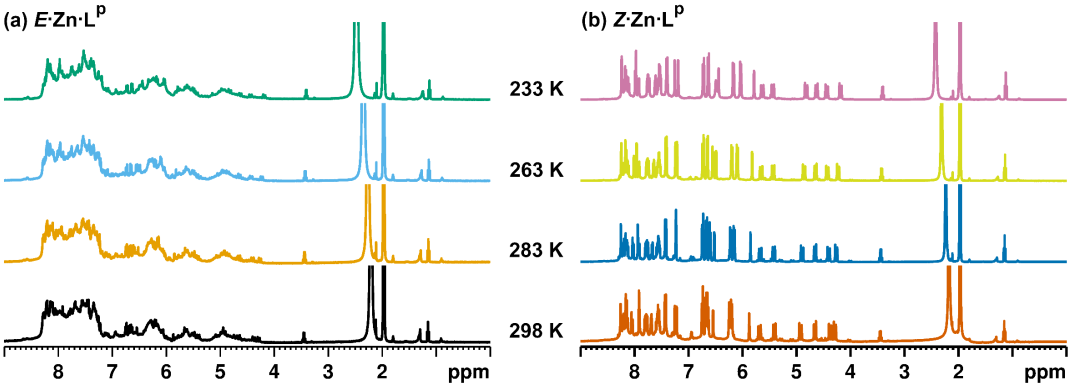

Figure S32. Variable temperature ^{1}H-NMR spectra (400 MHz, MeCN-d_3, 298, 283, 263, 233 K) with a sodium triflate (2 mM) internal standard of (a) *E***•Zn•L*p*** and (b) *Z***•Zn•L*p***.

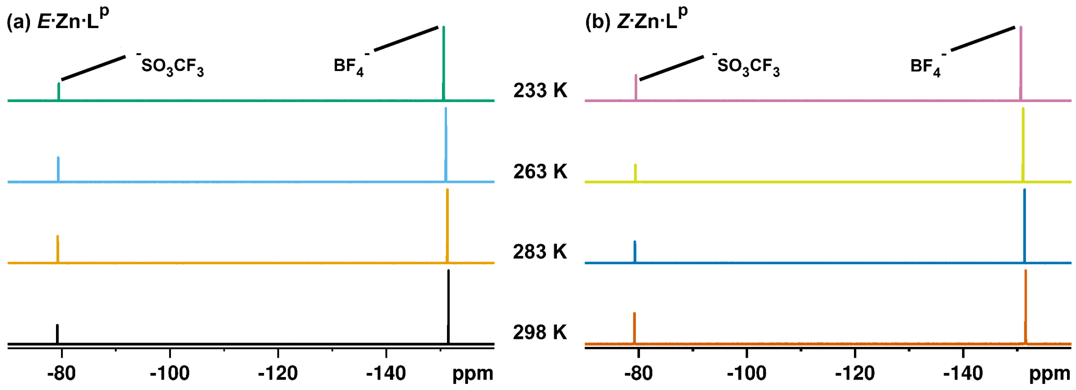

Figure S33. Variable temperature ^{19}F-NMR spectra (400 MHz, MeCN-d_3, 298, 283, 263, 233 K) with a sodium triflate (2 mM) internal standard of (a) *E*•**Zn**•**L**^{p} and (b) *Z*•**Zn**•**L**^{p}.

Thermal Relaxation NMR Experiments

Ligands

Ligands (5 mg, 10 µmol) were dissolved in MeCN-d3 (1 cm^3) and irradiated with 340 nm light until a photostationary state was reached. Spectra were recorded every 30 minutes for 18 hours at 65 °C and every 30 minutes for 60 hours at 25 °C.

Z-**L**^{p} 65 °C

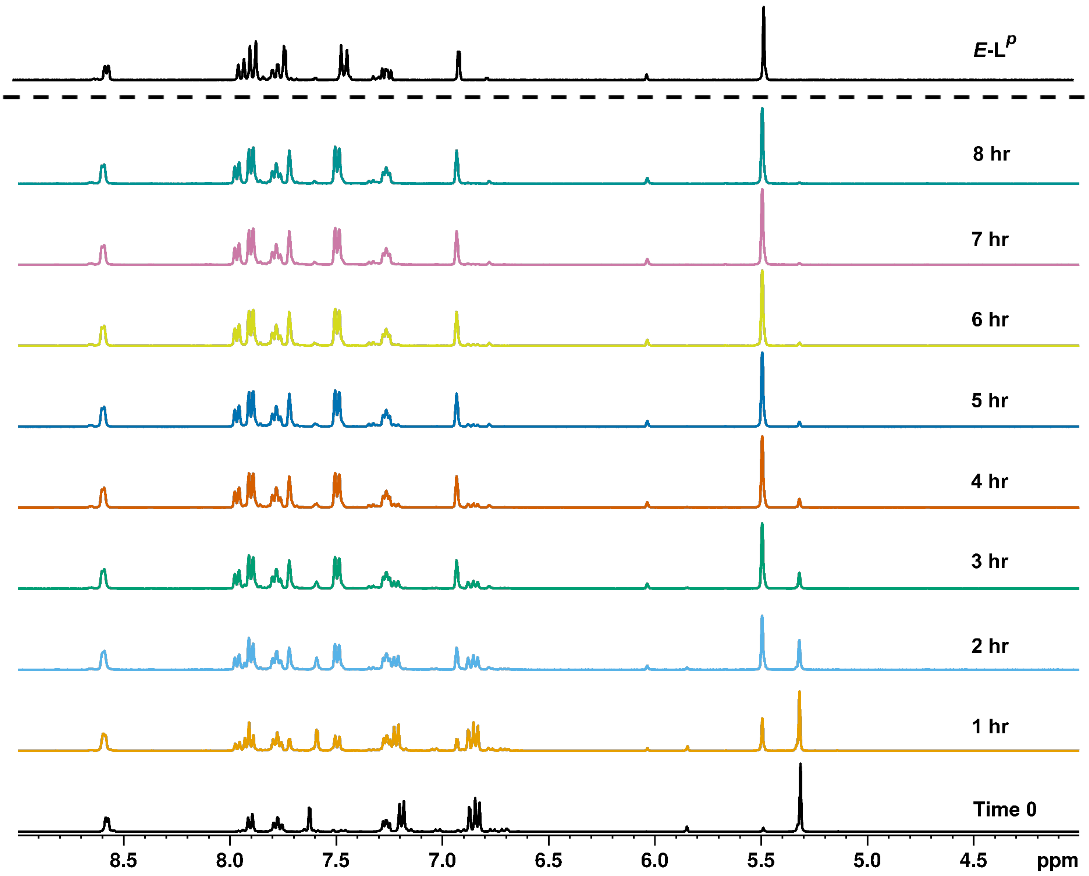

Figure S34. ^{1}H-NMR spectra (400 MHz, MeCN-d3, 338 K) of *Z*-**L**^{p} over 8 hours.

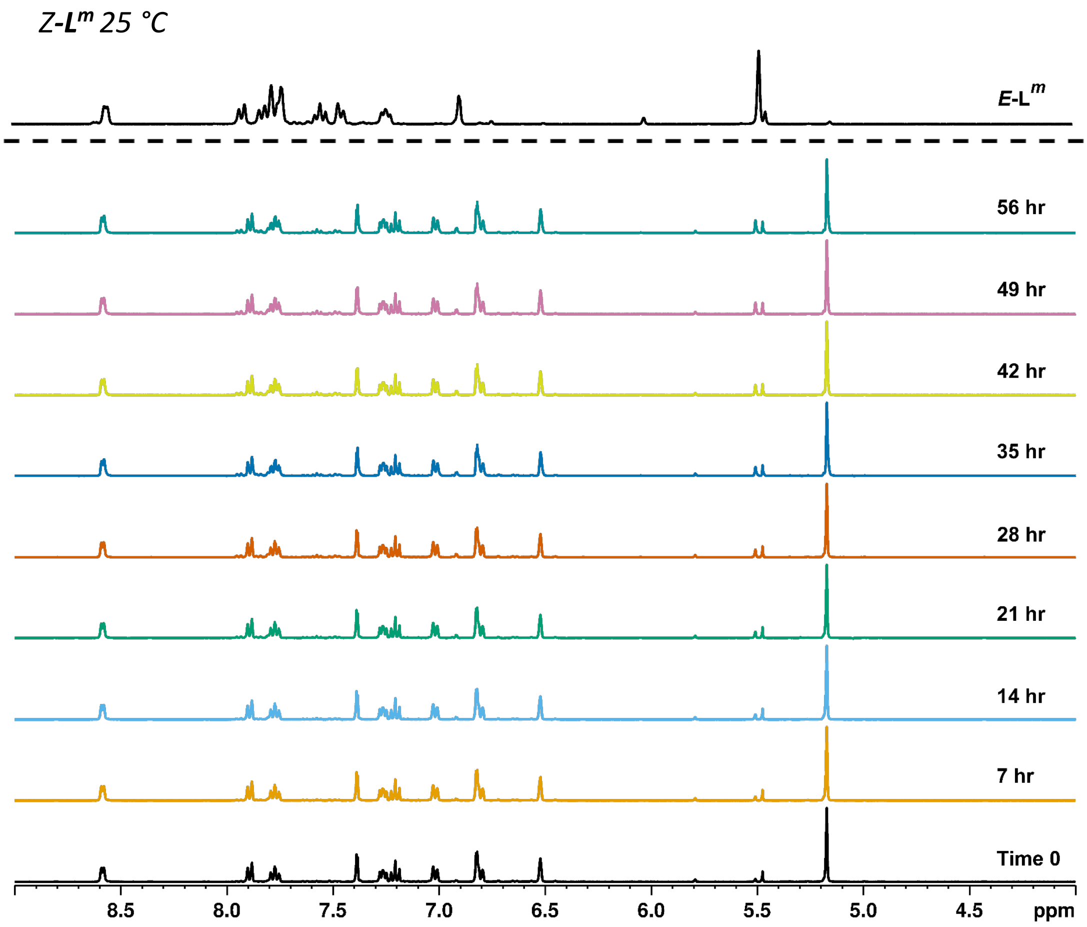

Figure S35. ^{1}H-NMR spectra (400 MHz, MeCN-d_3, 298 K) of *Z*-**L**^{m} over 56 hours.

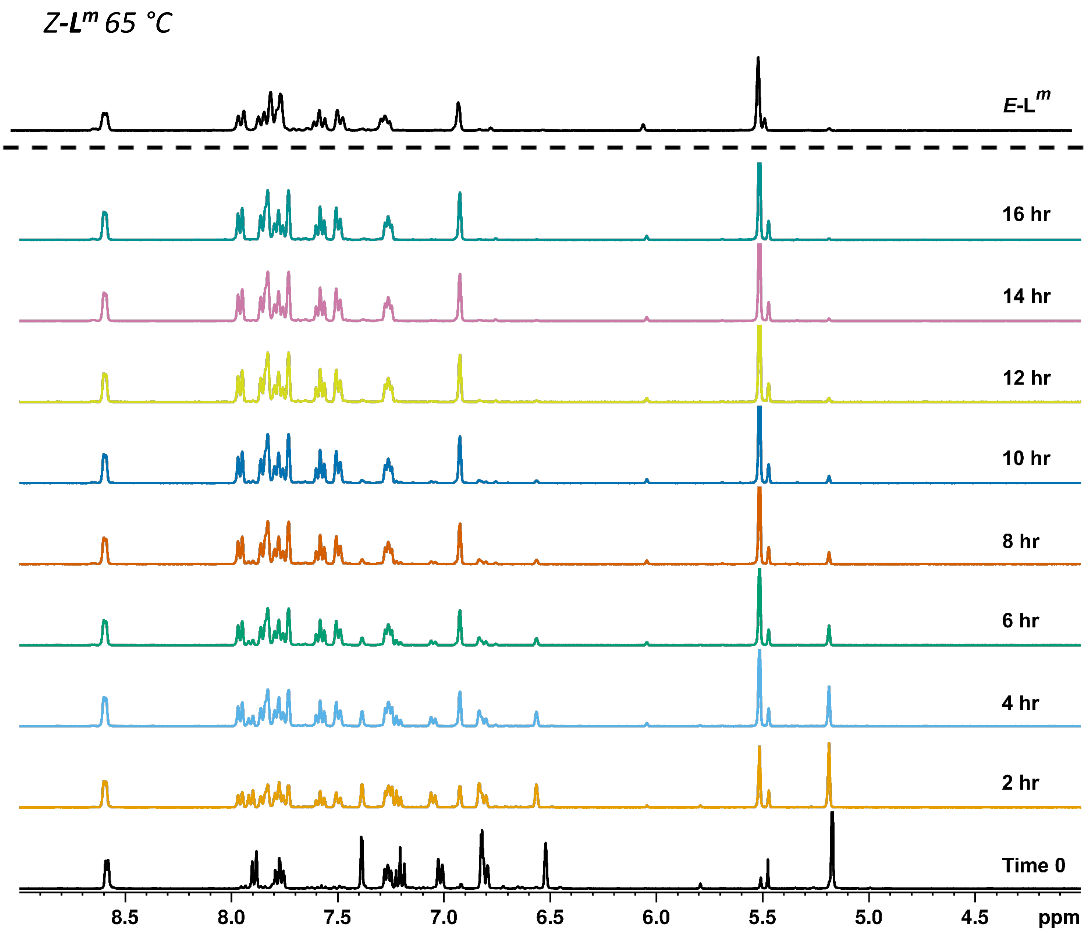

Figure S36. ^{1}H-NMR spectra (400 MHz, MeCN-d$_3$, 338 K) of *Z*-**L**^{m} over 16 hours.

Ligand Half-Life

Z-L^p

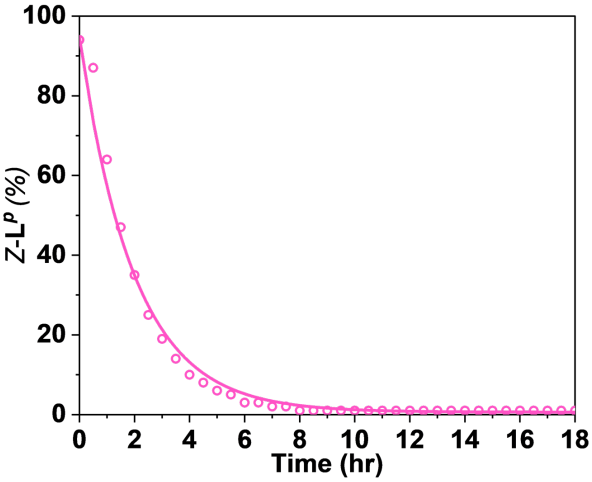

Figure S37. Thermal relaxation of $\mathbf{L}^{p}$ in acetonitrile (2 mM, ^{1}H NMR) at 65 °C after irradiation at 340 nm overnight.

Z-L^m

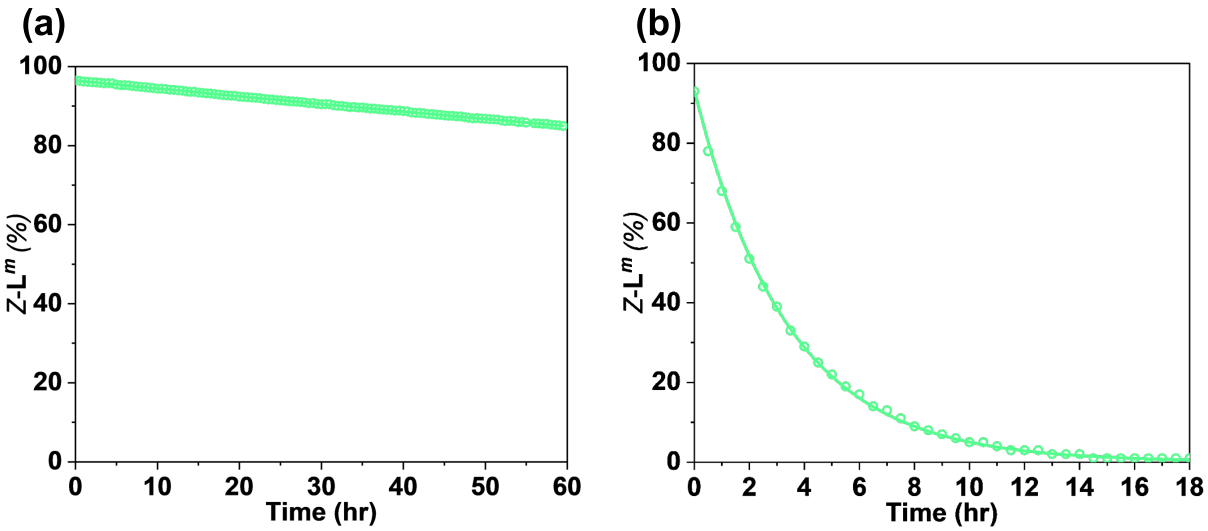

Figure S38. (a) Thermal relaxation of $\mathbf{L}^{m}$ in acetonitrile (2 mM, ^{1}H NMR) at 25 °C after irradiation at 340 nm overnight. (b) Thermal relaxation of $\mathbf{L}^{m}$ in acetonitrile (2 mM, ^{1}H NMR) at 65 °C after irradiation at 340 nm overnight.

Table S2. Rate constants (k), mean lifetimes (τ = k −1), and half-lives ($\tau_{½}$) obtained by thermal relaxation experiments of $\mathbf{L}^{m}$ and $\mathbf{L}^{p}$ (65 °C, MeCN-d_3, 2 mM) using NMR. Relaxation of $\mathbf{L}^{m}$ was also investigated at 25 °C (MeCN-d_3, 2 mM).

	$\mathbf{L}^{p}$ (65 °C)	$\mathbf{L}^{m}$ (25 °C)	$\mathbf{L}^{m}$ (65 °C)
$\tau_{½}$ (hr)	1.38	326.36	2.36
**τ** (hr)	1.99	470.84	3.40
**k** (hr^{-1})	0.504	0.002	0.294

Cages

Cages – *E*•**Co**•**L**^{p} (2 mg, 1 mM) and *E*•**Co**•**L**^{m} (2 mg, 0.5 mM) – were dissolved in MeCN-d$_3$ (1 cm^3) and irradiated with 340 nm light until a photostationary state was reached. Spectra were recorded at a series of time points of 5 weeks, with the samples stored in darkness at ambient temperature between time points.

***Z*•Co•L^{p}**

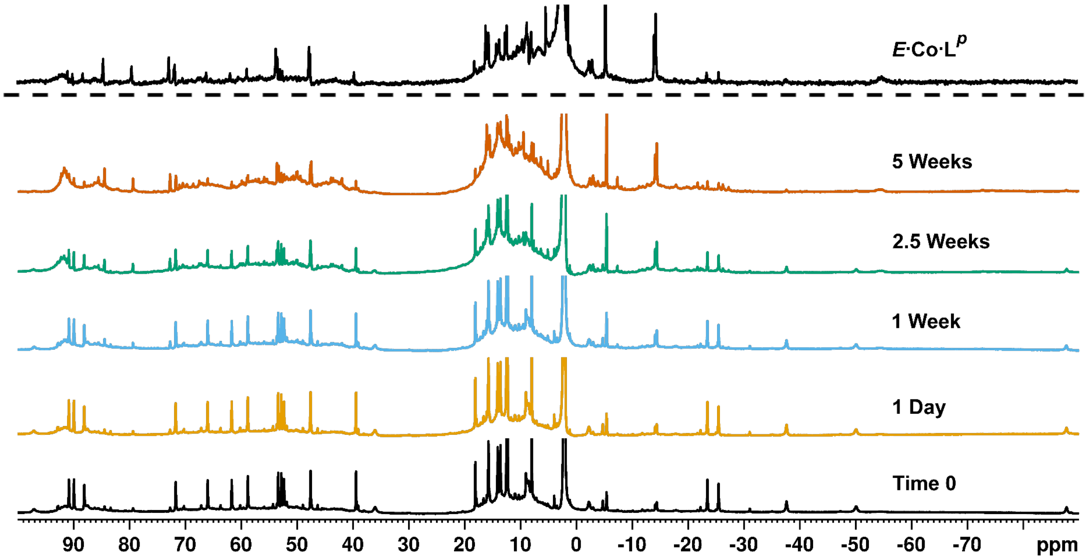

Figure S39. Series of ^{1}H NMR spectra (400 MHz, CD_3CN, MeCN-d$_3$, RT) showing the slow thermal conversion of *Z*•**Co**•**L**^{p} (bottom spectrum) back to *E*•**Co**•**L**^{p} (top spectrum) over 5 weeks.

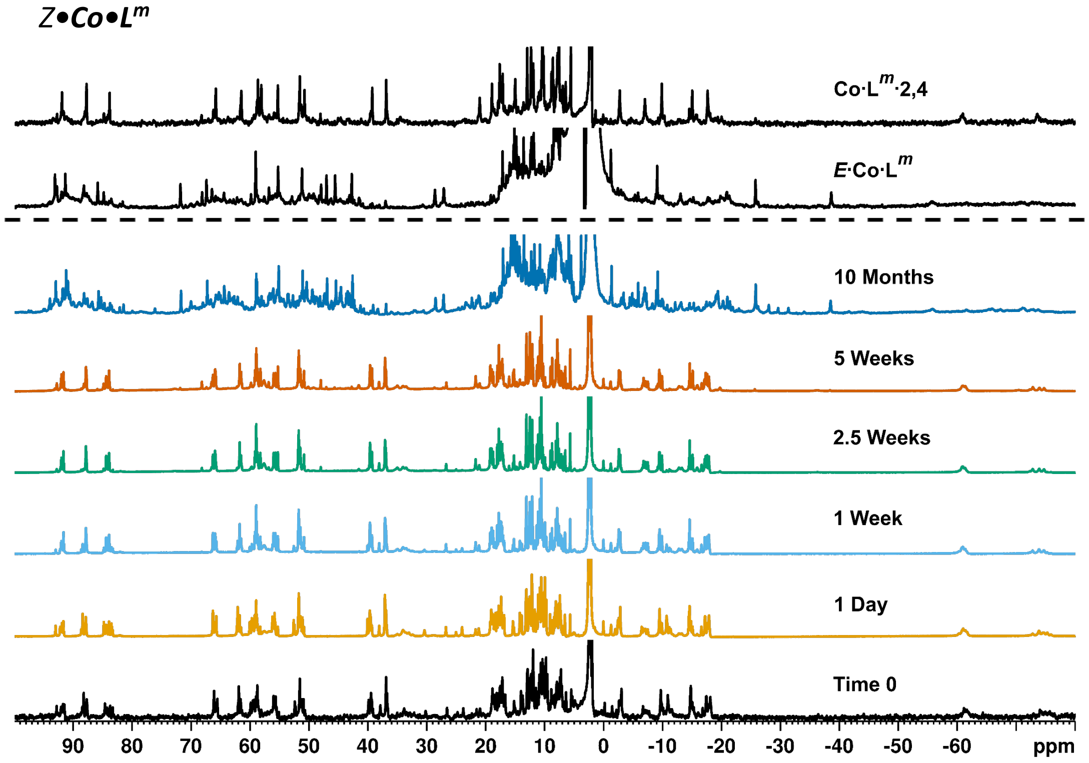

Figure S40. Series of ^1H NMR spectra (400 MHz, CD_3CN, MeCN-d_3, RT) showing the slow thermal conversion of *Z*•**Co•L***^{m}* (bottom spectrum) back to *E*•**Co•L***^{m}* (top spectrum) over 5 weeks.

Titrations

Mixed Ligand Cage Formation

E-**L***m* (30 mg, 60 µmol) was dissolved in a $CDCl_3$:MeCN-d_3 (85:15 *v/v*, 6 cm^3) mixture. 3 cm^3 of the ligand solution was irradiated with 340 nm light until a photostationary state was reached. A series of NMR tubes were prepared, containing 0.7 cm^3 of solution by combining the two samples in various ratios and NMR spectra recorded. $Co(BF_4)_2\cdot6H_2O$ (16 mg, 47 µmol) was dissolved in $CDCl_3$:MeCN-d_3 (85:15 *v/v*, 1 cm^3). 0.1 cm^3 of metal salt solution was added to each tube and spectra recorded.

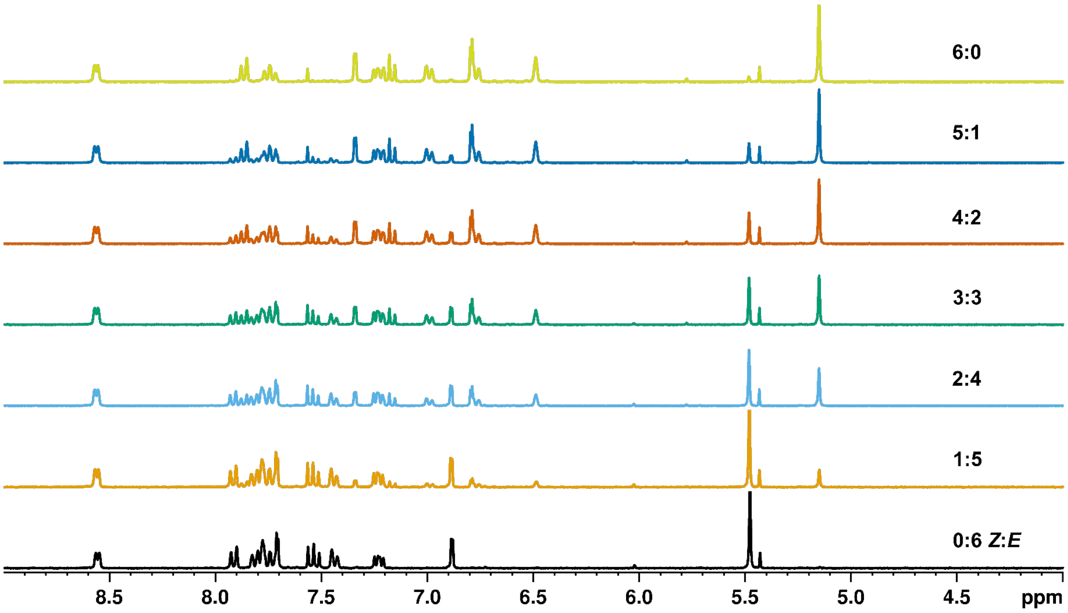

Figure S41. A series of ^{1}H-NMR spectra (300 MHz, MeCN-d_3/$CDCl_3$ (85:15 *v/v*), 298 K) recorded during the combination of *E*-**L***m* (10 mM) and *Z*-**L***m* (10 mM) in the specified ratios.

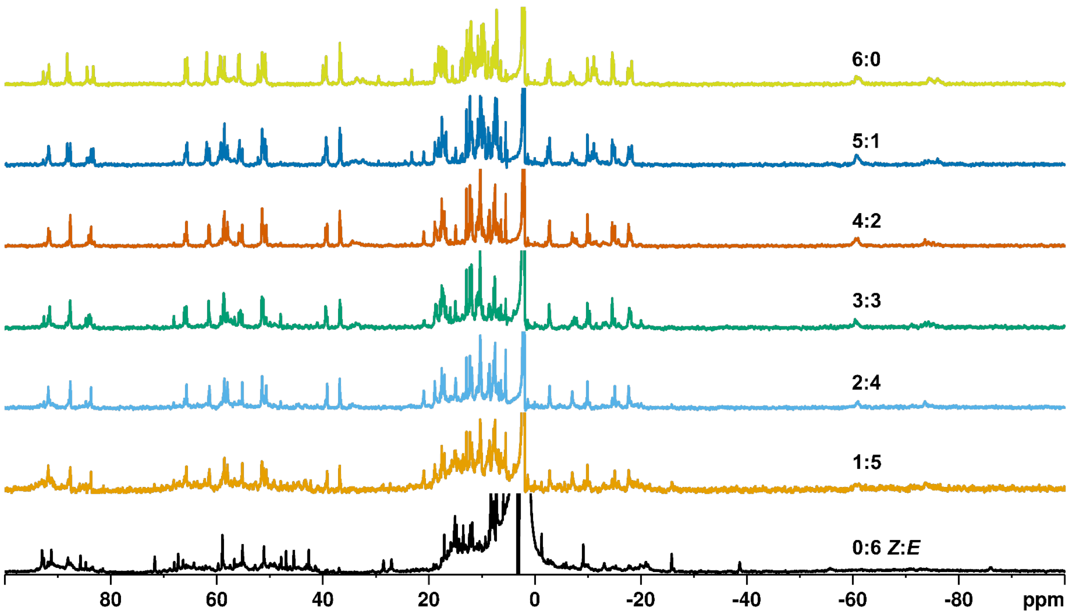

Figure S42. A series of ^{1}H-NMR spectra (300 MHz, MeCN-d_3/$CDCl_3$ (85:15 *v/v*), 298 K) recorded during the combination of a series of mixtures, containing *E*-**L*m*** and *Z*-**L*m*** in the specified ratios, with $Co(BF_4)_2$.

*Addition of Z-**L**^{m} to E•**Co**•**L**^{m}*

E-**L**^{m} (15 mg, 30 µmol) was dissolved in $CDCl_3$:MeCN-d_3 (85:15 *v/v*, 3 cm^3) and irradiated with 340 nm light. *E*•**Co**•**L**^{m} (62.5 mg, 16 µmol) was dissolved in $CDCl_3$:MeCN-d_3 (85:15 *v/v*, 2 cm^3). 0.1 cm^3 of cage solution was added to a series of NMR tubes. *Z*-**L**^{m} solution and $CDCl_3$:MeCN-d_3 (85:15 *v/v*) were added to each tube in various ratios to form 0.7 cm^3 samples containing excess ligand and NMR spectra recorded.

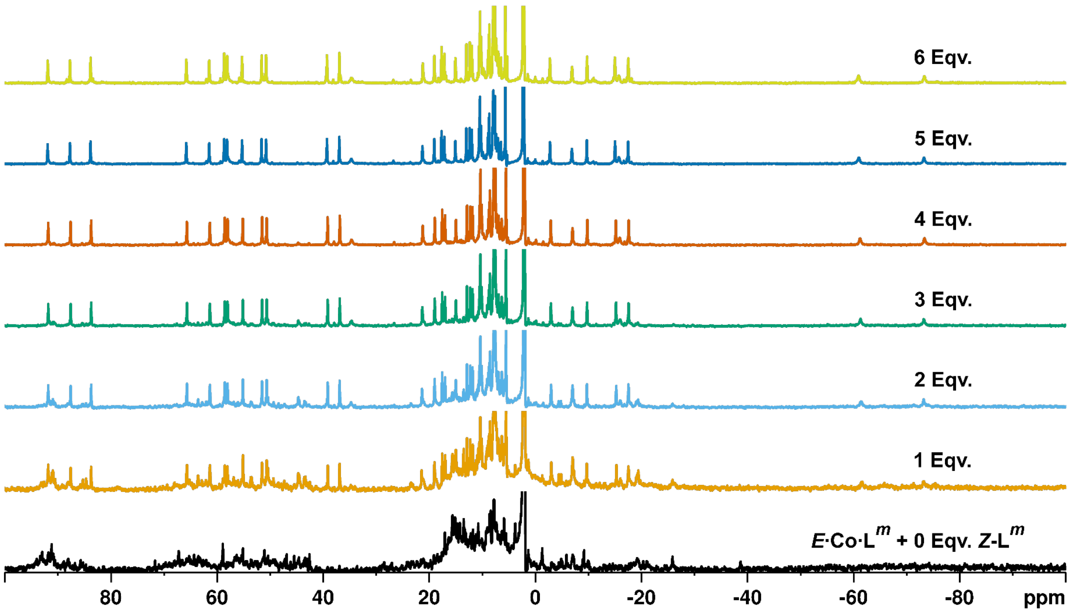

Figure S43. ^{1}H NMR spectra (300 MHz, MeCN-d_3/$CDCl_3$ (85:15 v/v), 298 K) recorded during addition of 0 – 6 equivalents (from bottom up) of *Z*-**L**^{m} to a solution of *E*•**Co**•**L**^{m}.

*Addition of E-**L**^m to Z•**Co**•**L**^m*

E-**L**^m (16 mg, 32 µmol) was dissolved in $CDCl_3$:MeCN-d_3 (85:15 *v/v*, 3.2 cm^3) and irradiated with 340 nm light overnight. $Co(BF_4)_2 \cdot 6H_2O$ (7.3 mg, 21 µmol) was added to form *Z*•**Co**•**L**^m. 0.43 mL of *Z*•**Co**•**L**^m solution was added to a series of NMR tubes. *E*-**L**^m (18 mg, 36 µmol) was dissolved in $CDCl_3$:MeCN-d_3 (85:15 *v/v*, 3 cm^3). *E*-**L**^m solution and $CDCl_3$:MeCN-d_3 (85:15 *v/v*) were added to the NMR tubes in various ratios to form 0.7 cm^3 samples such that *E*-**L**^m was in excess and NMR spectra recorded.

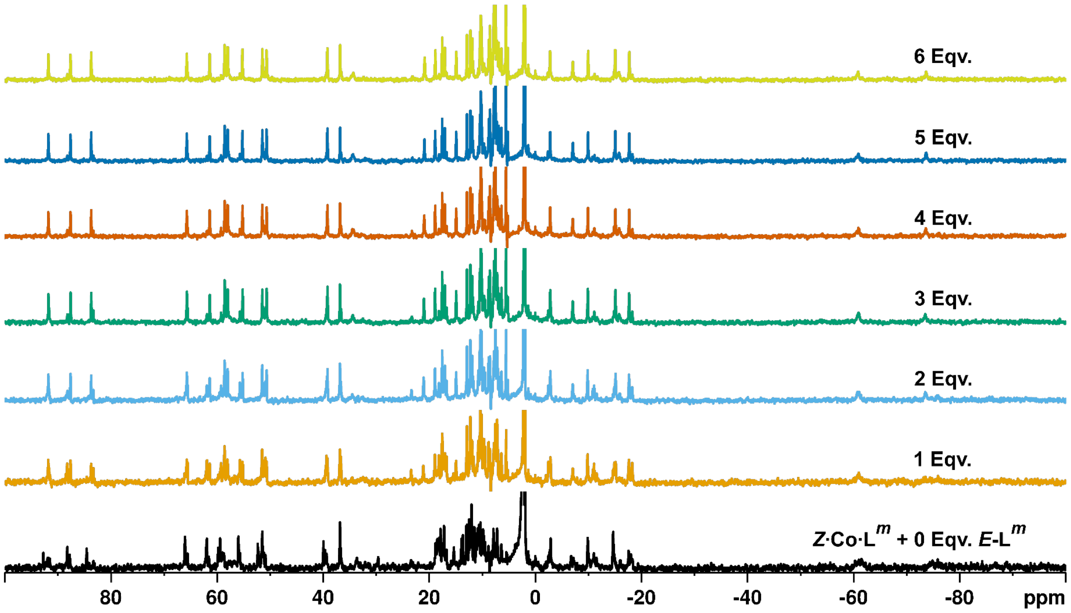

Figure S44. ^{1}H NMR spectra (300 MHz, MeCN-d_3/$CDCl_3$ (85:15 v/v), 298 K) recorded during addition of 0 – 6 equivalents (from bottom up) of *Z*-**L**^m to a solution of *E*•**Co**•**L**^m.

In situ NMR Spectra

We have adapted this method from literature[4] and described it previously.[5] We add this description here for convenience:

The NMR sample used for the *in-situ* illumination experiments was fitted with an insert tube made from quartz glass. A quartz glass optical fibre with a non-terminated end and its exposed surface and the exposed surface roughened to ensure even and omnidirectional illumination was subsequently inserted into the insert. In order to avoid damaging the fibre, an aluminium rod was used to lower this construction into the NMR spectrometer. The opposite end of the optical fibre was connected to the light source to allow for the irradiation of the sample inside the spectrometer.[4]

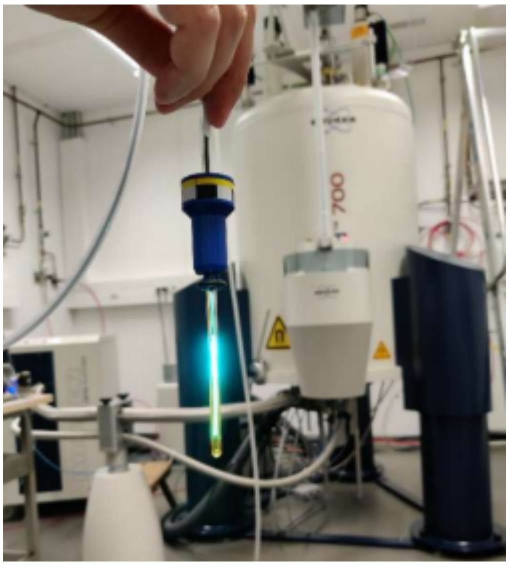

Figure S45. Visual presentation of the in-situ NMR irradiation set up: An NMR sample with a quartz-glass insert and an optical fibre.[5]

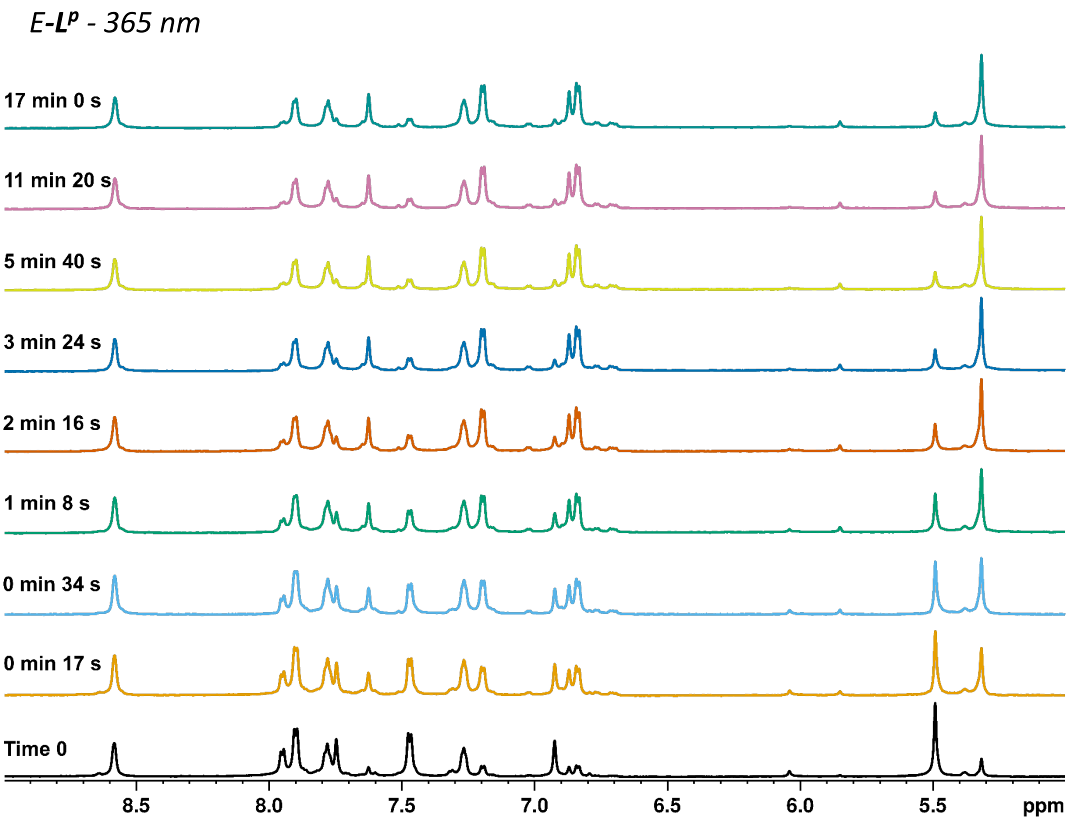

Figure S46. Series of ^{1}H-NMR (700 MHz, CD_3CN, 298 K) spectra during illumination of *E*-**L**^{p} with 365 nm light for 17 minutes.

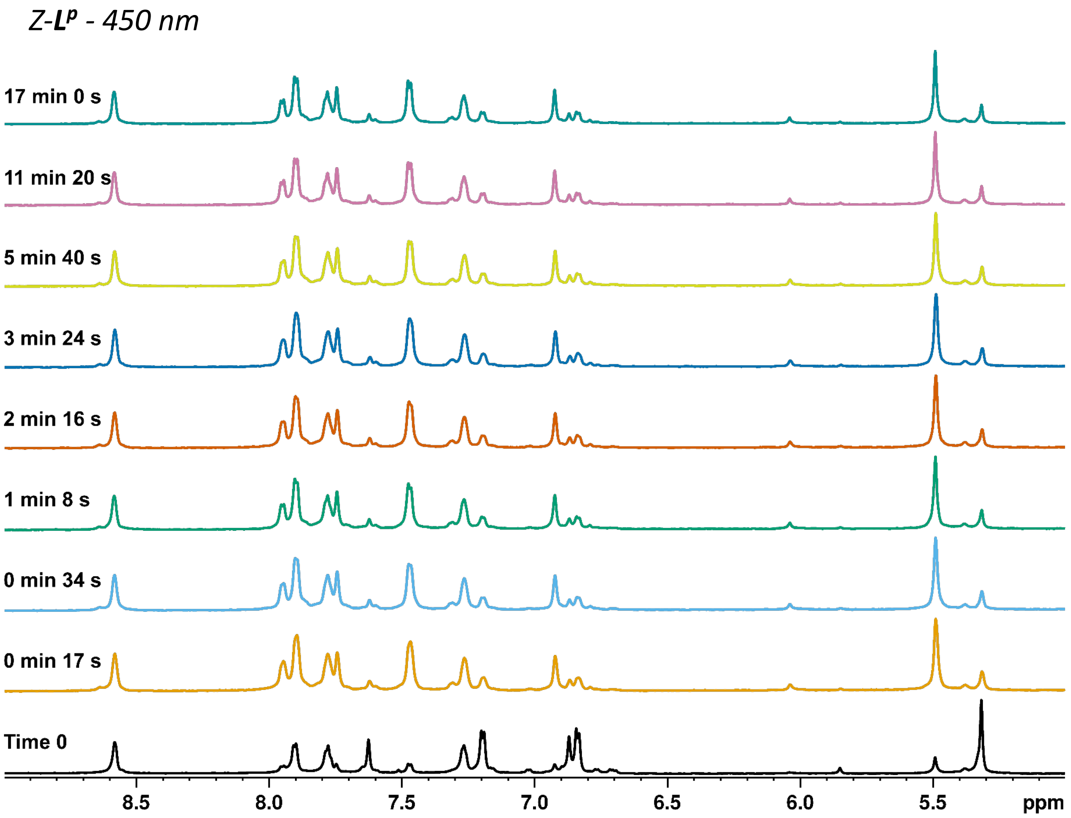

Figure S47. Series of ^{1}H-NMR (700 MHz, CD_3CN, 298 K) spectra during illumination of *Z*-**L**^{p} with 450 nm light for 17 minutes.

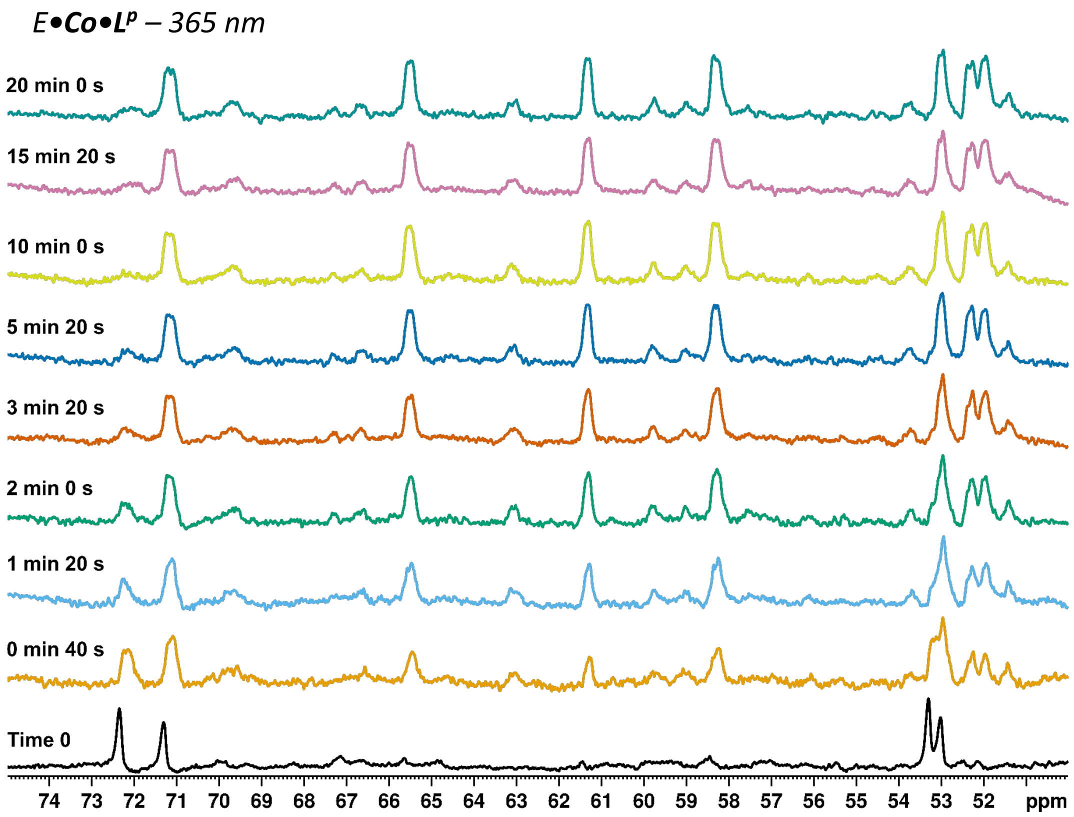

Figure S48. Series of ^{1}H-NMR (700 MHz, CD_3CN, 298 K) spectra during illumination of ***E*•Co•L^p** with 365 nm light for 20 minutes.

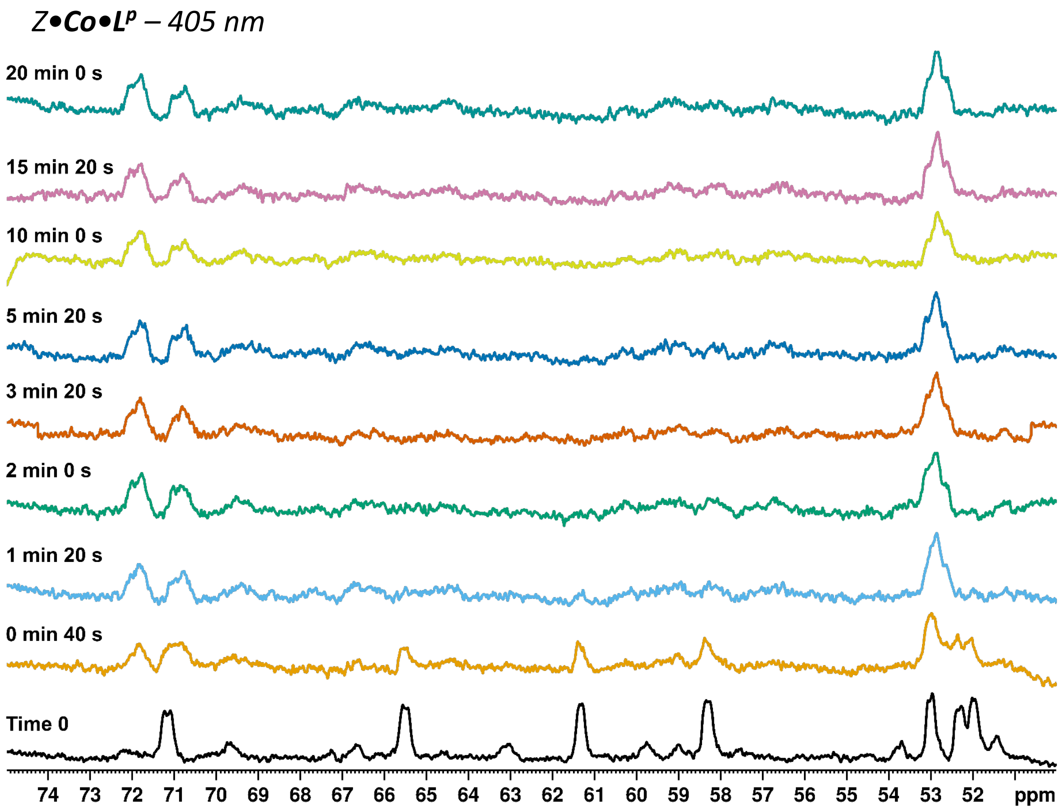

Figure S49. Series of ^{1}H-NMR (700 MHz, CD_3CN, 298 K) spectra during illumination of ***Z*•Co•L^p** with 405 nm light for 20 minutes.

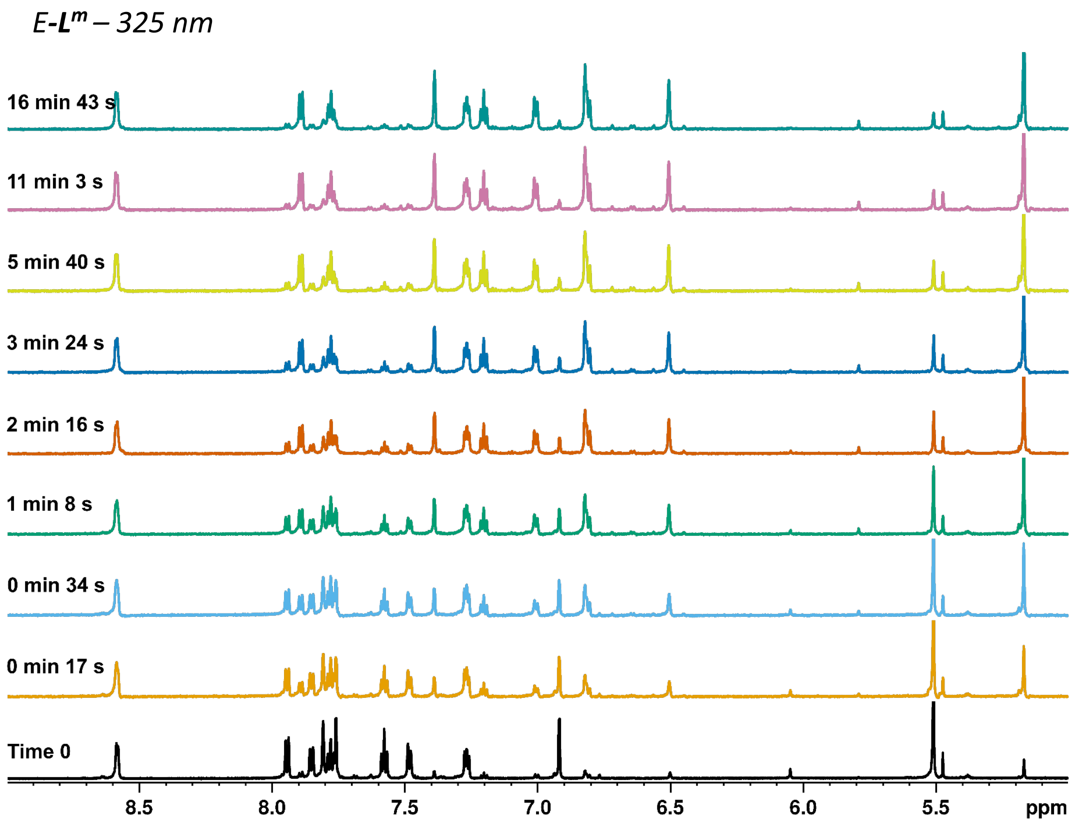

Figure S50. Series of ^{1}H-NMR (700 MHz, CD_3CN, 298 K) spectra during illumination of *E*-**L**^{m} with 325 nm light for 17 minutes.

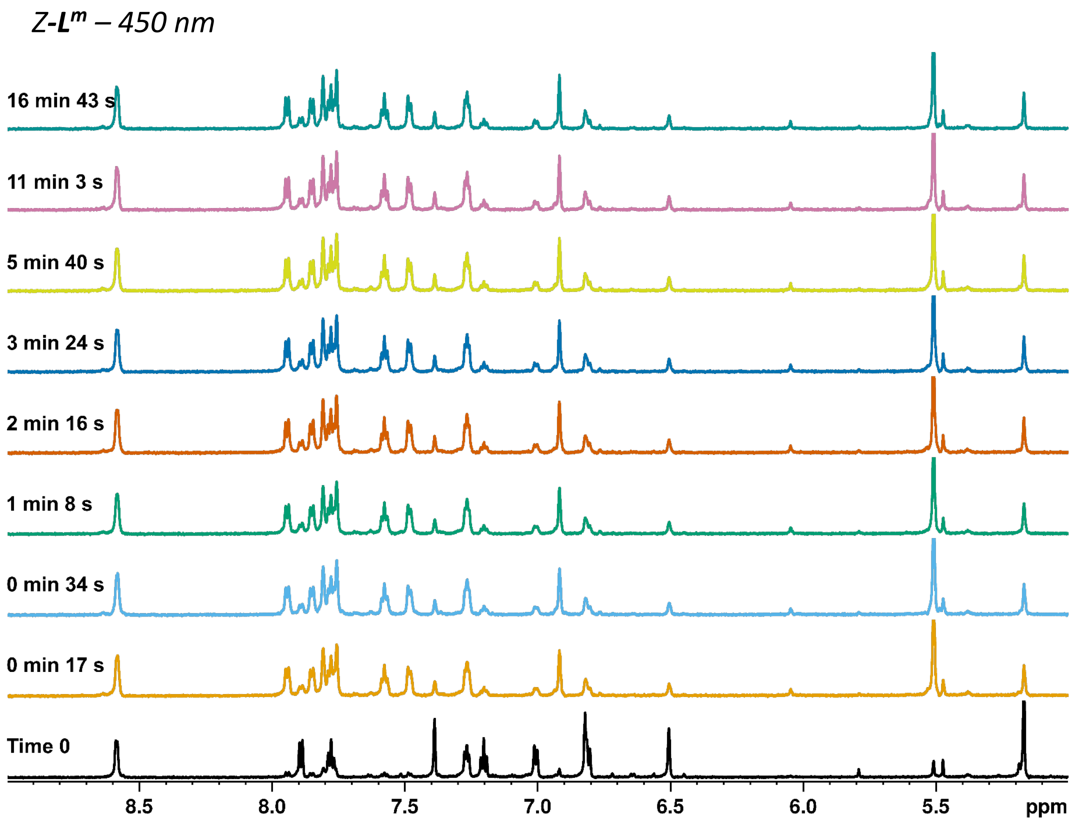

Figure S51. Series of ^{1}H-NMR (700 MHz, CD_3CN, 298 K) spectra during illumination of *Z*-**L**^{m} with 450 nm light for 17 minutes.

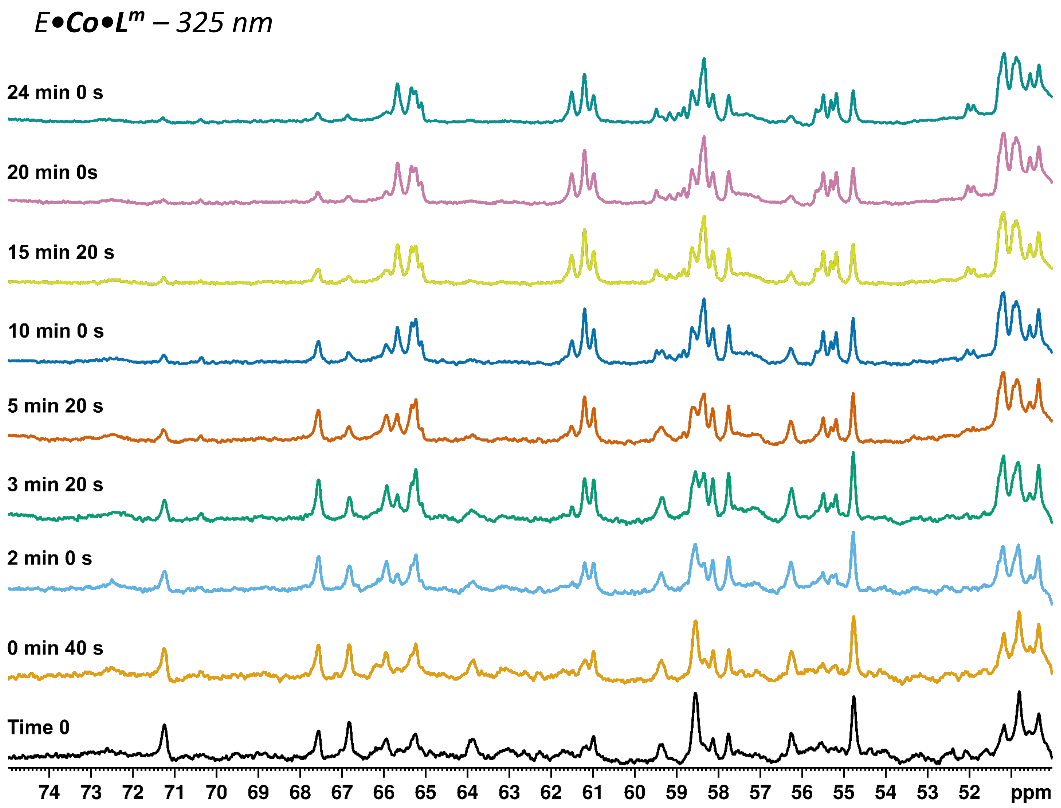

Figure S52. Series of ^{1}H-NMR (700 MHz, CD_3CN, 298 K) spectra during illumination of ***E*•Co•L*m*** with 325 nm light for 24 minutes.

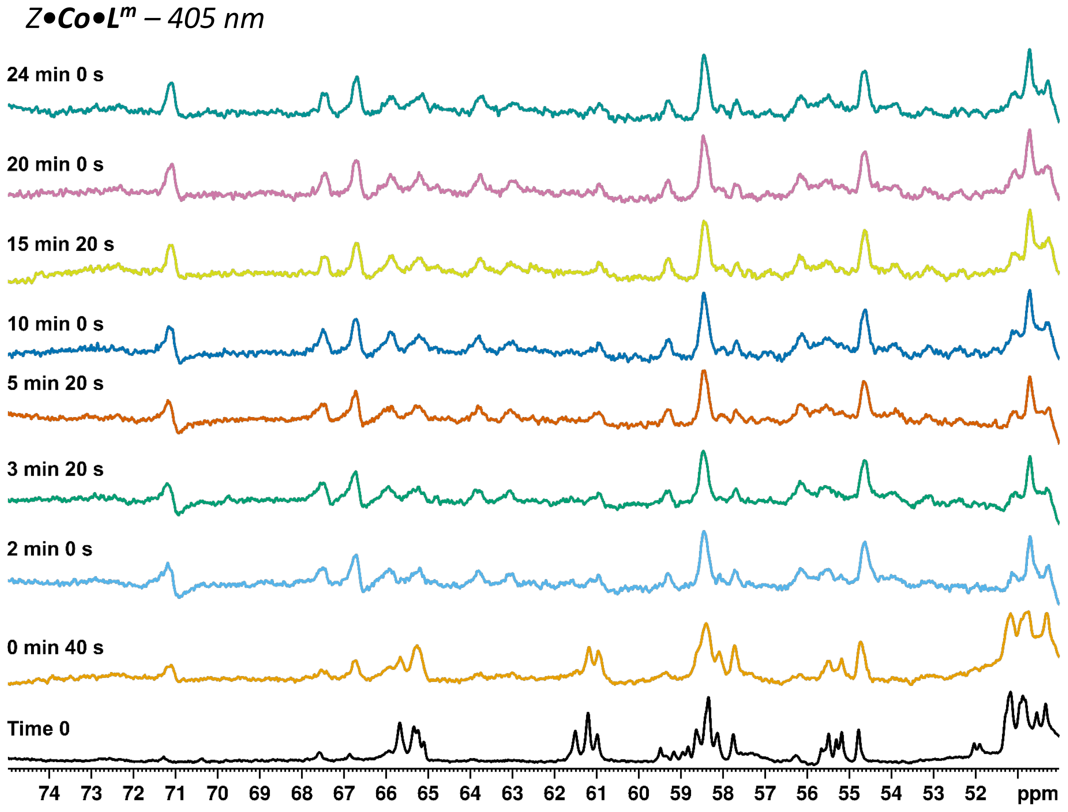

Figure S53. Series of ^{1}H-NMR (700 MHz, CD_3CN, 298 K) spectra during illumination of ***Z*•Co•L*m*** with 405 nm light for 24 minutes.

X-ray crystallography

Details of the crystals used, data collection and refinements are given in Table 1. The structural determination of ligand $\mathbf{L}^{\boldsymbol{m}}$ was performed on a Rigaku Supernova diffractometer. The data was integrated and absorption corrections were applied using a Gaussian numerical method using the CrysAlisPro software.[6]

The data collection for *E*•**Co**•$\mathbf{L}^{\boldsymbol{m}}$ was performed in Experiment Hutch 1 of beamline I-19 at the UK Diamond Light Source synchrotron facility,[7] using methodology, data processing and software described previously.[1h] The structures were solved with Olex2,[8] using dual space iterative methods (SHELXT)[9] and refined by a full-matrix least-squares algorithm (SHELXL).[9]

As is usual with crystallographic structure determinations of this kind of elaborate supramolecular assemblies, for *E*•**Co**•$\mathbf{L}^{\boldsymbol{m}}$ scattering is weak and refinement problems are significant due to substantial disorder, principally of anions and solvent molecules, although the Co_4L_6 cage superstructure itself showed disorder of some ligand fragments over two closely-spaced positions. These problems required (i) extensive use of restraints to ensure geometrically reasonable structures (in particular: the geometries of pyrazole and pyridine rings were fixed with AFIX commands due to high disorder, and rigid bond (RIGU) and similarity (SIMU) restraints were applied to the anisotropic displacement parameters of all atoms in the structure; and (ii) elimination of regions of diffuse electron density using the solvent mask feature in OLEX leaving apparent voids in the lattices equating to 513 electrons *per* complex molecule. Details pertaining to each structure are included in the individual CIFs. Discussion of the structure of *E*•**Co**•$\mathbf{L}^{\boldsymbol{m}}$ in the main text is accordingly at the level of demonstrating the gross geometry of the complex with detailed discussion of structural minutiae kept to a minimum.

Structure name	$\mathbf{L}^{\boldsymbol{m}}$	*E*•**Co**•$\mathbf{L}^{\boldsymbol{m}}$
CCDC number	2301496	2301498
Empirical formula	$C_{30}H_{24}N_8$	$B_{7.94}C_{191.88}Co_4F_{32.28}H_{203.94}N_{48.11}O_{17.88}$
Formula weight	496.57	4405.01
T / K	100	100
Radiation	Cu Kα (λ = 1.54184)	Synchrotron (λ = 0.6889 Å)
Crystal system	monoclinic	triclinic
Space group	$P2_1/n$	*P*–1
a/Å	12.4889(8)	20.35937(13)
b/Å	4.8850(3)	21.05549(13)
c/Å	20.2570(12)	23.94804(13)
α/°	90	93.3668(5)
β/°	92.563(6)	93.7613(5)
γ/°	90	94.7042(6)
Volume/Å^3	1234.61(13)	10188.21(8)

Z	2	2
ρ_{calc} / g cm^3	1.336	1.436
μ/mm^{-1}	0.664	0.393
Crystal size/mm^3	0.17 × 0.05 × 0.03	0.10 x 0.08 x 0.07
2Θ range for data collection/°	8.158 – 154.29	1.88 – 45.00
Reflections collected	10836	203895
Independent reflections /R_{int}	2364 / 0.1565	29237 / 0.0390
Data/restraints/ parameters	2364 / 252 / 225	29237 / 6264 / 2943
Goodness-of-fit on F^2	1.092	1.073
Final R_1 / wR_2 [a]	0.0736 / 0.1982	0.0638 / 0.1986
Largest diff. peak/hole / e Å^{-3}	0.31/-0.35	0.90 / -0.57

[a] The value of *R*1 is based on 'observed' data with $I > 2\sigma(I)$; the value of w*R*2 is based on all data.

Simulations

The geometries of the cages *Z*•**Co•L*p***, *E*•**Co•L*m*** *Z*•**Co•L*m*** , and (BF_4^-)@*E*•**Co•L*p*** (Figures 10 and 18, main text, S50, S51d, and S52) as well as potential structures for **Co•L*m*•2,4** and **Co•L*m*•4,2** (Figure S53) were optimised with a semi-empirical tight-binding quantum chemical method denoted as GFN-xTB.[10] For these geometry optimisations the stand-alone program xtb was used.[11] The molecular mechanics simulations (MM2 force field) of *E*•**Co•L*p*** (Figure 7, main text, and S51a) and (BF_4^-)@*E*•**Co•L*p*** (Figure S51b) were performed using SCiGRESS (version FQ 3.4.5, Build 1669.13197, Serial No. 1620809473902 (copyright 2008-2024, Fujitsu Limited)).

***Co•L*p**

For *Z*•**Co•L*p*** (Figures 9 and S50) two potential structures exist with C_3 symmetry, arising from one metal centre with an opposing Δ/Λ (*Z*•**Co•L*p***•Δ/Λ) or *fac/mer* (*Z*•**Co•L*p***•*fm*) tris-chelate coordination geometry to the other three tris-chelate coordination geometries. The vertices and geometries of *Z*•**Co•L*p***•*fm* were based on a previously published X-ray crystal structure of a C_3 symmetric tetrahedron with the same metal-ligand coordination motif.[12] The one *fac* and three *mer* vertices were connected by six *Z*-*p*-azobenzenes using Avogadro[13] (an open-source molecular builder and visualisation tool, Version 1.2.0, http://avogadro.cc/) and the complete supramolecular structure subjected to modelling in xtb.

The all *fac* M_4L_6 tetrahedral isomer, *Z*•**Co•L*p***•Δ/Λ (Figure S50b), was based on the same previously published X-ray crystal structure.[12] The Δ *fac* vertex was copied four times and one of them mirrored using SCiGRESS (version FQ 3.4.5, Build 1669.13197, Serial No. 1620809473902 (copyright 2008-2024, Fujitsu Limited)). The one Λ and three Δ vertices were connected by six *Z*-*p*-azobenzenes using Avogadro and the complete supramolecular structure subsequently subjected to modelling in xtb.

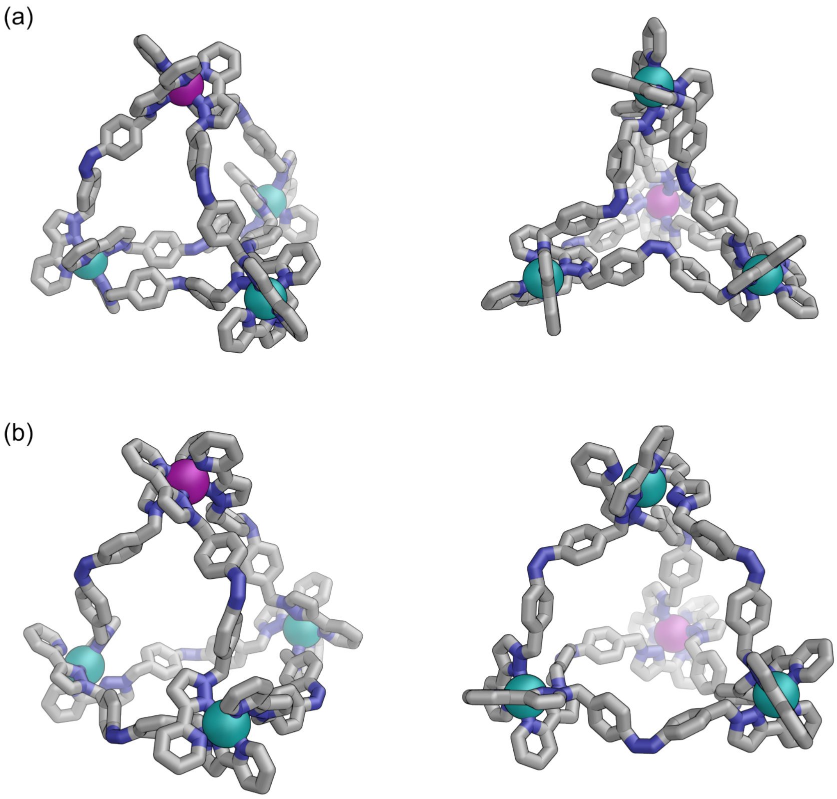

Figure S54. (a) GFN-xTB structures of two potential C_3 symmetric isomers of M_4L_6 structure *Z*•**Co•L*p***: (a) an M_4L_6 tetrahedron with one *fac* (purple) and three *mer* vertices (teal) (*Z*•**Co•L*p***•*fm*) and (b) an M_4L_6 tetrahedron with all *fac* vertices of different chiralities, one Λ (purple) and three Δ (teal) (*Z*•**Co•L*p***•Δ/Λ).

For *E*•**Co•L*p*** (Figure 7, main text, and S51a), an MM2 force field was needed, because dispersion interactions in GFN-xTB caused the helicate structure to collapse in order to allow for π-stacking between two of the ligands. Since ^{1}H NMR suggests approximate D_3 symmetry of the *E*•**Co•L*p*** helicate in solution, we reverted to a model which does not take dispersion interactions into account as strongly as GFN-xTB. The models of the vertices were based on the *fac* vertex obtained from a previously published X-ray crystal structure.[12] The cobalt(II) ions were set to octahedral geometry with sp^3d^2 hybridization to maintain Co–N bond angles. (BF_4^-)@*E*•**Co•L*p*** was simulated with a MM2 model for comparison (Figure S51b), showing that the flexibility of the ligands allows them to bend to accommodate the BF_4^- guest thereby reducing the Co—Co distance (Figure S51c). The MM2 coordinates for (BF_4^-)@*E*•**Co•L*p*** were also subjected to GFN-xTB which resulted in a similar geometry (Figure S51d).

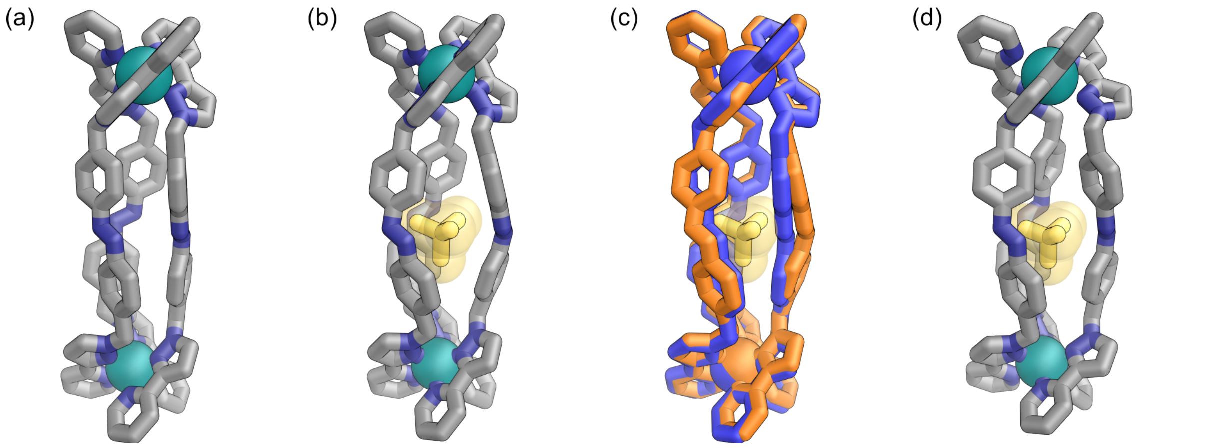

Figure S55. MM2 structures of (a) ***E*•Co•L*ᵖ***, (b) (BF_4^-)@***E*•Co•L*ᵖ***, and (c) the comparison of ***E*•Co•L*ᵖ*** (blue) and (BF_4^-)@***E*•Co•L*ᵖ*** (orange, with BF_4^- in yellow) showing the flexible ligands to bend slightly to accommodate the BF_4^- guest thereby reducing the Co—Co distance. (d) GFN-xTB structure of (BF_4^-)@***E*•Co•L*ᵖ***.

Co•L^m

The models of the vertices for E•Co•Lm and Z•Co•Lm were based on the vertices obtained from the X-ray crystal structure of ***E*•Co•L*ᵐ***, which were connected by the respective ligand structures using Avogadro and the complete supramolecular structures subsequently subjected to modelling in xtb. The modelled structure of ***Z*•Co•L*ᵐ*** is significantly expanded in comparison with the X-ray crystal structure of ***E*•Co•L*ᵐ***. To demonstrate that this is due to the lack of crystal packing forces in the molecular model, we subjected the coordinates of ***E*•Co•L*ᵐ*** obtained from its X-ray crystal structure to the same modelling method (GFN-xTB; Figure S52). Comparing the GFN-xTB structure of ***E*•Co•L*ᵐ*** to the X-ray crystal structure (Figure S52b) shows that subjection of the crystal structure coordinates to the GFN-xTB method causes the structure to unwind significantly, presumably due to the lack of crystal packing constraints. The structure of ***E*•Co•L*ᵐ*** seems to exhibit considerable flexibility, so that changing the ligand configuration from *E* to Z does not cause significant changes in the Co_4 framework (Figure S52c).

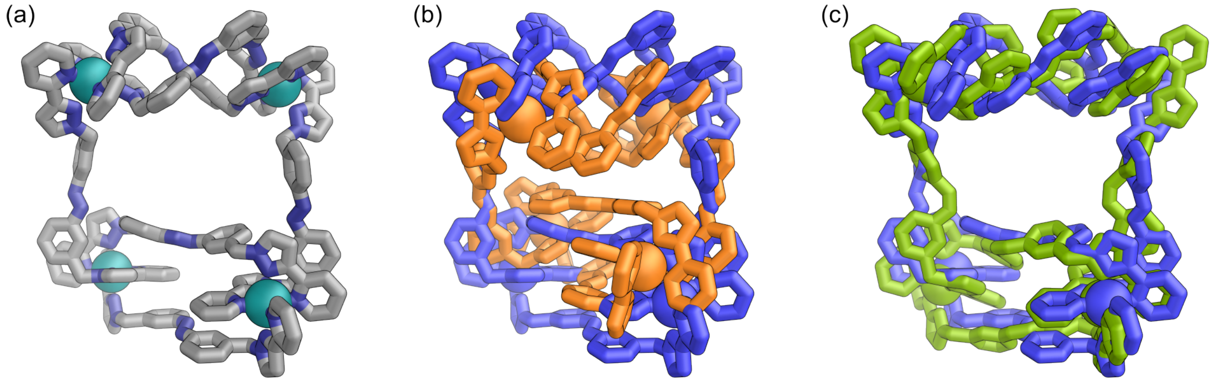

Figure S56. (a) GFN-xTB structure of ***E*•Co•L*ᵐ***. Comparison of GFN-xTB structure of ***E*•Co•L*ᵐ*** (blue) to (b) X-ray crystal structure of ***E*•Co•L*ᵐ*** (orange), that subjection of the crystal structure coordinates to the GFN-xTB method causes the structure to unwind significantly, and (c) GFN-xTB structure of ***Z*•Co•L*ᵐ*** (green), showing that the size of the Co_4 metallacycle barely changes upon *E*→*Z* isomerisation.

In cyclic bis-azobenzenes, virtually concerted switching of both azobenzenes can be observed,[14,15] with the *EZ* state being considerably less stable than the *EE* and *ZZ* states, respectively, leading to a short lifetime of the *EZ* state and a long thermal half-life of the *ZZ* state.[15] If the flexibility of the bis-azobenzene macrocycle is not sufficient, switching of the azo-units can be prevented entirely.[16] This literature precedence and the fact that we only seem to observe discrete structures with *Z*/*E* ratios of 0:6, 2:4, and 6:0, with an apparent fourth species likely possessing a 4:2 ratio, lead us to the assumption that the azo-units of **Co•L^{m}** would switch in pairs, i.e. the two helicate subunits and the two parallel connecting ligands, reducing the number of possible isomers for the structures with *Z*/*E* ligand mixtures, **Co•L^{m}•2,4** and **Co•L^{m}•4,2**, to three, respectively (Figure S53). We optimized the geometries of those possible isomers with GFN-xTB. Structures highlighted in yellow seem more likely to form.

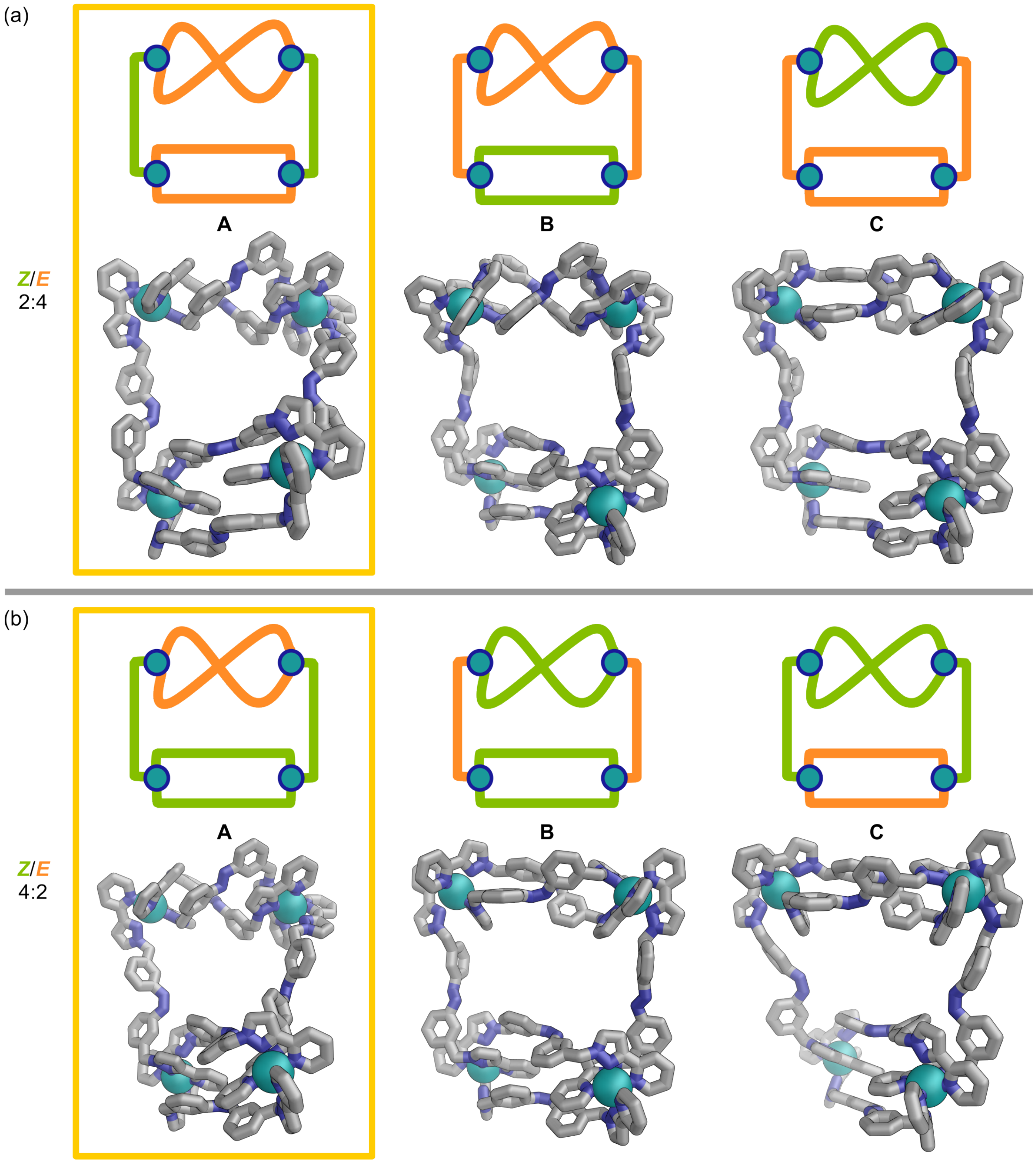

Figure S57. Possible structures (GFN-xTB) of **Co•L^{m}•2,4** and **Co•L^{m}•4,2**. Structures highlighted in yellow, seem more likely.

References

1. B. Jousselme, P. Blanchard, N. Gallego-Planas, E. Levillain, J. Delaunay, M. Allain, P. Richomme and J. Roncali, *Chem. Eur. J.,* 2003, **9**, 5297.
2. (a) A. J. Amoroso, A. M. W. Cargill Thompson, J. C. Jeffery, P. L. Jones, J. A. McCleverty and M. D. Ward, *J. Chem. Soc., Chem. Commun.,* 1994, 2751; (b) H. Brunner and T. Scheck, *Chem. Ber.,* 1992, **125**, 701; (c) Y. Lin and S. A. Lang, *J. Heterocycl. Chem.,* 1977, **14**, 345.
3. A. Stephenson and M. D. Ward, *RSC Adv.,* 2012, **2**, 10844.
4. C. Feldmeier, H. Bartling, E. Riedle and R. M. Gschwind, *J. Magn. Reson.*, 2013, **232**, 39.
5. R. Falkenburg, M. J. Notheis, G. Schnakenburg, L. K. S. von Krbek, *Org. Biomol. Chem.* 2023, **21**, 4993.
6. CrysAlisPro Software system (version 1.171.40.45a), Rigaku Oxford Diffraction, UK (2018).
7. D. R. Allan, H. Nowell, S. A. Barnett, M. R. Warren, A. Wilcox, J. Christensen, L. K. Saunders, A. Peach, M. T. Hooper, L. Zaja, S. Patel, L. Cahill, R. Marshall, S. Trimnell, A. J. Foster, T. Bates, S. Lay, M. A. Williams, P. V. Hathaway, G. Winter, M. Gerstel, R. W. Wooley, *Crystals,* 2017, **7**, 336.
8. O. V. Dolomanov, L. J. Bourhis, R. J. Gildea, J. A. K. Howard and H. Puschmann, *J. Appl. Cryst.,* 2009, **42**, 339.
9. G. M. Sheldrick, *Acta Cryst. A,* 2015, **71**, 3.
10. S. Grimme, C. Bannwarth, P. Sushkov, *J. Chem. Theory Comput.*, 2017, **13**, 1989-2009.
11. Please contact xtb@thch.uni–bonn.de to obtain the programme.
12. R. L. Paul, S. P. Argent, J. C. Jeffery, L. P. Harding, J. M. Lynam and M. D. Ward, *Dalton Trans.,* 2004, 3453.
13. M. D. Hanwell, D. E. Curtis, D. C. Lonie, T. Vandermeersch, E. Zurek, G. R. Hutchison *J. Cheminf.*, 2012, **4**.
14. M. Müri, K. C. Schuermann, L. De Cola, M. Mayor, *Eur. J. Org. Chem.*, 2009, 2562.
15. Y. Norikane, N. Tamaoki, *Org. Lett.*, 2004, **6**, 15, 2595–2598.
16. A. H. Heindl, L. Schweighauser, C. Logemann, H. A. Wegner, *Synthesis*, 2017, **49**, 2632.
